# Different interventions in preventing sufentanil-induced cough: a systematic review and network meta-analysis

**DOI:** 10.3389/fphar.2025.1619920

**Published:** 2025-11-19

**Authors:** Hao Liu, Yuewen He, Longfei Ding, Zhengze Zhang, Tong Wu, Ruogen Li, Yong Wang, Wuhua Ma

**Affiliations:** 1 Guangzhou University of Chinese Medicine, Guangzhou, Guangdong, China; 2 Department of Anesthesiology, The First Affiliated Hospital of Guangzhou University of Chinese Medicine, Guangzhou, Guangdong, China; 3 State Key Laboratory of Traditional Chinese Medicine Syndrome, The First Affiliated Hospital of Guangzhou University of Chinese Medicine, Guangzhou, Guangdong, China

**Keywords:** cough, dezocine, NMA (network meta-analysis), RCT, randomized controlled trial, sufentanil

## Abstract

**Background:**

Sufentanil-induced cough (SIC) is prevalent in anesthesia practice. A variety of interventions have been employed to prevent SIC. However, the optimal intervention remains elusive.

**Methods:**

A comprehensive search of the literature was conducted on the PubMed, Embase, Web of Science, Cochrane Library (CENTRAL) and China National Knowledge Infrastructure (CNKI) databases. The search was limited to publications prior to July 5, 2025. A network meta-analysis (NMA) was conducted using the R software. A Bayesian framework was employed for this NMA. Comparisons of competing models based on the deviance information criterion (DIC) were used to select the optimal model for NMA. The primary outcome is the overall incidence of SIC. The secondary outcomes included the incidence of mild SIC and moderate to severe SIC.

**Results:**

The NMA included 37 randomized controlled trials (RCTs) with 5,105 patients and 18 interventions. Pairwise meta-analysis results indicate that the intervention group significantly decreases the overall incidence of SIC (7.6% vs. 34.8%; OR 0.13; 95% CI 0.09 to 0.18; P < 0.0001; I^2^ = 53.0%), the incidence of mild SIC (4.0% vs. 13.0%; OR 0.28; 95% CI 0.22 to 0.35; P = 0.369; I^2^ = 5.7%), and the incidence of moderate to severe SIC (3.4% vs. 21.7%; OR 0.13; 95% CI 0.10 to 0.16; P = 0.040; I^2^ = 30.6%). NMA results suggested that nalbuphine, dezocine, and butorphanol significantly reduced the overall incidence of SIC, as well as the incidence of mild and moderate-to-severe SIC. Additionally, remifentanil and esketamine were effective in reducing both the overall incidence of SIC and the incidence of moderate to severe SIC. The use of a mechanical dropper was also effective in reducing the incidence of moderate to severe SIC.

**Conclusion:**

Three pharmacological interventions—nalbuphine, dezocine, and butorphanol significantly reduced the overall incidence of SIC, as well as the incidence of mild and moderate-to-severe SIC. Additionally, remifentanil and esketamine were effective in reducing the overall incidence of SIC and the incidence of moderate to severe SIC. The application of a mechanical dropper was also effective in reducing the incidence of moderate to severe SIC. The remaining interventions indicated a trend toward reducing SIC incidence; however, this was not statistically significant.

**Systematic Review:**

https://www.crd.york.ac.uk/PROSPERO/view/CRD42024581866 , PROSPERO (CRD42024581866)

## Introduction

Sufentanil is a potent mu-opioid receptor agonist characterized by rapid action, strong analgesic properties, prolonged duration, stable hemodynamics, and a high therapeutic index, making it an optimal choice for opioid analgesia during the induction of general anesthesia (Xue et al., 2008). SIC is prevalent in anesthesia practice, with some studies indicating an incidence rate as high as 64.7% (Xie et al., 2024). Coughs can vary in severity, with mild cases being self-limiting. However, severe cough may elevate the risk of aspiration pneumonia (Smith and Houghton, 2013) and postoperative nausea and vomiting (Peringathara and Robinson, 2016). In severe cases, it can elevate intracranial, intraocular, and intraabdominal pressures, potentially leading to various adverse effects in patients with high-risk comorbidities (Schug et al., 1992). Consequently, implementing effective interventions to prevent sufentanil-induced cough (SIC) in clinical settings is crucial for saving lives, improving quality of life, enhancing patient satisfaction, and optimizing healthcare resource utilization (Liu et al., 2023).

Various interventions have been suggested for the prevention of SIC, including pretreatment with drugs like dizocin (Liu et al., 2015), esketamine (Gao et al., 2024), nalmefene (Xie et al., 2024), and remifentanil (Zhang et al., 2024), as well as extended administration of induction drugs (Liu et al., 2019). Despite various interventions, the most effective intervention for preventing SIC remains uncertain.

Therefore, we conducted a meta-analysis that integrates existing randomized controlled trials and available interventions to assess the efficacy of various interventions in preventing SIC for clinical reference.

## Methods

### Study protocol

This systematic review was designed according to the Preferred Reporting Items for Systematic Review and Meta-Analyses extension statement for reviews incorporating NMA. The protocol of this review has been published in PROSPERO (ID: CRD42024581866). The PRISMA NMA checklist is available in the Supplementary Table S1.

### Search strategy

**FIGURE 1:**
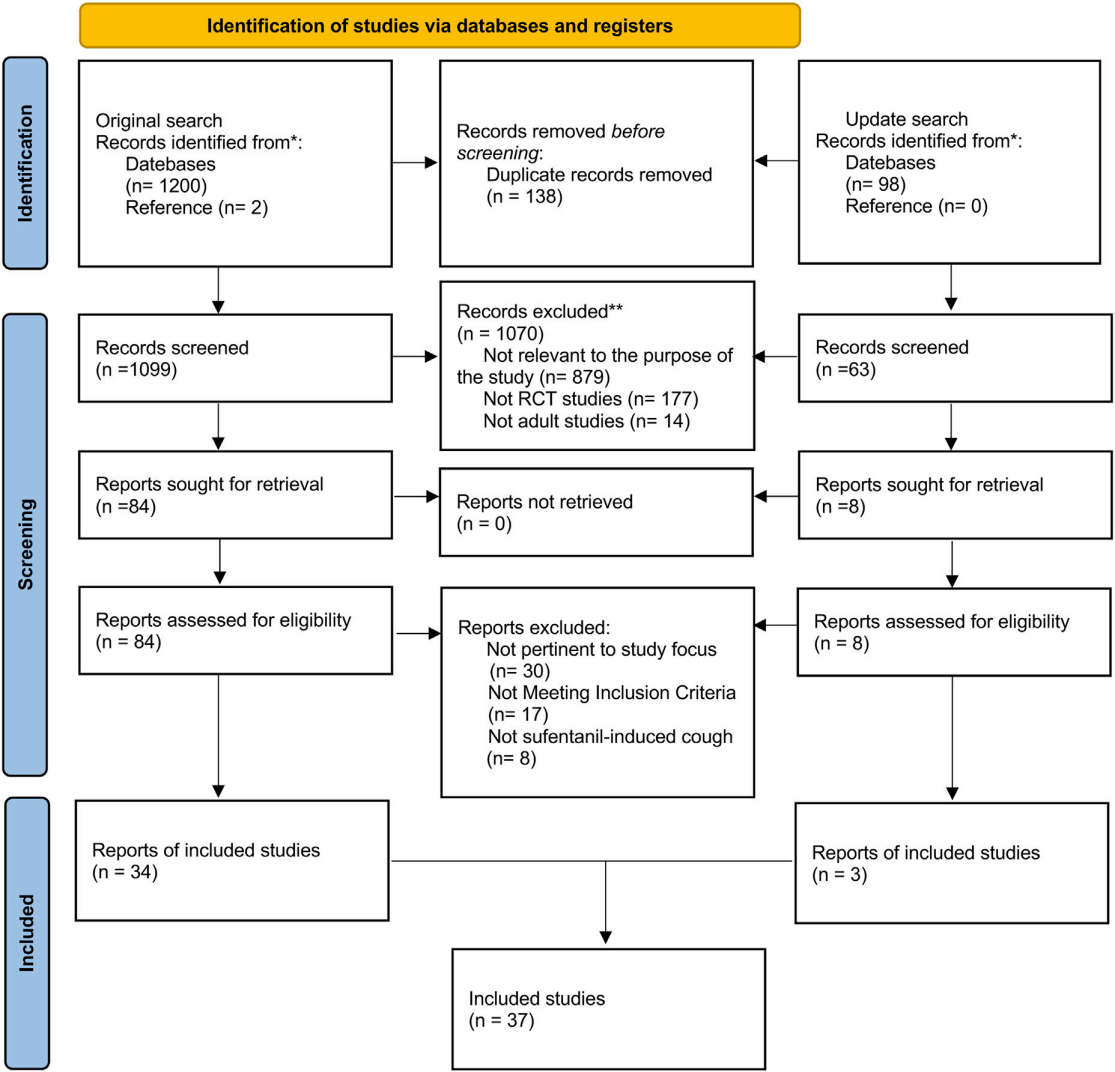
PRISMA flow diagram of the search strategy and included studies.

The search process was shown in PRISMA_2020_flow_diagram (Figure 1). Two researchers (H.L. and Y.W.H.) exhaustively searched studies published from inception to August 22, 2024, without language restriction in PubMed, Embase, Web of Science, Cochrane Library (CENTRAL), and China National Knowledge Infrastructure (CNKI) database. To ensure the inclusion of the most recent evidence, an updated systematic search was conducted, extending the original search window to July 5, 2025. The search formula was developed jointly by two independent researchers. W.H.M. was responsible for resolving any disputes during the process. Synonym searches and similar terms from critical meta-analysis determined the search terms for this NMA. Based on different databases, we would appropriately change the retrieval strategy, such as Mesh word and Publication Type and other limitations. In addition, we conducted a reference list search to enhance comprehensiveness (details in Supplementary Table S2).

### Study selection

The retrieved articles were managed by two researchers (H.L. and Y.W.H.) using EndNote X9 (Thomson Reuters, NY, USA). The process was as follows: first, we excluded all duplicates and incomplete studies. Subsequently, the titles, keywords, and abstracts were subjected to a review process, during which they were classified as “low correlation”, “moderate correlation” and “high correlation” in accordance with the established inclusion criteria. The investigators excluded all “low correlation” studies and examined the full text of the remaining studies, which were defined as “moderate correlation”, as well as all studies with “high correlation”. Finally, two reviewers identified the included literature based on the full text. When the results of the two researchers differed, the opinion of one researcher (W.H.M.) was used to reach a consensus. Figure 1 shows a screening process to illustrate the number of excluded studies at each stage.

### Eligibility criteria

For the inclusion of this NMA, studies had to meet the following criteria: published randomized controlled trials; at least two different interventions should be compared; at least one of four clinical outcomes (overall incidence of SIC, mild severity of SIC, moderate severity of SIC, and severe severity of SIC) should be evaluated; study participants are adults. The exclusion criteria were detailed as follows: non-randomized controlled trials; articles that did not compare at least two different interventions; and articles that did not evaluate at least one of four clinical outcomes; data could not be extracted from articles.

### Data extraction

Two investigators (H.L. and Y.W.H.) were independently responsible for data extraction, and W.H.M. and Y.W. adjudicated all disputes. We extracted the following data based on the characteristics of the included studies: Author, National, Year of publication, Language, Definition of cough severity, Age, ASA classification, Sex, Premedication, and Group information (Table 1). H.L. and Y.W.H. extracted and summarized the research data in Excel 2019, and W.H.M. was responsible for confirming the accuracy of the research data.

**TABLE 1:** Detailed information of individual studies enrolled in this network meta-analysis.

Study Country	Language	Age ASA Sex	Definition of cough severity	Sufentanil Concentration/Infusion Duration	Premedication	Group1 (dose, intervention, and number)	Group2 (dose, intervention, and number)	Group3 (dose, intervention, and number)	Group4 (dose, intervention, and number)
Gao et al. (2024) China	English	18∼70 years ASA I∼II Males and females	None: 0 Mild: 1–2 times Moderate: 3–5 times Severe: > 5 times	0.4 μg/kg 5s	No	0.15 mg/kg Esketamine 100	Equal volume NS 100	‐	‐
Lin et al. (2019) China	English	20∼70 years ASA I∼II Males and females	None: 0 Mild: 1–2 times Moderate: 3–5 times Severe: ≥5 times	0.5 μg/kg 5s	No	0.3 μg/kg Remifentanil 42	Equal volume NS 42	‐	
Tian et al. (2020) China	English	18∼65 years ASA I∼II Males and females	None: 0 Mild: 1–2 times Moderate: 3–4 times Severe: ≥5 times	0.5 μg/kg 3s	No	0.5 mg/kg Ketorolac tromethamine 45	Equal volume NS 45	‐	‐
Zhu et al. (2021) China	English	18∼50 years ASA I∼II Males and females	None: 0 Mild: 1–2 times Moderate: 3–5 times Severe: > 5 times	0.3 μg/kg 5s	No	0.5 μg/kg Dexmedetomidine 60	10mg Dexamethasone 60	Equal volume NS 60	‐
Xie et al. (2024) China	English	18∼65 years ASA I∼II Males and females	None: 0 Mild: 1–2 times Moderate: 3–4 times Severe: > 4 times	0.5 μg/kg 2s	NR	0.25 μg/kg Nalmefene 34	0.1 μg/kg Nalmefene 33	Equal volume NS 34	‐
Xu et al. (2024) China	English	18∼65 years ASA I∼II Males and females	None: 0 Mild: 1–2 times Moderate: 3–4 times Severe: ≥5 times	0.5 μg/kg 5s	No	2 μg/kg Afentanil 40	Equal volume NS 40	‐	‐
Qian et al. (2022) China	English	≥ 18 years ASA I∼II Females	None: 0 Mild: 1–2 times Moderate: 3–4 times Severe: ≥5 times	0.5 μg/kg 5s	No	1.25 μg/kg Naloxone 93	Equal volume NS 93	‐	‐
An et al. (2015) China	English	18∼65 years ASA I∼II Males and females	None: 0 Mild: 1–2 times Moderate: 3–4 times Severe: >5 times	1 μg/kg 5s	NR	50 mg/kg MgSO4 52	30 mg/kg MgSO4 55	Equal volume NS 53	-
Wang et al. (2020) China	English	18∼70 years ASA I∼II Males and females	None: 0 Mild: 1–2 times Moderate: 3–5 times Severe: >5 times	0.5 μg/kg 3s	NR	0.3 mg/kg Nalbuphine 105	Equal volume NS 105	‐	‐
Sun and Huang (2013) China	English	18∼58 years ASA I∼II Females	None: 0 Mild: 1–2 times Moderate: 3–5 times Severe: >5 times	0.5 μg/kg 3s	No	0.10 μg/kg Dexmedetomidine 60	0.25 μg/kg Dexmedetomidine 60	0.5 μg/kg Dexmedetomidine 60	Equal volume NS 60
Zhang et al. (2024) China	English	18∼58 years ASA I∼II Males and females	None: 0 Mild: 1–2 times Moderate: 3–5 times Severe: >5 times	0.5 μg/kg 5s	NR	0.5 μg /kg Remifentanil 60	Equal volume NS 60	‐	‐
Yin and Zhang (2019) China	English	18∼65 years ASA I∼II Males and females	None: 0 Mild: 1–2 times Moderate: 3–4 times Severe: ≥5 times	0.5 μg/kg 5s	NR	0.1 mg Butorphanol 40	1mg Butorphanol 40	Equal volume NS 40	‐
Zou et al. (2020) China	English	18∼65 years ASA I∼II Males and females	None: 0 Mild: 1–2 times Moderate: 3–5 times Severe: >5 times	0.3 μg/kg 5s	NR	5 μg Sufentanil 110	Equal volume NS 110	‐	‐
Zou et al. (2019) China	English	18∼65 years ASA I∼II Males and females	None: 0 Mild: 1–2 times Moderate: 3–5 times Severe: ≥5 times	0.4 μg/kg 5s	NR	1 mg/kg Tramadol 152	Equal volume NS 152	‐	‐
Liu et al. (2015) China	English	18∼70 years ASA I∼II Males and females	None: 0 Mild: 1–2 times Moderate: 3–5 times Severe: >5 times	0.5 μg/kg 3s	No	0.1 mg/kg Dezocine 185	Equal volume NS 185	‐	‐
Liu et al. (2019) China	English	18∼65 years ASA I∼II Males and females	None: 0 Mild: 1–2 times Moderate: 3–4 times Severe: ≥5 times	0.3 μg/kg mechanical dropper	No	0.3 μg/kg Mechanical dropper 100	0.3 μg/kg T-connector 100	‐	‐
Jie and Kai (2016) China	English	25∼53 years ASA I∼II Males and females	None: 0 Mild: 1–2 times Moderate: 3–4 times Severe: >5 times	0.3 μg/kg 3s	No	200 ug Salbutamol 40	Equal volume Saline aerosol 40	‐	‐
Qian (2024) China	English	18∼70 years ASA I∼II Males and females	None: 0 Mild: 1–2 times Moderate: 3–5 times Severe: >5 times	0.4 μg/kg 10s	NR	0.05 mg/kg Esketamine 118	Equal volume NS 118	‐	‐
Xie et al. (2025) China	English	≥ 18 years ASA III∼ IV Males and females	None: 0 Mild: 1–2 times Moderate: 3–5 times Severe: >5 times	2 μg/kg/3s	No	10 mg Dezocine 27	5mg Dezocine 27	Equal volume NS 27	
Zhou et al. (2025) China	English	18∼65 years ASA I∼II Males and females	None: 0 Mild: 1–2 times Moderate: 3–4 times,lasting < 5 s Severe: ≥ 5 times, lasting ≥ 5 s	0.5 μg/kg/5s	NR	0.2 mg/kg Esketamine 50	Equal volume NS 49	‐	‐
Chen (2019) China	Chinese	18∼65 years ASA I∼II Males and females	Grade 0: no choking Grade 1: choking duration ≤ 3 s Grade 2: choking duration > 3 s.	0.4 μg/kg 5s	NR	0.1 mg/kg Dezocine 30	1mg Butorphanol 40	Equal volume NS 40	‐
Wang and Qing (2015) China	Chinese	25∼65 years ASA I∼II Males and females	None: 0 Mild: 1–2 times Moderate: 3–5 times Severe: >5 times	0.3 μg/kg 5s	0.3mg scopolamine	0.1 mg/kg Dezocine 50	Equal volume NS 50	‐	‐
Li et al. (2015) China	Chinese	20∼55 years ASA I∼II Males and females	None: 0 Mild: 1–2 times Moderate: 3–4 times Severe: ≥5 timesNR	0.3 μg/kg 3s	NR	1 mg Butorphanol 41	Equal volume NS 41	‐	‐
He et al. (2020) China	Chinese	≥ 18 years ASA III∼IV Males and females	None: 0 Mild: 1–2 times Moderate: 3–4 times Severe: ≥5 times	2 μg/kg 3s	No	1 mg/kg Dezocine 20	Equal volume NS 20	‐	‐
Ding (2009) China	Chinese	18∼65 years ASA I∼II Males and females	None: 0 Mild: 1–2 times Moderate: 3–4 times Severe: ≥5 times	5 μg 2s	No	5 mg Dexamethasone 80	Equal volume NS 80	‐	‐
Shen et al. (2014) China	Chinese	20∼65 years ASA I∼II Females	None: 0 Mild: 1–2 times Moderate: 3–4 times Severe: ≥5 times	3 μg/kg 5s	NR	0.05 mg/kg Dezocine 60	Equal volume NS 60	‐	‐
Cao et al. (2020) China	Chinese	18∼60 years ASA I∼II Males and females	None: 0 Mild: 1–2 times Moderate: 3–4 times Severe: ≥5 times	0.4 μg/kg 5s	No	0.05 mg/kg Oxycodone 40	0.01 mg/kg Butorphanol 40	Equal volume NS 40	-
Yang et al. (2023) China	Chinese	Older ASA I∼II Males and females	None: 0 Mild: 1–2 times Moderate: 3–4 times Severe: ≥5 times	0.5 μg/kg NR	NR	0.1 mg/kg Nalbuphine 49	Equal volume NS 49	‐	‐
Sun and Guo (2024) China	Chinese	18∼65 years ASA I∼II Males and females	None: 0 Mild: 1times Moderate: 2–4 times Severe: ≥5 times	1.5 μg/kg 10s	NR	0.1 mg/kg Nalbuphine 48	Equal volume NS 48	‐	‐
Li and Li (2016) China	Chinese	23∼72 years ASA I∼II Males and females	None: 0 Mild: 1–2 times Moderate: 3–5 times Severe: >5 times	0.3 μg/kg 5s	0.3mg scopolamine	0.05 mg/kg Dezocine 80	0.1 mg/kg Dezocine 80	Equal volume NS 80	‐
Li (2014) China	Chinese	45∼60 years ASA I∼II Females	None: 0 Mild: 1–2 times Moderate: 3–4 times Severe: ≥5 times	0.5 μg/kg NR	No	0.2 μg/kg Nalmefene 25	Equal volume NS 25	‐	‐
Zhou et al. (2014) China	Chinese	18∼65 years ASA I∼II Males and females	None: 0 Mild: 1–2 times Moderate: 3–4 times Severe: ≥5 times	0.3 μg/kg 10s	0.1g phenobarbital sodium 0.5mg atropine	0.1 mg/kg Dezocine 30	Equal volume NS 30	‐	‐
Xu et al. (2014) China	Chinese	20∼65 years ASA I∼II Males and females	None: 0 Mild: 1–2 times Moderate: 3–4 times Severe: >5 times	0.5 μg/kg NR	No	0.1 mg/kg Dezocine 50	Equal volume NS 50	‐	‐
Zheng et al. (2019) China	Chinese	18∼65 years ASA I∼II Males and females	None: 0 Mild: 1–2 times Moderate: 3–4 times Severe: ≥5 times	0.4 μg/kg 6s	NR	5 mg Dezocine 50	Equal volume NS 50	‐	
Teng et al. (2020) China	Chinese	25∼45 years ASA I∼II Males and females	None: 0 Mild: 1–2 times Moderate: 3–4 times Severe: ≥5 times	0.5 μg/kg NR	NR	0.1 mg/kg Nalbuphine 40	Equal volume NS 44	‐	‐
You and Zeng (2023) China	Chinese	18∼65 years ASA I∼II Males and females	None: 0 Mild: 1–2 times Moderate: 3–4 times Severe: ≥5 times	0.5 μg/kg 5s	NR	0.1 μg/kg Sufentanil 44	Equal volume NS 40	‐	‐
Wang et al. (2014) China	Chinese	35∼65 years ASA I∼II Males and females	None: 0 Mild: 1–2 times Moderate: 3–4 times Severe: ≥5 times	0.5 μg/kg 10s	No	5 mg Dexamethasone 40	0.5 mg / kg Lidocaine 40	Equal volume NS 40	‐

Abbreviations: ASA, American Society of Anesthesiologists; NS, normal saline; NR: no record.

### Risk of bias assessment

**FIGURE 2:**
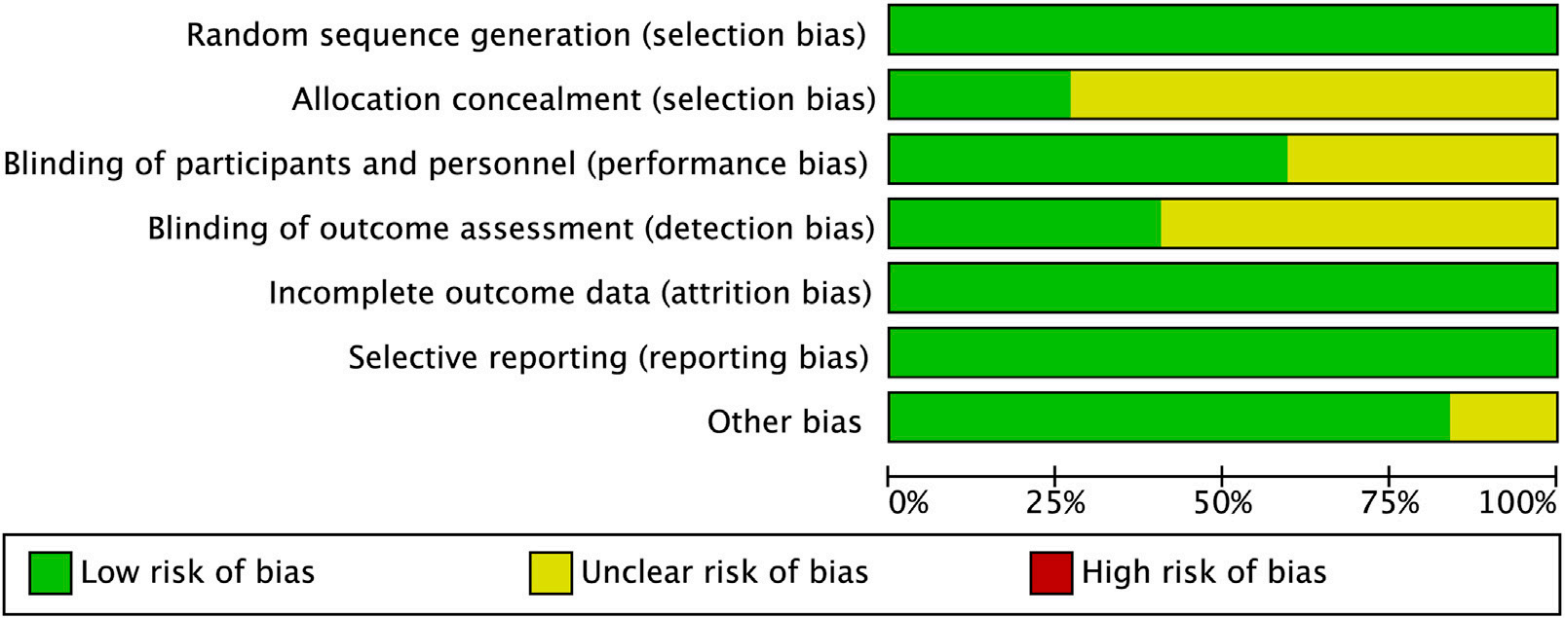
Risk of bias graph.

Two investigators (Z.Z.Z. and L.F.D.) independently assessed the risk of bias for each trial using Review Manager 5.4 (RevMan, The Cochrane Collaboration, Oxford, United Kingdom) according to the criteria outlined in the Cochrane Handbook for Systematic Reviews of Interventions. Based on the Cochrane Collaboration’s tool, RCT was defined as high risk, low risk, and unclear. The risk of bias summary is shown in Figure 2 and the Supplementary Figure S1.

### Outcomes

The primary outcome is the overall incidence of SIC. The secondary outcomes include the incidence of mild SIC and the incidence of moderate to severe SIC.

### Statistical analysis for pairwise meta-analysis

Two investigators (H.L. and Y.W.H.) are responsible for the statistical methodology. Meta packages of R (version 4.2.3) were applied to perform the pairwise meta-analysis of direct evidence by using random-effects models or fixed-effects models. For the pairwise meta-analysis, heterogeneity between studies was estimated by the *I*-squared (*I* ^2^) test and Cochran’s *Q* test. According to the Cochrane Collaboration Handbook, when moderate or high heterogeneity (*I* ^2^ > 50% and *P* < 0.05) was observed, a random-effects model was used; otherwise, a fixed-effects model was used. Furthermore, we chose meta packages of R (version 4.2.3) to generate funnel plots to assess publication bias. Evaluation methods include the plot of effect size centered at comparison-specific pooled effect and the Egger’s test to evaluate small sample effect. Where asymmetries were present, Duval and Tweedie’s trim-and-fill procedure was applied to estimate bias-corrected effects. When researchers disagree on the biased analysis of the same study, another researcher (W.H.M.) will make the decision.

### Statistical analysis for network meta-analysis

Two investigators (H.L. and Y.W.H.) are responsible for the statistical methodology. We constructed a network graph to evaluate the overall arrangement of the network evidence base. A network graph consists of nodes and lines. The nodes depict what we regarded as individual interventions. Meanwhile, the lines connecting different nodes represent the direct comparisons between the relevant interventions, and their thicknesses are proportional to the number of RCTs that studied the respective direct comparison.

For the NMA, the analysis was carried out in a Bayesian framework. The network estimates are obtained using the Markov chain Monte Carlo simulation method. For the analysis results of this study, two-tailed tests with *P* < 0.05 were defined as statistically significant. The metafor package (3.8.1) generated the NMA forest plot. Then, the DIC and potential scale reduced factor (PSRF) were calculated. DIC is widely used in the selection of Bayesian models. In general, a smaller DIC indicates a better fit for the model (Spiegelha et al., 2002). As for the PSRF, closer to 1, it means that the results have good convergence, and the consistency model can be considered robust (Supplementary Table S3). We evaluated the consistency between direct and indirect evidence through both local and global approaches. Analysis of heterogeneity and node-splitting methods, along with Q statistics to assess homogeneity and consistency, were applied for this purpose.

To rank the interventions, we reported the surface under the cumulative ranking curve (SUCRA) scores. For the outcomes in this NMA, a larger value of SUCRA means a better effect. Finally, meta-regression and subgroup analyses were conducted based on the duration of sufentanil injection, dosage of sufentanil injection, and ASA classification, and leave-one-out sensitivity analyses was employed to identify outliers and explore potential sources of heterogeneity.

### Certainty assessment of the evidence

Two independent investigators (H.L. and L.F.D.) assessed the quality of the evidence by using the standard Grading of Recommendations Assessment, Development and Evaluation (GRADE) method. The NMA findings were evaluated comprehensively in terms of risk of bias, indirectness, inconsistency, imprecision, and publication bias according to the GRADE methodology (Brignardello-Petersen et al., 2020). Additionally, the GRADE published framework was used to guide the development of summary of findings (SoF) tables to report comparative results for the NMA (Yepes-Nuñez et al., 2019).

## Results

### Literature search findings

A total of 1,202 studies were identified through initial searches of five databases and the reference list: PubMed (106), Embase (307), Web of Science (362), Cochrane Library (CENTRAL) (70), CNKI (355), and a reference list (2). Duplicate and ineligible trials were removed, followed by the exclusion of all trials categorized as “low correlation”, resulting in the inclusion of 84 RCTs. Following a joint screening of the full text of 84 trials by two reviewers, 34 trials (Xie et al., 2024; Liu et al., 2015; Gao et al., 2024; Zhang et al., 2024; Liu et al., 2019; Lin et al., 2019; Tian et al., 2020; Zhu et al., 2021; Xu et al., 2024; Qian et al., 2022; An et al., 2015; Wang et al., 2020; Sun and Huang, 2013; Yin and Zhang, 2019; Zou et al., 2019; Zou et al., 2020; Chen, 2019; Wang and Qing, 2015; Li et al., 2015) involving 4,689 patients were deemed eligible. An updated search identified three additional eligible studies (Qian et al., 2024; Xie et al., 2025; Zhou et al., 2025). In total, 37 studies involving 5,105 patients were included in the NMA. The search process is illustrated in the PRISMA 2020 flow diagram (Figure 1).

### Studies and patient characteristics

The intervention group comprised 2,815 patients undergoing pharmacological management or mechanical dropper, whereas the control group included 2,295 patients receiving normal saline. Eighteen distinct interventions were analyzed, comprising dezocine, nalbuphine, butorphanol, alfentanil, oxycodone, remifentanil, esketamine, ketorolac tromethamine, nalmefene, magnesium sulfate, salbutamol, dexmedetomidine, sufentanil, lidocaine, dexamethasone, naloxone, tramadol, and mechanical dropper (flow rate at 1 mL/s). Among them, dezocine (10 articles), butorphanol (5 articles), nalbuphine (3 articles), sufentanil (3 articles), esketamine (3 articles), dexamethasone (3 articles), dexmedetomidine (2 articles), nalmefene (2 articles), and remifentanil (2 articles) were discussed in more than 2 studies.

Table 1 summarizes the characteristics of the enrolled studies. All included studies reported the overall incidence of SIC. All studies reported on cough severity; however, two studies (Chen, 2019; Sun and Guo, 2024) were excluded due to discrepancies in the definition of cough severity, leading to a final inclusion of 35 articles for the analysis of SIC severity. To minimize bias due to varying definitions of moderate and severe SIC across studies, we will evaluate the incidence of mild SIC (1-2 instances of coughing) and the incidence of moderate to severe SIC (more than 2 instances of coughing).

### Assessment of risk of bias, consistency, and certainty of the evidence

The risk of bias assessments for 37 RCTs is shown in Figure 2 and the Supplementary Figure S1. Funnel plots were generated to assess the publication bias of the studies (Supplementary Figure S2). Moreover, the results of Egger’s test indicated that all outcomes had a risk of publication bias (Supplementary Table S5). Bias-corrected meta-analysis by trim-and-fill was performed separately for all outcomes (Supplementary Figures S2, S3).

No global or local inconsistencies were detected in any of the results (Supplementary Table S3; Supplementary Figure S6). The certainty of the evidence from the NMA was evaluated using the GRADE methodology (Supplementary Table S4). The absence of direct randomized controlled trial comparisons among certain interventions is noted. Consequently, inconsistency tests were not feasible. All indirect evidence was downgraded for inconsistency. Finally, the certainty of all evidence was between high and very low.

### Pairwise meta-analysis

During the initial phase of data analysis, a pairwise meta-analysis was conducted to compare the intervention group with the control group. The findings indicate that the intervention group significantly decreases the overall incidence of SIC (7.6% vs. 34.8%; OR 0.13; 95% CI 0.09 to 0.18; P < 0.0001; I^2^ = 53.0%), the incidence of mild SIC (4.0% vs. 13.0%; OR 0.28; 95% CI 0.22 to 0.35; P = 0.369; I^2^ = 5.7%), and the incidence of moderate to severe SIC (3.4% vs. 21.7%; OR 0.13; 95% CI 0.10 to 0.16; P = 0.040; I^2^ = 30.6%). Significant heterogeneity was observed in the primary outcome (*I* ^*2*^ = 53.0%) (Supplementary Figure S3). Consequently, after excluding outliers (Yin and Zhang, 2019; Qian et al., 2024; Ding, 2009; Xu et al., 2014), heterogeneity was substantially reduced (Supplementary Figure S3). Meta-regression analysis and 留一法 were performed however, the source of the heterogeneity could not be identified (Supplementary Table S3). The funnel plots and results from Egger’s test demonstrate the presence of publication bias across all outcomes. A bias-corrected meta-analysis was conducted utilizing the trim-and-fill method for all outcomes, confirming the effectiveness of the intervention group in preventing SIC (Supplementary Figures S2, S3).

### Network meta-analysis

**FIGURE 3:**
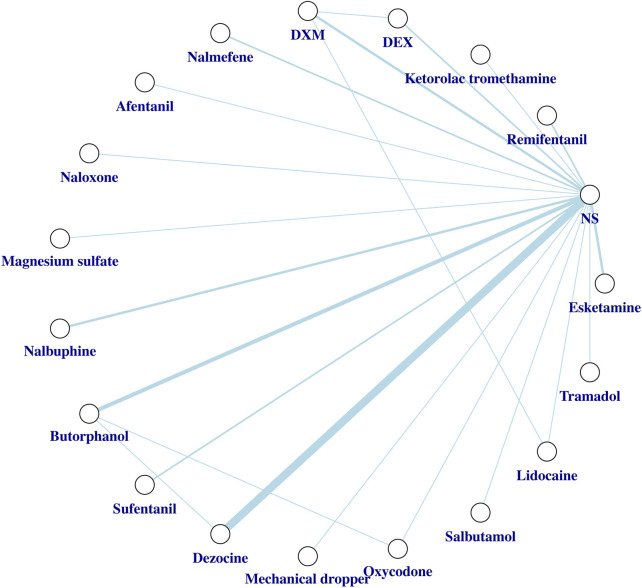
Network plot of the network meta-analysis for the overall incidence of SIC. Network meta-analysis plot comparing different interventions. Each node represents what we consider to be a single intervention. The lines represent direct comparisons between interventions, and their thickness is proportional to the number of clinical trials included in each comparison. Abbreviations: DEX, Dexmedetomidine; DXM, Dexamethasone; NS, normal saline; SIC, sufentanil-induced cough.

**FIGURE 4:**
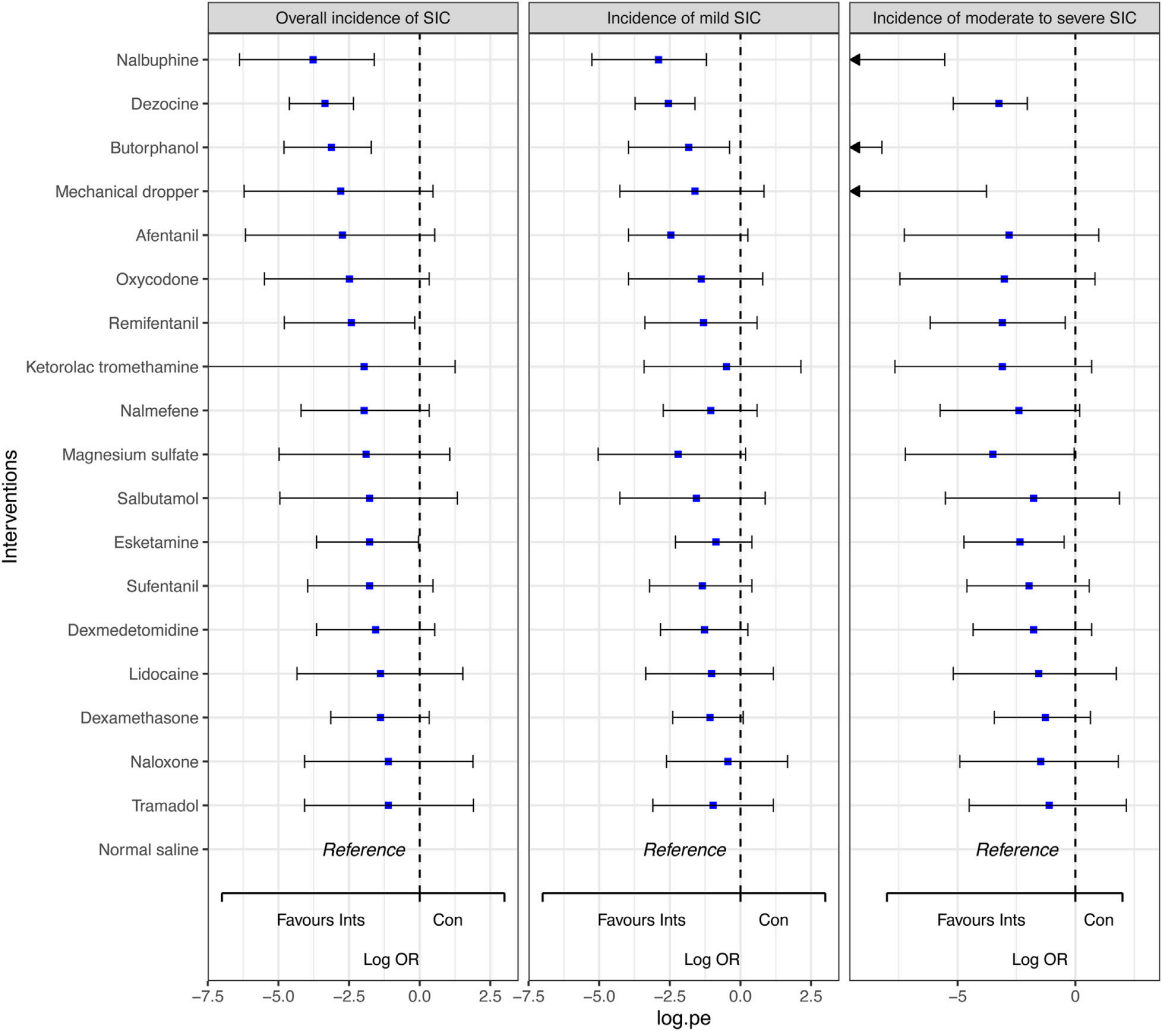
Forest plots for the outcomes with the comparative network effect sizes of all interventions. Blue squares represent the estimated network effect sizes. Black bars represent the 95% credible intervals (95% CrIs). Abbreviations: SIC, sufentanil-induced cough.

**FIGURE 5:**
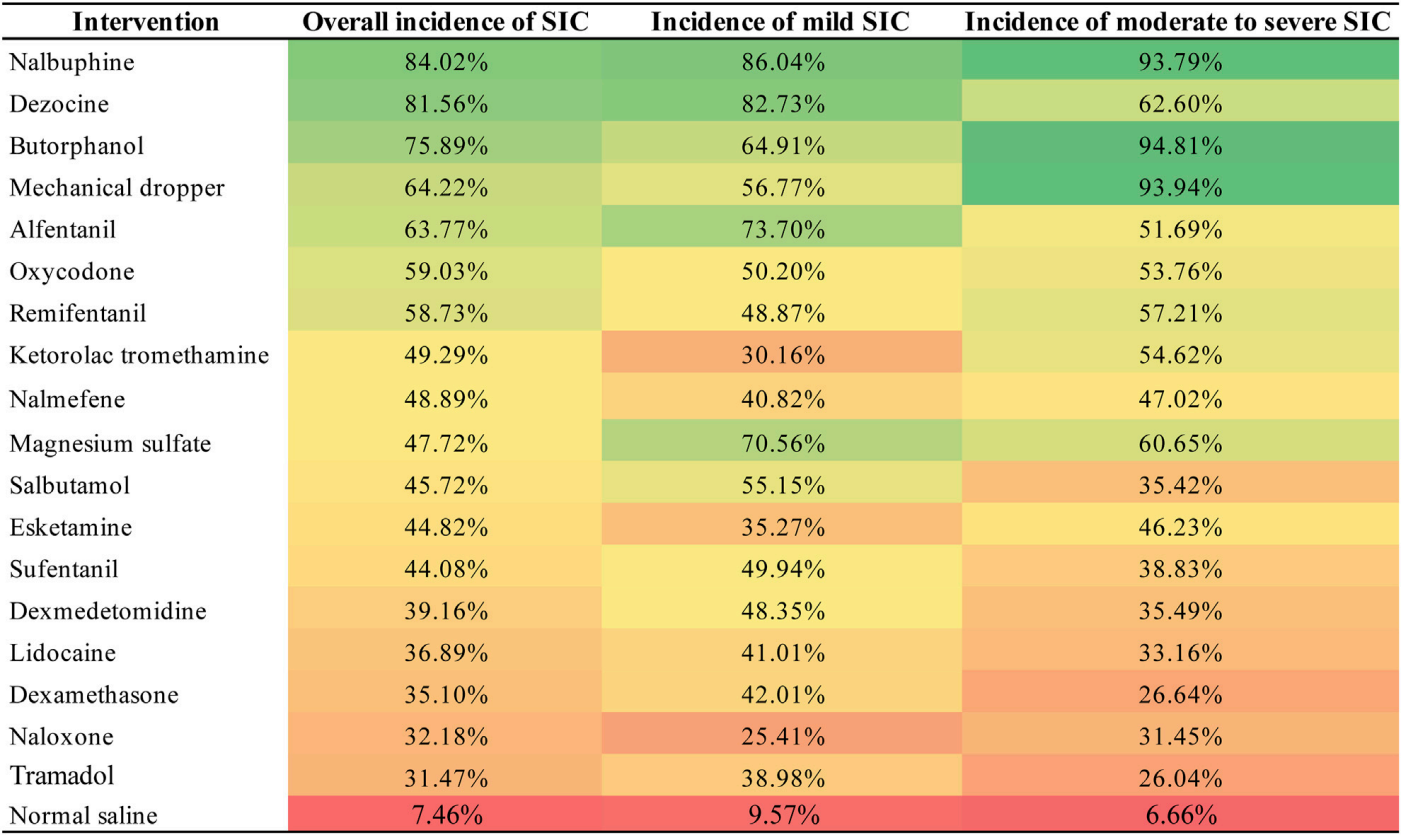
Heat map of surface under the cumulative ranking curve (SUCRA) values of each intervention for the outcomes. “Heat map” of surface under every intervention’s cumulative ranking curve (SUCRA) values for all outcomes. Values can range from 0% to 100%, and the higher the percentage, the greater the likelihood that the intervention is ranked first or in the top ranks. The highest SUCRA values are green, lowest ones are red. Abbreviations: SIC, sufentanil-induced cough.

Network plot for the overall incidence of SIC is shown in Figure 3. Network plots for the incidence of mild SIC and moderate to severe SIC are shown in Supplementary Figure S4. We did not detect global inconsistency and therefore used the consistency model for network estimation. Effect model selection based on DIC results (Supplementary Table S3). The pooled effect sizes derived from the network estimation and the SUCRA values and the ranking of the interventions for some outcomes are presented in Figures 4, 5, and Supplementary Figure S5.

In comparison to normal saline, dezocine (OR 0.03, 95% CI 0.01 to 0.10; high-quality evidence), nalbuphine (OR 0.02, 95% CI 0.00 to 0.20; high-quality evidence), butorphanol (OR 0.04, 95% CI 0.01 to 0.18; high-quality evidence),remifentanil (OR 0.09, 95% CI 0.01 to 0.84; moderate-quality evidence), and esketamine (OR 0.17, 95% CI 0.03 to 0.96; moderate-quality evidence) significantly decreased the overall incidence of SIC. Similarly, dezocine (OR 0.08, 95% CI 0.02 to 0.20; high-quality evidence), nalbuphine (OR 0.06, 95% CI 0.01 to 0.30; high-quality evidence), and butorphanol (OR 0.16, 95% CI 0.02 to 0.68; moderate-quality evidence) significantly reduced the incidence of mild SIC. Dezocine, nalbuphine, butorphanol, mechanical dropper, remifentanil, magnesium sulfate and esketamine significantly reduced the incidence of moderate to severe SIC. While the other interventions indicated a trend in decreasing the incidence of SIC, the results were not statistically significant.

## Discussion

Sufentanil, a fentanyl analogue, is an opioid analgesic with high selectivity for the μ-receptor site (Monk et al., 1988). It is commonly utilized for inducing general anesthesia in clinical settings because of its reliable analgesic effectiveness, lack of histamine release, and minimal effects on hemodynamics (Xu et al., 2024). Coughing is a prevalent side effect associated with sufentanil during the induction of general anesthesia. Coughing serves as a defensive reflex (Yin et al., 2017), with receptors extensively located throughout the bronchial tree and present in lesser quantities in regions including the ear, paranasal sinuses, pleura, diaphragm, pericardium, and esophagus (Andrani et al., 2019; Polverino et al., 2012). Coughing results from the activation of a complex reflex arc, serving to prevent foreign objects from entering the respiratory tract and to clear excessive bronchial secretions, thereby playing a crucial protective role for the airways and lungs (Andrani et al., 2019). The increase in pressure within the coelomic cavity due to coughing, which encompasses intracranial, intraocular, and intra-abdominal pressure, may result in significant negative consequences for critically ill patients (Tian et al., 2020; Sun and Huang, 2013).

The mechanism of the Opioid-induced cough (OIC) is complex and currently not well understood. Various hypotheses have been proposed by researchers, including the receptor hypothesis, vagal excitation hypothesis, β-arrestin signaling pathway, citric acid, and opioid receptor hypothesis, among others (Chen et al., 2020). Moreover, OIC is influenced by several factors, including the types of opioids, dosage, concentration, and the individual physical conditions of patients (El Baissari et al., 2014; Shu et al., 2016). Various interventions are currently employed to prevent SIC in clinical settings. The absence of direct comparisons among various interventions presents challenges for clinical physicians in selecting the most effective therapeutic drug for patients undergoing general anesthesia. Consequently, we have produced the initial article on the prevention of SIC as an NMA to serve as a reference for future clinical research.

The study comprised 18 intervention measures. Traditional pair-wise meta-analyses demonstrated that these interventions effectively reduce the incidence and severity of SIC. The NMA results indicated that pre-treatment with dezocine, nalbuphine, and butorphanol significantly decreases the overall incidence of SIC, as well as the incidence of mild and moderate to severe SIC. Remifentanil and a mechanical dropper are effective solely in reducing the incidence of moderate to severe SIC. No significant statistical significance was observed for the remaining intervention measures. The three drugs that are efficacious in preventing all SIC outcomes—dezocine, nalbuphine, and butorphanol—are all mixed agonist-antagonists (Ye et al., 2022; Gunion et al., 2004; Commiskey et al., 2005). This sparks our curiosity. Previous studies have identified dezocine as a partial agonist of mu receptors. The role of kappa-receptors remains a subject of debate (Wang et al., 2017). Liu et al. (2014) suggest that dezocine acts as an antagonist of kappa-receptors, whereas Wang et al. (2018) propose it functions as a partial agonist. Nalbuphine exhibits solely antagonist effects at mu-receptors, while it demonstrates an activating agonist effect at kappa receptors (Gunion et al., 2004). Butorphanol functions as a partial agonist at mu-receptors and as an agonist at kappa-receptors (Ji et al., 2020). Future research necessitates a deeper investigation into the occurrence mechanisms of OIC and the mechanisms of drug action. In addition, some research indicates that combination therapies, including ketamine with dexmedetomidine (Saleh et al., 2014), ketamine with dexamethasone (Safavi et al., 2013), and dexmedetomidine with midazolam (Yu et al., 2012), may improve the preventive effects of monotherapy for OIC. At present, there is a lack of research assessing the efficacy of combination therapy in the prevention of SIC.

The dosage of the intervention medication requires careful consideration. The absence of consensus regarding the dosage of intervention drugs for SIC prevention leads to variability in the dosages of the same intervention drugs utilized in this study. Table 1. Li and Li (2017) reported no significant difference in the effectiveness of preventing SIC between the low-dose dexmedetomidine group (0.05 mg/kg) and the high-dose group (0.1 mg/kg). However, the high-dose group exhibited a greater incidence of adverse reactions. Xie et al. (2024) found that the incidence of SIC in the low-dose nalmefene group (0.25 μg/kg) was 30.3%, compared to 14.7% in the high-dose group. Previous studies (Cheng et al., 2016; Pandey et al., 2005; Zhou et al., 2019) on the impact of intervention drug dosages on OIC (69, 70, 71) also indicate a correlation between the dosage of pretreatment drugs and their preventive effects. In summary, there is still a significant gap regarding the optimal dosage of SIC preventive medications, and future research should focus more on the impact of drug dosage on the effectiveness of SIC prevention.

This study represents the inaugural NMA utilizing randomized controlled trials to compare the efficacy of various interventions in the prevention of SIC. The preventive effects of 18 interventions were analyzed and compared through traditional pairwise comparisons and NMA, addressing gaps in direct comparisons of specific interventions. Simultaneously, this study presents certain limitations. Different doses of the same drug utilized in various studies may introduce biases in the research outcomes. Secondly, the number of articles on specific intervention measures is restricted, and additional RCT studies are required in the future to address this. Ultimately, the constraints of meta-analysis permit the extraction of only a restricted volume of data from the selected articles. The analysis of our results focused solely on effectiveness, neglecting factors such as dosage variations, adverse effects, timing of drug administration, and cost-benefit considerations.

## Conclusion

Our results indicate that pretreatment with dezocine, nalbuphine, and butorphanol significantly reduced the overall incidence of SIC, as well as the incidence of mild and moderate-to-severe SIC. Additionally, remifentanil and mechanical dropper were effective in reducing the incidence of moderate to severe SIC. The remaining interventions indicated a trend toward reducing SIC incidence; however, this was not statistically significant. Future research should prioritize the conduct of additional high-quality randomized controlled trials to enhance current results and establish the optimal dosage of intervention medications.

## Data Availability

The datasets presented in this study can be found in online repositories. The names of the repository/repositories and accession number(s) can be found in the article/Supplementary Material.
