# Supplementary material for: Different interventions in preventing sufentanil-induced cough: a systematic review and network meta-analysis
Source: Front Pharmacol. 2025 Nov 19;16:1619920. doi: 10.3389/fphar.2025.1619920 (PMC12673273; doi:10.3389/fphar.2025.1619920)

Different interventions in preventing sufentanil-induced cough: a systematic review and network meta-analysis

Additional Files

Tables

Table S1 PRISMA NMA Checklist.

Table S2 Search strategy

Table S3 Network meta-analysis: model fit details.

Table S4 Summary of the GRADE assessment of NMA.

Table S5 Result of heterogeneity test and meta-regression for conventional pair comparisons.

Figures

Figure S1 Risk of bias summary review authors' judgment about each risk of bias item for included trials.

Figure S2 Funnel plot for the conventional pairwise meta-analysis.

Figure S3 Forest plots of conventional pairwise meta-analysis.

Figure S4 Network plot for the secondary outcomes in the meta-analysis.

Figure S5 League table.

Figure S6 Results of local inconsistency and heterogeneity analyses.

Figure S7 Gelman–Rubin diagnostic plots

Table S1 PRISMA NMA Checklist of Items to Include When Reporting a Systematic Review Involving a Network Meta-analysis

Section/Topic	Item #	Checklist Item	Reported on Page #
**TITLE**			
Title	1	Identify the report as a systematic review *incorporating a network meta-analysis (or related form of meta-analysis).*	Title
**ABSTRACT**			
Structured summary	2	Provide a structured summary including, as applicable: **Background:** main objectives **Methods:** data sources; study eligibility criteria, participants, and interventions; study appraisal; and *synthesis methods, such as network meta-analysis.* **Results:** number of studies and participants identified; summary estimates with corresponding confidence/credible intervals; *treatment rankings may also be discussed. Authors may choose to summarize pairwise comparisons against a chosen treatment included in their analyses for brevity.* **Discussion/Conclusions:** limitations; conclusions and implications of findings. **Other:** primary source of funding; systematic review registration number with registry name.	Abstract
**INTRODUCTION**			
Rationale	3	Describe the rationale for the review in the context of what is already known*, including mention of why a network meta-analysis has been conducted.*	Introduction
Objectives	4	Provide an explicit statement of questions being addressed, with reference to participants, interventions, comparisons, outcomes, and study design (PICOS).	Introduction
**METHODS**			
Protocol and registration	5	Indicate whether a review protocol exists and if and where it can be accessed (e.g., Web address); and, if available, provide registration information, including registration number.	Methods
Eligibility criteria	6	Specify study characteristics (e.g., PICOS, length of follow-up) and report characteristics (e.g., years considered, language, publication status) used as criteria for eligibility, giving rationale. *Clearly describe eligible treatments included in the treatment network, and note whether any have been clustered or merged into the same node (with justification).*	Methods
Information sources	7	Describe all information sources (e.g., databases with dates of coverage, contact with study authors	Methods

		to identify additional studies) in the search and date last searched.	
Search	8	Present full electronic search strategy for at least one database, including any limits used, such that it could be repeated.	Methods
Study selection	9	State the process for selecting studies (i.e., screening, eligibility, included in systematic review, and, if applicable, included in the meta-analysis).	Methods
Data collection process	10	Describe method of data extraction from reports (e.g., piloted forms, independently, in duplicate) and any processes for obtaining and confirming data from investigators.	Methods
Data items	11	List and define all variables for which data were sought (e.g., PICOS, funding sources) and any assumptions and simplifications made.	Methods
**Geometry of the network**	**S1**	Describe methods used to explore the geometry of the treatment network under study and potential biases related to it. This should include how the evidence base has been graphically summarized for presentation, and what characteristics were compiled and used to describe the evidence base to readers.	Methods
Risk of bias within individual studies	12	Describe methods used for assessing risk of bias of individual studies (including specification of whether this was done at the study or outcome level), and how this information is to be used in any data synthesis.	Methods
Summary measures	13	State the principal summary measures (e.g., risk ratio, difference in means). *Also describe the use of additional summary measures assessed, such as treatment rankings and surface under the cumulative ranking curve (SUCRA) values, as well as modified approaches used to present summary findings from meta-analyses.*	Methods
Planned methods of analysis	14	Describe the methods of handling data and combining results of studies for each network meta-analysis. This should include, but not be limited to:<br>• *Handling of multi-arm trials;*<br>• *Selection of variance structure;*<br>• *Selection of prior distributions in Bayesian analyses; and*<br>• *Assessment of model fit.*	Methods
**Assessment of Inconsistency**	**S2**	Describe the statistical methods used to evaluate the agreement of direct and indirect evidence in the treatment network(s) studied. Describe efforts taken to address its presence when found.	Methods
Risk of bias across studies	15	Specify any assessment of risk of bias that may affect the cumulative evidence (e.g., publication bias, selective reporting within studies).	Methods
Additional analyses	16	Describe methods of additional analyses if done, indicating which were pre-specified. This may include, but not be limited to, the following:<br>• Sensitivity or subgroup analyses;<br>• Meta-regression analyses;<br>• *Alternative formulations of the treatment network; and*<br>• *Use of alternative prior distributions for Bayesian analyses (if applicable).*	Methods

RESULTS†

Study selection	17	Give numbers of studies screened, assessed for eligibility, and included in the review, with reasons for exclusions at each stage, ideally with a flow diagram.	Results
**Presentation of network structure**	**S3**	Provide a network graph of the included studies to enable visualization of the geometry of the treatment network.	Results, Figure 3, Additional file: Figure S4
**Summary of network geometry**	**S4**	Provide a brief overview of characteristics of the treatment network. This may include commentary on the abundance of trials and randomized patients for the different interventions and pairwise comparisons in the network, gaps of evidence in the treatment network, and potential biases reflected by the network structure.	Results
Study characteristics	18	For each study, present characteristics for which data were extracted (e.g., study size, PICOS, follow-up period) and provide the citations.	Results, Tbale 1
Risk of bias within studies	19	Present data on risk of bias of each study and, if available, any outcome level assessment.	Figure 2, Additional files Table S2
Results of individual studies	20	For all outcomes considered (benefits or harms), present, for each study: 1) simple summary data for each intervention group, and 2) effect estimates and confidence intervals. *Modified approaches may be needed to deal with information from larger networks.*	Results
Synthesis of results	21	Present results of each meta-analysis done, including confidence/credible intervals. *In larger networks, authors may focus on comparisons versus a particular comparator (e.g. placebo or standard care), with full findings presented in an appendix. League tables and forest plots may be considered to summarize pairwise comparisons.* If additional summary measures were explored (such as treatment rankings), these should also be presented.	Results, Figure 4
**Exploration for inconsistency**	**S5**	Describe results from investigations of inconsistency. This may include such information as measures of model fit to compare consistency and inconsistency models, *P* values from statistical tests, or summary of inconsistency estimates from different parts of the treatment network.	Results
Risk of bias across studies	22	Present results of any assessment of risk of bias across studies for the evidence base being studied.	Results
Results of additional analyses	23	Give results of additional analyses, if done (e.g., sensitivity or subgroup analyses, meta-regression analyses*, alternative network geometries studied, alternative choice of prior distributions for Bayesian analyses,* and so forth).	Results

DISCUSSION

Summary of evidence	24	Summarize the main findings, including the strength of evidence for each main outcome; consider their relevance to key groups (e.g., healthcare providers, users, and policy-makers).	Discussion
Limitations	25	Discuss limitations at study and outcome level (e.g., risk of bias), and at review level (e.g.,	Discussion

		incomplete retrieval of identified research, reporting bias). *Comment on the validity of the assumptions, such as transitivity and consistency. Comment on any concerns regarding network geometry (e.g., avoidance of certain comparisons).*	
Conclusions	26	Provide a general interpretation of the results in the context of other evidence, and implications for future research.	Discussion
**FUNDING**			
Funding	27	Describe sources of funding for the systematic review and other support (e.g., supply of data); role of funders for the systematic review. This should also include information regarding whether funding has been received from manufacturers of treatments in the network and/or whether some of the authors are content experts with professional conflicts of interest that could affect use of treatments in the network.	Funding

PICOS = population, intervention, comparators, outcomes, study design.

* Text in italics indicates wording specific to reporting of network meta-analyses that has been added to guidance from the PRISMA statement.

† Authors may wish to plan for use of appendices to present all relevant information in full detail for items in this section.

Table S2 Search strategy

Number	Search for
#1	Esketamine[Supplementary Concept] OR Esketamine[Title/Abstract] OR Ketamine[Mesh] OR Ketalar[Title/Abstract] OR Ketanest[Title/Abstract] OR Ketaset[Title/Abstract]
#2	Nalmefene[Supplementary Concept] OR Nalmefene[Title/Abstract]
#3	Alfentanil[MeSH Terms] OR (Alfentanil[Title/Abstract] OR Alfentanyl[Title/Abstract] OR Alfenta[Title/Abstract] OR Rapifen[Title/Abstract] OR alfentanil hydrochloride[Title/Abstract]
#4	Lidocaine[MeSH Terms] OR (Lidocaine[Title/Abstract] OR Lignocaine[Title/Abstract] OR lidocaine hydrocarbonate[Title/Abstract] OR Xyloneural[Title/Abstract] OR Xylocaine[Title/Abstract]
#5	Oxycodone[MeSH Terms] OR Oxycodone[MeSH Terms] OR (Oxycodone[Title/Abstract] OR Dihydrohydroxycodeinone[Title/Abstract] OR Dihydrone[Title/Abstract] OR Oxycodeinon[Title/Abstract] OR Oxycone[Title/Abstract] OR Oxycontin[Title/Abstract]
#6	Ketorolac Tromethamine[MeSH Terms] OR (Ketorolac Tromethamine[Title/Abstract] OR Toradol[Title/Abstract] OR Acular[Title/Abstract]
#7	Sufentanil[MeSH Terms] OR (Sufentanil[Title/Abstract] OR Sulfentanyl[Title/Abstract] OR Sufenta[Title/Abstract]
#8	Butorphanol[MeSH Terms] OR (Butorphanol[Title/Abstract] OR Beforal[Title/Abstract] OR Stadol[Title/Abstract] OR Moradol[Title/Abstract] OR Torbugesic[Title/Abstract]
#9	Remifentanil[MeSH Terms] OR Remifentanil[Title/Abstract] OR Ultiva[Title/Abstract] OR remifentanil hydrochloride[Title/Abstract]
#10	dezocine[Supplementary Concept] OR dezocine[Title/Abstract]
#11	Dexmedetomidine[MeSH Terms] OR dexmedetomidine hydrochloride[Title/Abstract] OR Precedex[Title/Abstract] OR Igalmi[Title/Abstract] OR Sileo[Title/Abstract] OR Dexdor[Title/Abstract] OR Dexdomitor[Title/Abstract]
#12	Nalbuphine[MeSH Terms] OR (Nubain[Title/Abstract] OR Nalbuphine[Title/Abstract]
#13	Dexamethasone[MeSH Terms] OR (Dexamethasone[Title/Abstract] OR Hexadecadrol[Title/Abstract] OR Methylfluorprednisolone[Title/Abstract] OR Oradexon[Title/Abstract] OR Millicorten[Title/Abstract] OR Hexadrol[Title/Abstract] OR Dexasone[Title/Abstract] OR Decaspray[Title/Abstract] OR Decameth[Title/Abstract] OR Decaject[Title/Abstract] OR Dexpak[Title/Abstract] OR Maxidex[Title/Abstract]
#14	Albuterol[MeSH Terms] OR (Albuterol[Title/Abstract] OR Salbutamol[Title/Abstract] OR Ventolin[Title/Abstract] OR Sultanol[Title/Abstract] OR Proventil[Title/Abstract]
#15	Magnesium[MeSH Terms] OR Magnesium[Title/Abstract]
#16	Propofol[MeSH Terms] OR (Propofol[Title/Abstract] OR Disoprofol[Title/Abstract] OR Diprivan[Title/Abstract] OR Disoprivan[Title/Abstract] OR Aquafol[Title/Abstract] OR Fresofol[Title/Abstract] OR Ivofol[Title/Abstract] OR Recofol[Title/Abstract]
#17	Naloxone[MeSH Terms] OR Naloxone[Title/Abstract] OR Narcan[Title/Abstract] OR Narcanti[Title/Abstract] OR Nalone[Title/Abstract]

#18	mechanical dropper[Title/Abstract] OR induction regimen[Title/Abstract]
#19	sufentanil induced cough[Title/Abstract] OR (sufentanil-induced[All Fields] AND bucking[Title/Abstract]) OR (sufentanil-induced[All Fields] AND choking[Title/Abstract])
#20	opioid induced cough[Title/Abstract] OR (opioid-induced[All Fields] AND choking[Title/Abstract]) OR (opioid-induced[All Fields] AND bucking[Title/Abstract]) OR SIC[Title/Abstract])
#21	#1 OR #2 OR #3 OR #4 OR #5 OR #6 OR #7 OR #8 OR #9 OR #10 OR #11 OR #12 OR #13 OR #14 OR #15 OR #16 OR #17 OR #18
#22	#19 OR #20
#23	#21 AND #22

Table S3 Network meta-analysis: model fit details

	**Consistency model**		**Inconsistency model**
	**Random-effects model**	**Fixed-effects model**	
**The overall incidence of SIC**			
Dbar	81.94	147.30	81.26
pD	70.78	56.27	70.56
DIC	**152.72**	203.57	152.18
I^2	6%	48%	6%
PSRF	1.0022	1.0010	1.0013
**The incidence of mild SIC**			
Dbar	75.64	88.39	75.78
pD	62.46	55.01	62.61
DIC	**138.09**	143.40	138.39
I^2	5%	19%	5%
PSRF	1.0022	1.0017	1.0032
**The incidence of moderate to severe SIC**			
Dbar	67.54	88.39	67.41
pD	59.27	55.01	59.18
DIC	**126.81**	143.40	126.59
I^2	0%	19%	0%
PSRF	1.0065	1.0017	1.0066

Dbar: posterior mean of deviance; DIC: deviance information criteria; pD: effective number of parameters or leverage.
* In general, the smaller the DIC, the better the model fit is indicated. Therefore, random effects models were chosen in our NMA.

Table S4 A. Summary of findings table and GRADE assessment of NMA for the overall incidence of sufentanil-induced cough.

Comparison	Number of studies	Within-study Bias [1]	Reporting bias	Indirectness[2]	Imprecision [3]	Heterogeneity [4]	Incoherence	Confidence rating	Reason(s) for downgrading
Alfentanil: Normal saline	1	No concerns	Low risk	No concerns	No concerns	Major concerns	No concerns	moderate	Heterogeneity
Butorphanol: Dezocine	1	Some concerns	Low risk	No concerns	Major concerns	No concerns	No concerns	moderate	Imprecision
Butorphanol: Normal saline	4	Some concerns	Low risk	No concerns	No concerns	No concerns	No concerns	High	-
Butorphanol: Oxycodone	1	Some concerns	Low risk	No concerns	Major concerns	No concerns	No concerns	moderate	Imprecision
Dexamethasone: Dexmedetomidine	1	Some concerns	Low risk	No concerns	Major concerns	No concerns	No concerns	moderate	Imprecision
Dexamethasone: Lidocaine	1	Some concerns	Low risk	No concerns	Major concerns	No concerns	No concerns	moderate	Imprecision
Dexamethasone: Normal saline	3	Some concerns	Low risk	No concerns	No concerns	Major concerns	No concerns	moderate	Heterogeneity
Dexmedetomidine:	2	Some concerns	Low risk	No concerns	No concerns	Major concerns	No concerns	moderate	Heterogeneity

Normal saline									
Dezocine:<br><br>Normal saline	9	Some concerns	Low risk	No concerns	No concerns	No concerns	No concerns	High	-
Esketamine:<br><br>Normal saline	1	No concerns	Low risk	No concerns	No concerns	Some concerns	No concerns	moderate	Heterogeneity
Ketorolac tromethamine:<br><br>Normal saline	1	Some concerns	Low risk	No concerns	Some concerns	Some concerns	No concerns	low	Imprecision, Heterogeneity
Lidocaine:<br><br>Normal saline	1	Some concerns	Low risk	No concerns	Major concerns	No concerns	No concerns	moderate	Imprecision
Mechanical dropper:<br><br>Normal saline	1	Some concerns	Low risk	No concerns	No concerns	Some concerns	No concerns	moderate	Heterogeneity
MgSO4:<br><br>Normal saline	1	No concerns	Low risk	No concerns	No concerns	Major concerns	No concerns	moderate	Heterogeneity
Nalbuphine:<br><br>Normal saline	1	No concerns	Low risk	No concerns	No concerns	No concerns	No concerns	High	-
Nalmefene:<br><br>Normal saline	2	No concerns	Low risk	No concerns	No concerns	Major concerns	No concerns	moderate	Heterogeneity

Naloxone: Normal saline	1	Some concerns	Low risk	No concerns	Major concerns	No concerns	No concerns	moderate	Imprecision
Normal saline: Oxycodone	1	Some concerns	Low risk	No concerns	No concerns	Major concerns	No concerns	moderate	Heterogeneity
Normal saline: Remifentanil	2	Some concerns	Low risk	No concerns	No concerns	No concerns	No concerns	moderate	-
Normal saline: Salbutamol	1	Some concerns	Low risk	No concerns	Major concerns	No concerns	No concerns	moderate	Imprecision
Normal saline: Sufentani;	2	Some concerns	Low risk	No concerns	No concerns	Major concerns	No concerns	moderate	Heterogeneity
Normal saline: Tramadol	1	Some concerns	Low risk	No concerns	Major concerns	No concerns	No concerns	moderate	Imprecision
Alfentanil: Butorphanol	0	Some concerns	Low risk	Major concerns	Major concerns	No concerns	No concerns	low	Indirectness, Imprecision
Alfentanil: Dexamethasone	0	Some concerns	Low risk	Major concerns	Major concerns	No concerns	No concerns	low	Indirectness, Imprecision
Alfentanil: Dexmedetomidine	0	Some concerns	Low risk	Major concerns	Major concerns	No concerns	No concerns	low	Indirectness, Imprecision

Alfentanil:<br><br>Dezocine	0	No concerns	Low risk	Major concerns	Major concerns	No concerns	No concerns	low	Indirectness, Imprecision
Alfentanil:<br><br>Esketamine	0	No concerns	Low risk	Major concerns	Major concerns	No concerns	No concerns	low	Indirectness, Imprecision
Alfentanil:<br><br>Ketorolac tromethamine	0	Some concerns	Low risk	Major concerns	Major concerns	No concerns	No concerns	low	Indirectness, Imprecision
Alfentanil:<br><br>Lidocaine	0	Some concerns	Low risk	Major concerns	Major concerns	No concerns	No concerns	low	Indirectness, Imprecision
Alfentanil:<br><br>Mechanical dropper	0	Some concerns	Low risk	Major concerns	Major concerns	No concerns	No concerns	low	Indirectness, Imprecision
Alfentanil:<br><br>MgSO4	0	No concerns	Low risk	Major concerns	Major concerns	No concerns	No concerns	low	Indirectness, Imprecision
Alfentanil:<br><br>Nalbuphine	0	No concerns	Low risk	Major concerns	Major concerns	No concerns	No concerns	low	Indirectness, Imprecision
Alfentanil:<br><br>Nalmefene	0	No concerns	Low risk	Major concerns	Major concerns	No concerns	No concerns	low	Indirectness, Imprecision
Alfentanil:<br><br>Naloxone	0	Some concerns	Low risk	Major concerns	Major concerns	No concerns	No concerns	low	Indirectness, Imprecision

Alfentanil: Oxycodone	0	Some concerns	Low risk	Major concerns	Major concerns	No concerns	No concerns	low	Indirectness, Imprecision
Alfentanil: Remifentanil	0	Some concerns	Low risk	Major concerns	Major concerns	No concerns	No concerns	low	Indirectness, Imprecision
Alfentanil: Salbutamol	0	Some concerns	Low risk	Major concerns	Major concerns	No concerns	No concerns	low	Indirectness, Imprecision
Alfentanil: Sufentani;	0	Some concerns	Low risk	Major concerns	Major concerns	No concerns	No concerns	low	Indirectness, Imprecision
Alfentanil: Tramadol	0	Some concerns	Low risk	Major concerns	Major concerns	No concerns	No concerns	low	Indirectness, Imprecision
Butorphanol: Dexamethasone	0	Some concerns	Low risk	Major concerns	Major concerns	No concerns	No concerns	low	Indirectness, Imprecision
Butorphanol: Dexmedetomidine	0	Some concerns	Low risk	Major concerns	Major concerns	No concerns	No concerns	low	Indirectness, Imprecision
Butorphanol: Esketamine	0	Some concerns	Low risk	Major concerns	Major concerns	No concerns	No concerns	low	Indirectness, Imprecision
Butorphanol: Ketorolac tromethamine	0	Some concerns	Low risk	Major concerns	Major concerns	No concerns	No concerns	low	Indirectness, Imprecision

Butorphanol:<br><br>Lidocaine	0	Some concerns	Low risk	Major concerns	Major concerns	No concerns	No concerns	low	Indirectness, Imprecision
Butorphanol:<br><br>Mechanical dropper	0	Some concerns	Low risk	Major concerns	Major concerns	No concerns	No concerns	low	Indirectness, Imprecision
Butorphanol:<br><br>MgSO4	0	Some concerns	Low risk	Major concerns	Major concerns	No concerns	No concerns	low	Indirectness, Imprecision
Butorphanol:<br><br>Nalbuphine	0	Some concerns	Low risk	Major concerns	Major concerns	No concerns	No concerns	low	Indirectness, Imprecision
Butorphanol:<br><br>Nalmefene	0	Some concerns	Low risk	Major concerns	Major concerns	No concerns	No concerns	low	Indirectness, Imprecision
Butorphanol:<br><br>Naloxone	0	Some concerns	Low risk	Major concerns	Major concerns	No concerns	No concerns	low	Indirectness, Imprecision
Butorphanol:<br><br>Remifentanil	0	Some concerns	Low risk	Major concerns	Major concerns	No concerns	No concerns	low	Indirectness, Imprecision
Butorphanol:<br><br>Salbutamol	0	Some concerns	Low risk	Major concerns	Major concerns	No concerns	No concerns	low	Indirectness, Imprecision
Butorphanol:<br><br>Sufentani;	0	Some concerns	Low risk	Major concerns	Major concerns	No concerns	No concerns	low	Indirectness, Imprecision

Butorphanol:<br><br>Tramadol	0	Some concerns	Low risk	Major concerns	Major concerns	No concerns	No concerns	low	Indirectness, Imprecision
Dexamethasone:<br><br>Dezocine	0	Some concerns	Low risk	Major concerns	No concerns	Major concerns	No concerns	low	Indirectness, Heterogeneity
Dexamethasone:<br><br>Esketamine	0	Some concerns	Low risk	Major concerns	Major concerns	No concerns	No concerns	low	Indirectness, Imprecision
Dexamethasone:<br><br>Ketorolac tromethamine	0	Some concerns	Low risk	Major concerns	Major concerns	No concerns	No concerns	low	Indirectness, Imprecision
Dexamethasone:<br><br>Mechanical dropper	0	Some concerns	Low risk	Major concerns	Major concerns	No concerns	No concerns	low	Indirectness, Imprecision
Dexamethasone:<br><br>MgSO4	0	Some concerns	Low risk	Major concerns	Major concerns	No concerns	No concerns	low	Indirectness, Imprecision
Dexamethasone:<br><br>Nalbuphine	0	Some concerns	Low risk	Major concerns	Some concerns	Some concerns	No concerns	very low	Indirectness, Imprecision, Heterogeneity
Dexamethasone:<br><br>Nalmefene	0	Some concerns	Low risk	Major concerns	Major concerns	No concerns	No concerns	low	Indirectness, Imprecision
Dexamethasone:<br><br>Naloxone	0	Some concerns	Low risk	Major concerns	Major concerns	No concerns	No concerns	low	Indirectness, Imprecision

Dexamethasone: Oxycodone	0	Some concerns	Low risk	Major concerns	Major concerns	No concerns	No concerns	low	Indirectness, Imprecision
Dexamethasone: Remifentanil	0	Some concerns	Low risk	Major concerns	Major concerns	No concerns	No concerns	low	Indirectness, Imprecision
Dexamethasone: Salbutamol	0	Some concerns	Low risk	Major concerns	Major concerns	No concerns	No concerns	low	Indirectness, Imprecision
Dexamethasone: Sufentani;	0	Some concerns	Low risk	Major concerns	Major concerns	No concerns	No concerns	low	Indirectness, Imprecision
Dexamethasone: Tramadol	0	Some concerns	Low risk	Major concerns	Major concerns	No concerns	No concerns	low	Indirectness, Imprecision
Dexmedetomidine: Dezocine	0	Some concerns	Low risk	Major concerns	Some concerns	Some concerns	No concerns	very low	Indirectness, Imprecision, Heterogeneity
Dexmedetomidine: Esketamine	0	Some concerns	Low risk	Major concerns	Major concerns	No concerns	No concerns	low	Indirectness, Imprecision
Dexmedetomidine: Ketorolac tromethamine	0	Some concerns	Low risk	Major concerns	Major concerns	No concerns	No concerns	low	Indirectness, Imprecision
Dexmedetomidine: Lidocaine	0	Some concerns	Low risk	Major concerns	Major concerns	No concerns	No concerns	low	Indirectness, Imprecision

Dexmedetomidine: Mechanical dropper	0	Some concerns	Low risk	Major concerns	Major concerns	No concerns	No concerns	low	Indirectness, Imprecision
Dexmedetomidine: MgSO4	0	Some concerns	Low risk	Major concerns	Major concerns	No concerns	No concerns	low	Indirectness, Imprecision
Dexmedetomidine: Nalbuphine	0	Some concerns	Low risk	Major concerns	Major concerns	No concerns	No concerns	low	Indirectness, Imprecision
Dexmedetomidine: Nalmefene	0	Some concerns	Low risk	Major concerns	Major concerns	No concerns	No concerns	low	Indirectness, Imprecision
Dexmedetomidine: Naloxone	0	Some concerns	Low risk	Major concerns	Major concerns	No concerns	No concerns	low	Indirectness, Imprecision
Dexmedetomidine: Oxycodone	0	Some concerns	Low risk	Major concerns	Major concerns	No concerns	No concerns	low	Indirectness, Imprecision
Dexmedetomidine: Remifentanil	0	Some concerns	Low risk	Major concerns	Major concerns	No concerns	No concerns	low	Indirectness, Imprecision
Dexmedetomidine: Salbutamol	0	Some concerns	Low risk	Major concerns	Major concerns	No concerns	No concerns	low	Indirectness, Imprecision
Dexmedetomidine: Sufentani;	0	Some concerns	Low risk	Major concerns	Major concerns	No concerns	No concerns	low	Indirectness, Imprecision

Dexmedetomidine:<br><br>Tramadol	0	Some concerns	Low risk	Major concerns	Major concerns	No concerns	No concerns	low	Indirectness, Imprecision
Dezocine:<br><br>Esketamine	0	No concerns	Low risk	Major concerns	Major concerns	No concerns	No concerns	low	Indirectness, Imprecision
Dezocine:<br><br>Ketorolac tromethamine	0	Some concerns	Low risk	Major concerns	Major concerns	No concerns	No concerns	low	Indirectness, Imprecision
Dezocine:<br><br>Lidocaine	0	Some concerns	Low risk	Major concerns	Major concerns	No concerns	No concerns	low	Indirectness, Imprecision
Dezocine:<br><br>Mechanical dropper	0	Some concerns	Low risk	Major concerns	Major concerns	No concerns	No concerns	low	Indirectness, Imprecision
Dezocine:<br><br>MgSO4	0	No concerns	Low risk	Major concerns	Major concerns	No concerns	No concerns	low	Indirectness, Imprecision
Dezocine:<br><br>Nalbuphine	0	No concerns	Low risk	Major concerns	Major concerns	No concerns	No concerns	low	Indirectness, Imprecision
Dezocine:<br><br>Nalmefene	0	Some concerns	Low risk	Major concerns	Major concerns	No concerns	No concerns	low	Indirectness, Imprecision
Dezocine:<br><br>Naloxone	0	Some concerns	Low risk	Major concerns	Some concerns	Some concerns	No concerns	low	Indirectness, Imprecision, Heterogeneity

Dezocine: Oxycodone	0	Some concerns	Low risk	Major concerns	Major concerns	No concerns	No concerns	low	Indirectness, Imprecision
Dezocine: Remifentanil	0	Some concerns	Low risk	Major concerns	Major concerns	No concerns	No concerns	low	Indirectness, Imprecision
Dezocine: Salbutamol	0	Some concerns	Low risk	Major concerns	Major concerns	No concerns	No concerns	low	Indirectness, Imprecision
Dezocine: Sufentani;	0	Some concerns	Low risk	Major concerns	Major concerns	No concerns	No concerns	low	Indirectness, Imprecision
Dezocine: Tramadol	0	Some concerns	Low risk	Major concerns	No concerns	Major concerns	No concerns	low	Indirectness, Heterogeneity
Esketamine: Ketorolac tromethamine	0	Some concerns	Low risk	Major concerns	Major concerns	No concerns	No concerns	low	Indirectness, Imprecision
Esketamine: Lidocaine	0	Some concerns	Low risk	Major concerns	Major concerns	No concerns	No concerns	low	Indirectness, Imprecision
Esketamine: Mechanical dropper	0	Some concerns	Low risk	Major concerns	Major concerns	No concerns	No concerns	low	Indirectness, Imprecision
Esketamine: MgSO4	0	No concerns	Low risk	Major concerns	Major concerns	No concerns	No concerns	low	Indirectness, Imprecision

Esketamine:<br><br>Nalbuphine	0	No concerns	Low risk	Major concerns	Major concerns	No concerns	No concerns	low	Indirectness, Imprecision
Esketamine:<br><br>Nalmefene	0	No concerns	Low risk	Major concerns	Major concerns	No concerns	No concerns	low	Indirectness, Imprecision
Esketamine:<br><br>Naloxone	0	Some concerns	Low risk	Major concerns	Major concerns	No concerns	No concerns	low	Indirectness, Imprecision
Esketamine:<br><br>Oxycodone	0	Some concerns	Low risk	Major concerns	Major concerns	No concerns	No concerns	low	Indirectness, Imprecision
Esketamine:<br><br>Remifentanil	0	Some concerns	Low risk	Major concerns	Major concerns	No concerns	No concerns	low	Indirectness, Imprecision
Esketamine:<br><br>Salbutamol	0	Some concerns	Low risk	Major concerns	Major concerns	No concerns	No concerns	low	Indirectness, Imprecision
Esketamine:<br><br>Sufentani;	0	Some concerns	Low risk	Major concerns	Major concerns	No concerns	No concerns	low	Indirectness, Imprecision
Esketamine:<br><br>Tramadol	0	Some concerns	Low risk	Major concerns	Major concerns	No concerns	No concerns	low	Indirectness, Imprecision
Ketorolac tromethamine:<br><br>Lidocaine	0	Some concerns	Low risk	Major concerns	Major concerns	No concerns	No concerns	low	Indirectness, Imprecision

Ketorolac tromethamine:<br><br>Mechanical dropper	0	Some concerns	Low risk	Major concerns	Major concerns	No concerns	No concerns	low	Indirectness, Imprecision
Ketorolac tromethamine:<br><br>MgSO4	0	Some concerns	Low risk	Major concerns	Major concerns	No concerns	No concerns	low	Indirectness, Imprecision
Ketorolac tromethamine:<br><br>Nalbuphine	0	Some concerns	Low risk	Major concerns	Major concerns	No concerns	No concerns	low	Indirectness, Imprecision
Ketorolac tromethamine:<br><br>Nalmefene	0	Some concerns	Low risk	Major concerns	Major concerns	No concerns	No concerns	low	Indirectness, Imprecision
Ketorolac tromethamine:<br><br>Naloxone	0	Some concerns	Low risk	Major concerns	Major concerns	No concerns	No concerns	low	Indirectness, Imprecision
Ketorolac tromethamine:<br><br>Oxycodone	0	Some concerns	Low risk	Major concerns	Major concerns	No concerns	No concerns	low	Indirectness, Imprecision
Ketorolac tromethamine:<br><br>Remifentanil	0	Some concerns	Low risk	Major concerns	Major concerns	No concerns	No concerns	low	Indirectness, Imprecision

Ketorolac tromethamine:<br><br>Salbutamol	0	Some concerns	Low risk	Major concerns	Major concerns	No concerns	No concerns	low	Indirectness, Imprecision
Ketorolac tromethamine:<br><br>Sufentani;	0	Some concerns	Low risk	Major concerns	Major concerns	No concerns	No concerns	low	Indirectness, Imprecision
Ketorolac tromethamine:<br><br>Tramadol	0	Some concerns	Low risk	Major concerns	Major concerns	No concerns	No concerns	low	Indirectness, Imprecision
Lidocaine:<br><br>Mechanical dropper	0	Some concerns	Low risk	Major concerns	Major concerns	No concerns	No concerns	low	Indirectness, Imprecision
Lidocaine:<br><br>MgSO4	0	Some concerns	Low risk	Major concerns	Major concerns	No concerns	No concerns	low	Indirectness, Imprecision
Lidocaine:<br><br>Nalbuphine	0	Some concerns	Low risk	Major concerns	Major concerns	No concerns	No concerns	low	Indirectness, Imprecision
Lidocaine:<br><br>Nalmefene	0	Some concerns	Low risk	Major concerns	Major concerns	No concerns	No concerns	low	Indirectness, Imprecision
Lidocaine:<br><br>Naloxone	0	Some concerns	Low risk	Major concerns	Major concerns	No concerns	No concerns	low	Indirectness, Imprecision

Lidocaine: Oxycodone	0	Some concerns	Low risk	Major concerns	Major concerns	No concerns	No concerns	low	Indirectness, Imprecision
Lidocaine: Remifentanil	0	Some concerns	Low risk	Major concerns	Major concerns	No concerns	No concerns	low	Indirectness, Imprecision
Lidocaine: Salbutamol	0	Some concerns	Low risk	Major concerns	Major concerns	No concerns	No concerns	low	Indirectness, Imprecision
Lidocaine: Sufentani;	0	Some concerns	Low risk	Major concerns	Major concerns	No concerns	No concerns	low	Indirectness, Imprecision
Lidocaine: Tramadol	0	Some concerns	Low risk	Major concerns	Major concerns	No concerns	No concerns	low	Indirectness, Imprecision
Mechanical dropper: MgSO4	0	Some concerns	Low risk	Major concerns	Major concerns	No concerns	No concerns	low	Indirectness, Imprecision
Mechanical dropper: Nalbuphine	0	Some concerns	Low risk	Major concerns	Major concerns	No concerns	No concerns	low	Indirectness, Imprecision
Mechanical dropper: Nalmefene	0	Some concerns	Low risk	Major concerns	Major concerns	No concerns	No concerns	low	Indirectness, Imprecision
Mechanical dropper: Naloxone	0	Some concerns	Low risk	Major concerns	Major concerns	No concerns	No concerns	low	Indirectness, Imprecision

Mechanical dropper: Oxycodone	0	Some concerns	Low risk	Major concerns	Major concerns	No concerns	No concerns	low	Indirectness, Imprecision
Mechanical dropper: Remifentanil	0	Some concerns	Low risk	Major concerns	Major concerns	No concerns	No concerns	low	Indirectness, Imprecision
Mechanical dropper: Salbutamol	0	Some concerns	Low risk	Major concerns	Major concerns	No concerns	No concerns	low	Indirectness, Imprecision
Mechanical dropper: Sufentani;	0	Some concerns	Low risk	Major concerns	Major concerns	No concerns	No concerns	low	Indirectness, Imprecision
Mechanical dropper: Tramadol	0	Some concerns	Low risk	Major concerns	Major concerns	No concerns	No concerns	low	Indirectness, Imprecision
MgSO4: Nalbuphine	0	No concerns	Low risk	Major concerns	Major concerns	No concerns	No concerns	low	Indirectness, Imprecision
MgSO4: Nalmefene	0	No concerns	Low risk	Major concerns	Major concerns	No concerns	No concerns	low	Indirectness, Imprecision
MgSO4: Naloxone	0	Some concerns	Low risk	Major concerns	Major concerns	No concerns	No concerns	low	Indirectness, Imprecision
MgSO4: Oxycodone	0	Some concerns	Low risk	Major concerns	Major concerns	No concerns	No concerns	low	Indirectness, Imprecision

MgSO4: Remifentanil	0	Some concerns	Low risk	Major concerns	Major concerns	No concerns	No concerns	low	Indirectness, Imprecision
MgSO4: Salbutamol	0	Some concerns	Low risk	Major concerns	Major concerns	No concerns	No concerns	low	Indirectness, Imprecision
MgSO4: Sufentani;	0	Some concerns	Low risk	Major concerns	Major concerns	No concerns	No concerns	low	Indirectness, Imprecision
MgSO4: Tramadol	0	Some concerns	Low risk	Major concerns	Major concerns	No concerns	No concerns	low	Indirectness, Imprecision
Nalbuphine: Nalmefene	0	No concerns	Low risk	Major concerns	Major concerns	No concerns	No concerns	low	Indirectness, Imprecision
Nalbuphine: Naloxone	0	Some concerns	Low risk	Major concerns	Major concerns	No concerns	No concerns	low	Indirectness, Imprecision
Nalbuphine: Oxycodone	0	Some concerns	Low risk	Major concerns	Major concerns	No concerns	No concerns	low	Indirectness, Imprecision
Nalbuphine: Remifentanil	0	Some concerns	Low risk	Major concerns	Major concerns	No concerns	No concerns	low	Indirectness, Imprecision
Nalbuphine: Salbutamol	0	Some concerns	Low risk	Major concerns	Major concerns	No concerns	No concerns	low	Indirectness, Imprecision

Nalbuphine: Sufentani;	0	Some concerns	Low risk	Major concerns	Major concerns	No concerns	No concerns	low	Indirectness, Imprecision
Nalbuphine: Tramadol	0	Some concerns	Low risk	Major concerns	Some concerns	Some concerns	No concerns	low	Indirectness, Imprecision, Heterogeneity
Nalmefene: Naloxone	0	Some concerns	Low risk	Major concerns	Major concerns	No concerns	No concerns	low	Indirectness, Imprecision
Nalmefene: Oxycodone	0	Some concerns	Low risk	Major concerns	Major concerns	No concerns	No concerns	low	Indirectness, Imprecision
Nalmefene: Remifentanil	0	Some concerns	Low risk	Major concerns	Major concerns	No concerns	No concerns	low	Indirectness, Imprecision
Nalmefene: Salbutamol	0	Some concerns	Low risk	Major concerns	Major concerns	No concerns	No concerns	low	Indirectness, Imprecision
Nalmefene: Sufentani;	0	Some concerns	Low risk	Major concerns	Major concerns	No concerns	No concerns	low	Indirectness, Imprecision
Nalmefene: Tramadol	0	Some concerns	Low risk	Major concerns	Major concerns	No concerns	No concerns	low	Indirectness, Imprecision
Naloxone: Oxycodone	0	Some concerns	Low risk	Major concerns	Major concerns	No concerns	No concerns	low	Indirectness, Imprecision

Naloxone: Remifentanil	0	Some concerns	Low risk	Major concerns	Major concerns	No concerns	No concerns	low	Indirectness, Imprecision
Naloxone: Salbutamol	0	Some concerns	Low risk	Major concerns	Major concerns	No concerns	No concerns	low	Indirectness, Imprecision
Naloxone: Sufentani;	0	Some concerns	Low risk	Major concerns	Major concerns	No concerns	No concerns	low	Indirectness, Imprecision
Naloxone: Tramadol	0	Some concerns	Low risk	Major concerns	Major concerns	No concerns	No concerns	low	Indirectness, Imprecision
Oxycodone: Remifentanil	0	Some concerns	Low risk	Major concerns	Major concerns	No concerns	No concerns	low	Indirectness, Imprecision
Oxycodone: Salbutamol	0	Some concerns	Low risk	Major concerns	Major concerns	No concerns	No concerns	low	Indirectness, Imprecision
Oxycodone: Sufentani;	0	Some concerns	Low risk	Major concerns	Major concerns	No concerns	No concerns	low	Indirectness, Imprecision
Oxycodone: Tramadol	0	Some concerns	Low risk	Major concerns	Major concerns	No concerns	No concerns	low	Indirectness, Imprecision
Remifentanil: Salbutamol	0	Some concerns	Low risk	Major concerns	Major concerns	No concerns	No concerns	low	Indirectness, Imprecision

Remifentanil:<br><br>Sufentani;	0	Some concerns	Low risk	Major concerns	Major concerns	No concerns	No concerns	low	Indirectness, Imprecision
Remifentanil:<br><br>Tramadol	0	Some concerns	Low risk	Major concerns	Major concerns	No concerns	No concerns	low	Indirectness, Imprecision
Salbutamol:<br><br>Sufentani;	0	Some concerns	Low risk	Major concerns	Major concerns	No concerns	No concerns	low	Indirectness, Imprecision
Salbutamol:<br><br>Tramadol	0	Some concerns	Low risk	Major concerns	Major concerns	No concerns	No concerns	low	Indirectness, Imprecision
Sufentani;:<br><br>Tramadol	0	Some concerns	Low risk	Major concerns	Major concerns	No concerns	No concerns	low	Indirectness, Imprecision

Table S4 B. Summary of findings table and GRADE assessment of NMA for the incidence of mild sufentanil-induced cough.

Comparison	Number of studies	Within-study Bias [1]	Reporting bias	Indirectness	Imprecision [2]	Heterogeneity [3]	Incoherence [4]	Confidence rating	Reason(s) for downgrading
Alfentanil:<br><br>Normal saline	1	No concerns	Low risk	No concerns	Some concerns	Some concerns	No concerns	low	Imprecision, Heterogeneity
Butorphanol:<br><br>Normal saline	3	Some concerns	Low risk	No concerns	Major concerns	No concerns	No concerns	moderate	Imprecision
Dexamethasone:<br><br>Dexmedetomidine	1	Some concerns	Low risk	No concerns	Major concerns	No concerns	No concerns	moderate	Imprecision
Dexamethasone:<br><br>Normal saline	3	Some concerns	Low risk	No concerns	No concerns	Some concerns	No concerns	moderate	Heterogeneity
Dexamethasone:<br><br>Salbutamol	1	Some concerns	Low risk	No concerns	Major concerns	No concerns	No concerns	moderate	Imprecision
Dexmedetomidine:<br><br>Normal saline	2	Some concerns	Low risk	No concerns	No concerns	Some concerns	No concerns	moderate	Heterogeneity
Dezocine:<br><br>Normal saline	8	Some concerns	Low risk	No concerns	No concerns	No concerns	No concerns	High	-
Dezocine:	1	Some concerns	Low risk	No concerns	No concerns	No concerns	No concerns	High	-

Oxycodone									
Esketamine:<br><br>Normal saline	1	No concerns	Low risk	No concerns	Some concerns	Some concerns	No concerns	low	Imprecision, Heterogeneity
Ketorolac tromethamine:<br><br>Normal saline	1	Some concerns	Low risk	No concerns	Major concerns	No concerns	No concerns	moderate	Imprecision
Lidocaine:<br><br>Normal saline	1	Some concerns	Low risk	No concerns	Major concerns	No concerns	No concerns	moderate	Imprecision
Mechanical dropper:<br><br>Normal saline	1	Some concerns	Low risk	No concerns	Major concerns	No concerns	No concerns	moderate	Imprecision
MgSO4:<br><br>Normal saline	1	No concerns	Low risk	No concerns	No concerns	No concerns	No concerns	High	-
Nalbuphine:<br><br>Normal saline	3	Some concerns	Low risk	No concerns	No concerns	No concerns	No concerns	High	-
Nalmefene:<br><br>Normal saline	2	No concerns	Low risk	No concerns	Some concerns	Some concerns	No concerns	low	Imprecision, Heterogeneity
Naloxone:<br><br>Normal saline	1	Some concerns	Low risk	No concerns	Major concerns	No concerns	No concerns	moderate	Imprecision

Normal saline: Oxycodone	1	Some concerns	Low risk	No concerns	Major concerns	No concerns	No concerns	moderate	Imprecision
Normal saline: Remifentanil	2	Some concerns	Low risk	No concerns	Major concerns	No concerns	No concerns	moderate	Imprecision
Normal saline: Salbutamol	1	Some concerns	Low risk	No concerns	Major concerns	No concerns	No concerns	moderate	Imprecision
Normal saline: Sufentanil	2	Some concerns	Low risk	No concerns	Some concerns	Some concerns	No concerns	low	Imprecision, Heterogeneity
Normal saline: Tramadol	1	Some concerns	Low risk	No concerns	Major concerns	No concerns	No concerns	moderate	Imprecision
Alfentanil: Butorphanol	0	Some concerns	Low risk	Major concerns	Major concerns	No concerns	No concerns	low	Indirectness, Imprecision
Alfentanil: Dexamethasone	0	Some concerns	Low risk	Major concerns	Major concerns	No concerns	No concerns	low	Indirectness, Imprecision
Alfentanil: Dexmedetomidine	0	Some concerns	Low risk	Major concerns	Major concerns	No concerns	No concerns	low	Indirectness, Imprecision
Alfentanil: Dezocine	0	No concerns	Low risk	Major concerns	Major concerns	No concerns	No concerns	low	Indirectness, Imprecision

Alfentanil:<br><br>Esketamine	0	No concerns	Low risk	Major concerns	Major concerns	No concerns	No concerns	low	Indirectness, Imprecision
Alfentanil:<br><br>Ketorolac tromethamine	0	Some concerns	Low risk	Major concerns	Major concerns	No concerns	No concerns	low	Indirectness, Imprecision
Alfentanil:<br><br>Lidocaine	0	Some concerns	Low risk	Major concerns	Major concerns	No concerns	No concerns	low	Indirectness, Imprecision
Alfentanil:<br><br>Mechanical dropper	0	Some concerns	Low risk	Major concerns	Major concerns	No concerns	No concerns	low	Indirectness, Imprecision
Alfentanil:<br><br>MgSO4	0	No concerns	Low risk	Major concerns	Major concerns	No concerns	No concerns	low	Indirectness, Imprecision
Alfentanil:<br><br>Nalbuphine	0	No concerns	Low risk	Major concerns	Major concerns	No concerns	No concerns	low	Indirectness, Imprecision
Alfentanil:<br><br>Nalmefene	0	No concerns	Low risk	Major concerns	Major concerns	No concerns	No concerns	low	Indirectness, Imprecision
Alfentanil:<br><br>Naloxone	0	Some concerns	Low risk	Major concerns	Major concerns	No concerns	No concerns	low	Indirectness, Imprecision
Alfentanil:	0	Some concerns	Low risk	Major concerns	Some concerns	Some concerns	No concerns	very low	Indirectness, Imprecision,

Oxycodone									Heterogeneity
Alfentanil:<br><br>Remifentanil	0	Some concerns	Low risk	Major concerns	Major concerns	No concerns	No concerns	low	Indirectness, Imprecision
Alfentanil:<br><br>Salbutamol	0	Some concerns	Low risk	Major concerns	Major concerns	No concerns	No concerns	low	Indirectness, Imprecision
Alfentanil:<br><br>Sufentanil	0	Some concerns	Low risk	Major concerns	Major concerns	No concerns	No concerns	low	Indirectness, Imprecision
Alfentanil:<br><br>Tramadol	0	Some concerns	Low risk	Major concerns	Major concerns	No concerns	No concerns	low	Indirectness, Imprecision
Butorphanol:<br><br>Dexamethasone	0	Some concerns	Low risk	Major concerns	Major concerns	No concerns	No concerns	low	Indirectness, Imprecision
Butorphanol:<br><br>Dexmedetomidine	0	Some concerns	Low risk	Major concerns	Major concerns	No concerns	No concerns	low	Indirectness, Imprecision
Butorphanol:<br><br>Dezocine	0	Some concerns	Low risk	Major concerns	Major concerns	No concerns	No concerns	low	Indirectness, Imprecision
Butorphanol:<br><br>Esketamine	0	Some concerns	Low risk	Major concerns	Major concerns	No concerns	No concerns	low	Indirectness, Imprecision

Butorphanol:<br><br>Ketorolac tromethamine	0	Some concerns	Low risk	Major concerns	Major concerns	No concerns	No concerns	low	Indirectness, Imprecision
Butorphanol:<br><br>Lidocaine	0	Some concerns	Low risk	Major concerns	Major concerns	No concerns	No concerns	low	Indirectness, Imprecision
Butorphanol:<br><br>Mechanical dropper	0	Some concerns	Low risk	Major concerns	Major concerns	No concerns	No concerns	low	Indirectness, Imprecision
Butorphanol:<br><br>MgSO4	0	Some concerns	Low risk	Major concerns	Major concerns	No concerns	No concerns	low	Indirectness, Imprecision
Butorphanol:<br><br>Nalbuphine	0	Some concerns	Low risk	Major concerns	Major concerns	No concerns	No concerns	low	Indirectness, Imprecision
Butorphanol:<br><br>Nalmefene	0	Some concerns	Low risk	Major concerns	Major concerns	No concerns	No concerns	low	Indirectness, Imprecision
Butorphanol:<br><br>Naloxone	0	Some concerns	Low risk	Major concerns	Major concerns	No concerns	No concerns	low	Indirectness, Imprecision
Butorphanol:<br><br>Oxycodone	0	Some concerns	Low risk	Major concerns	Major concerns	No concerns	No concerns	low	Indirectness, Imprecision
Butorphanol:	0	Some concerns	Low risk	Major concerns	Major concerns	No concerns	No concerns	low	Indirectness, Imprecision

Remifentanil									
Butorphanol:<br><br>Salbutamol	0	Some concerns	Low risk	Major concerns	Major concerns	No concerns	No concerns	low	Indirectness, Imprecision
Butorphanol:<br><br>Sufentanil	0	Some concerns	Low risk	Major concerns	Major concerns	No concerns	No concerns	low	Indirectness, Imprecision
Butorphanol:<br><br>Tramadol	0	Some concerns	Low risk	Major concerns	Major concerns	No concerns	No concerns	low	Indirectness, Imprecision
Dexamethasone:<br><br>Dezocine	0	Some concerns	Low risk	Major concerns	No concerns	Major concerns	No concerns	low	Indirectness, Heterogeneity
Dexamethasone:<br><br>Esketamine	0	Some concerns	Low risk	Major concerns	Major concerns	No concerns	No concerns	low	Indirectness, Imprecision
Dexamethasone:<br><br>Ketorolac tromethamine	0	Some concerns	Low risk	Major concerns	Major concerns	No concerns	No concerns	low	Indirectness, Imprecision
Dexamethasone:<br><br>Lidocaine	0	Some concerns	Low risk	Major concerns	Major concerns	No concerns	No concerns	low	Indirectness, Imprecision
Dexamethasone:<br><br>Mechanical dropper	0	Some concerns	Low risk	Major concerns	Major concerns	No concerns	No concerns	low	Indirectness, Imprecision

Dexamethasone: MgSO4	0	Some concerns	Low risk	Major concerns	Major concerns	No concerns	No concerns	low	Indirectness, Imprecision
Dexamethasone: Nalbuphine	0	Some concerns	Low risk	Major concerns	Some concerns	Some concerns	No concerns	very low	Indirectness, Imprecision, Heterogeneity
Dexamethasone: Nalmefene	0	Some concerns	Low risk	Major concerns	Major concerns	No concerns	No concerns	low	Indirectness, Imprecision
Dexamethasone: Naloxone	0	Some concerns	Low risk	Major concerns	Major concerns	No concerns	No concerns	low	Indirectness, Imprecision
Dexamethasone: Oxycodone	0	Some concerns	Low risk	Major concerns	Major concerns	No concerns	No concerns	low	Indirectness, Imprecision
Dexamethasone: Remifentanil	0	Some concerns	Low risk	Major concerns	Major concerns	No concerns	No concerns	low	Indirectness, Imprecision
Dexamethasone: Sufentanil	0	Some concerns	Low risk	Major concerns	Major concerns	No concerns	No concerns	low	Indirectness, Imprecision
Dexamethasone: Tramadol	0	Some concerns	Low risk	Major concerns	Major concerns	No concerns	No concerns	low	Indirectness, Imprecision
Dexmedetomidine: Dezocine	0	Some concerns	Low risk	Major concerns	Major concerns	No concerns	No concerns	low	Indirectness, Imprecision

Dexmedetomidine:<br><br>Esketamine	0	Some concerns	Low risk	Major concerns	Major concerns	No concerns	No concerns	low	Indirectness, Imprecision
Dexmedetomidine:<br><br>Ketorolac tromethamine	0	Some concerns	Low risk	Major concerns	Major concerns	No concerns	No concerns	low	Indirectness, Imprecision
Dexmedetomidine:<br><br>Lidocaine	0	Some concerns	Low risk	Major concerns	Major concerns	No concerns	No concerns	low	Indirectness, Imprecision
Dexmedetomidine:<br><br>Mechanical dropper	0	Some concerns	Low risk	Major concerns	Major concerns	No concerns	No concerns	low	Indirectness, Imprecision
Dexmedetomidine:<br><br>MgSO4	0	Some concerns	Low risk	Major concerns	Major concerns	No concerns	No concerns	low	Indirectness, Imprecision
Dexmedetomidine:<br><br>Nalbuphine	0	Some concerns	Low risk	Major concerns	Major concerns	No concerns	No concerns	low	Indirectness, Imprecision
Dexmedetomidine:<br><br>Nalmefene	0	Some concerns	Low risk	Major concerns	Major concerns	No concerns	No concerns	low	Indirectness, Imprecision
Dexmedetomidine:<br><br>Naloxone	0	Some concerns	Low risk	Major concerns	Major concerns	No concerns	No concerns	low	Indirectness, Imprecision
Dexmedetomidine:	0	Some concerns	Low risk	Major concerns	Some concerns	Some concerns	No concerns	very low	Indirectness, Imprecision,

Oxycodone									Heterogeneity
Dexmedetomidine:<br><br>Remifentanil	0	Some concerns	Low risk	Major concerns	Major concerns	No concerns	No concerns	low	Indirectness, Imprecision
Dexmedetomidine:<br><br>Salbutamol	0	Some concerns	Low risk	Major concerns	Major concerns	No concerns	No concerns	low	Indirectness, Imprecision
Dexmedetomidine:<br><br>Sufentanil	0	Some concerns	Low risk	Major concerns	Major concerns	No concerns	No concerns	low	Indirectness, Imprecision
Dexmedetomidine:<br><br>Tramadol	0	Some concerns	Low risk	Major concerns	Major concerns	No concerns	No concerns	low	Indirectness, Imprecision
Dezocine:<br><br>Esketamine	0	No concerns	Low risk	Major concerns	Major concerns	No concerns	No concerns	low	Indirectness, Imprecision
Dezocine:<br><br>Ketorolac tromethamine	0	Some concerns	Low risk	Major concerns	Major concerns	No concerns	No concerns	low	Indirectness, Imprecision
Dezocine:<br><br>Lidocaine	0	Some concerns	Low risk	Major concerns	Major concerns	No concerns	No concerns	low	Indirectness, Imprecision
Dezocine:<br><br>Mechanical dropper	0	Some concerns	Low risk	Major concerns	Major concerns	No concerns	No concerns	low	Indirectness, Imprecision

Dezocine: MgSO4	0	No concerns	Low risk	Major concerns	Major concerns	No concerns	No concerns	low	Indirectness, Imprecision
Dezocine: Nalbuphine	0	Some concerns	Low risk	Major concerns	Major concerns	No concerns	No concerns	low	Indirectness, Imprecision
Dezocine: Nalmefene	0	Some concerns	Low risk	Major concerns	Major concerns	No concerns	No concerns	low	Indirectness, Imprecision
Dezocine: Naloxone	0	Some concerns	Low risk	Major concerns	No concerns	Some concerns	No concerns	low	Indirectness, Heterogeneity
Dezocine: Remifentanil	0	Some concerns	Low risk	Major concerns	Major concerns	No concerns	No concerns	low	Indirectness, Imprecision
Dezocine: Salbutamol	0	Some concerns	Low risk	Major concerns	Major concerns	No concerns	No concerns	low	Indirectness, Imprecision
Dezocine: Sufentanil	0	Some concerns	Low risk	Major concerns	Major concerns	No concerns	No concerns	low	Indirectness, Imprecision
Dezocine: Tramadol	0	Some concerns	Low risk	Major concerns	Major concerns	No concerns	No concerns	low	Indirectness, Imprecision
Esketamine: Ketorolac	0	Some concerns	Low risk	Major concerns	Major concerns	No concerns	No concerns	low	Indirectness, Imprecision

tromethamine									
Esketamine:<br><br>Lidocaine	0	Some concerns	Low risk	Major concerns	Major concerns	No concerns	No concerns	low	Indirectness, Imprecision
Esketamine:<br><br>Mechanical dropper	0	Some concerns	Low risk	Major concerns	Major concerns	No concerns	No concerns	low	Indirectness, Imprecision
Esketamine:<br><br>MgSO4	0	No concerns	Low risk	Major concerns	Major concerns	No concerns	No concerns	low	Indirectness, Imprecision
Esketamine:<br><br>Nalbuphine	0	No concerns	Low risk	Major concerns	Major concerns	No concerns	No concerns	low	Indirectness, Imprecision
Esketamine:<br><br>Nalmefene	0	No concerns	Low risk	Major concerns	Major concerns	No concerns	No concerns	low	Indirectness, Imprecision
Esketamine:<br><br>Naloxone	0	Some concerns	Low risk	Major concerns	Major concerns	No concerns	No concerns	low	Indirectness, Imprecision
Esketamine:<br><br>Oxycodone	0	Some concerns	Low risk	Major concerns	Some concerns	Some concerns	No concerns	very low	Indirectness, Imprecision, Heterogeneity
Esketamine:<br><br>Remifentanil	0	Some concerns	Low risk	Major concerns	Major concerns	No concerns	No concerns	low	Indirectness, Imprecision

Esketamine:<br><br>Salbutamol	0	Some concerns	Low risk	Major concerns	Major concerns	No concerns	No concerns	low	Indirectness, Imprecision
Esketamine:<br><br>Sufentanil	0	Some concerns	Low risk	Major concerns	Major concerns	No concerns	No concerns	low	Indirectness, Imprecision
Esketamine:<br><br>Tramadol	0	Some concerns	Low risk	Major concerns	Major concerns	No concerns	No concerns	low	Indirectness, Imprecision
Ketorolac tromethamine:<br><br>Lidocaine	0	Some concerns	Low risk	Major concerns	Major concerns	No concerns	No concerns	low	Indirectness, Imprecision
Ketorolac tromethamine:<br><br>Mechanical dropper	0	Some concerns	Low risk	Major concerns	Major concerns	No concerns	No concerns	low	Indirectness, Imprecision
Ketorolac tromethamine:<br><br>MgSO4	0	Some concerns	Low risk	Major concerns	Major concerns	No concerns	No concerns	low	Indirectness, Imprecision
Ketorolac tromethamine:<br><br>Nalbuphine	0	Some concerns	Low risk	Major concerns	Major concerns	No concerns	No concerns	low	Indirectness, Imprecision
Ketorolac tromethamine:	0	Some concerns	Low risk	Major concerns	Major concerns	No concerns	No concerns	low	Indirectness, Imprecision

Nalmefene									
Ketorolac tromethamine:<br><br>Naloxone	0	Some concerns	Low risk	Major concerns	Major concerns	No concerns	No concerns	low	Indirectness, Imprecision
Ketorolac tromethamine:<br><br>Oxycodone	0	Some concerns	Low risk	Major concerns	Major concerns	No concerns	No concerns	low	Indirectness, Imprecision
Ketorolac tromethamine:<br><br>Remifentanil	0	Some concerns	Low risk	Major concerns	Major concerns	No concerns	No concerns	low	Indirectness, Imprecision
Ketorolac tromethamine:<br><br>Salbutamol	0	Some concerns	Low risk	Major concerns	Major concerns	No concerns	No concerns	low	Indirectness, Imprecision
Ketorolac tromethamine:<br><br>Sufentanil	0	Some concerns	Low risk	Major concerns	Major concerns	No concerns	No concerns	low	Indirectness, Imprecision
Ketorolac tromethamine:<br><br>Tramadol	0	Some concerns	Low risk	Major concerns	Major concerns	No concerns	No concerns	low	Indirectness, Imprecision
Lidocaine:	0	Some concerns	Low risk	Major concerns	Major concerns	No concerns	No concerns	low	Indirectness, Imprecision

Mechanical dropper									
Lidocaine: MgSO4	0	Some concerns	Low risk	Major concerns	Major concerns	No concerns	No concerns	low	Indirectness, Imprecision
Lidocaine: Nalbuphine	0	Some concerns	Low risk	Major concerns	Major concerns	No concerns	No concerns	low	Indirectness, Imprecision
Lidocaine: Nalmefene	0	Some concerns	Low risk	Major concerns	Major concerns	No concerns	No concerns	low	Indirectness, Imprecision
Lidocaine: Naloxone	0	Some concerns	Low risk	Major concerns	Major concerns	No concerns	No concerns	low	Indirectness, Imprecision
Lidocaine: Oxycodone	0	Some concerns	Low risk	Major concerns	Major concerns	No concerns	No concerns	low	Indirectness, Imprecision
Lidocaine: Remifentanil	0	Some concerns	Low risk	Major concerns	Major concerns	No concerns	No concerns	low	Indirectness, Imprecision
Lidocaine: Salbutamol	0	Some concerns	Low risk	Major concerns	Major concerns	No concerns	No concerns	low	Indirectness, Imprecision
Lidocaine: Sufentanil	0	Some concerns	Low risk	Major concerns	Major concerns	No concerns	No concerns	low	Indirectness, Imprecision

Lidocaine: Tramadol	0	Some concerns	Low risk	Major concerns	Major concerns	No concerns	No concerns	low	Indirectness, Imprecision
Mechanical dropper: MgSO4	0	Some concerns	Low risk	Major concerns	Major concerns	No concerns	No concerns	low	Indirectness, Imprecision
Mechanical dropper: Nalbuphine	0	Some concerns	Low risk	Major concerns	Major concerns	No concerns	No concerns	low	Indirectness, Imprecision
Mechanical dropper: Nalmefene	0	Some concerns	Low risk	Major concerns	Major concerns	No concerns	No concerns	low	Indirectness, Imprecision
Mechanical dropper: Naloxone	0	Some concerns	Low risk	Major concerns	Major concerns	No concerns	No concerns	low	Indirectness, Imprecision
Mechanical dropper: Oxycodone	0	Some concerns	Low risk	Major concerns	Major concerns	No concerns	No concerns	low	Indirectness, Imprecision
Mechanical dropper: Remifentanil	0	Some concerns	Low risk	Major concerns	Major concerns	No concerns	No concerns	low	Indirectness, Imprecision
Mechanical dropper: Salbutamol	0	Some concerns	Low risk	Major concerns	Major concerns	No concerns	No concerns	low	Indirectness, Imprecision
Mechanical dropper: Sufentanil	0	Some concerns	Low risk	Major concerns	Major concerns	No concerns	No concerns	low	Indirectness, Imprecision

Mechanical dropper: Tramadol	0	Some concerns	Low risk	Major concerns	Major concerns	No concerns	No concerns	low	Indirectness, Imprecision
MgSO4: Nalbuphine	0	No concerns	Low risk	Major concerns	Major concerns	No concerns	No concerns	low	Indirectness, Imprecision
MgSO4: Nalmefene	0	No concerns	Low risk	Major concerns	Major concerns	No concerns	No concerns	low	Indirectness, Imprecision
MgSO4: Naloxone	0	Some concerns	Low risk	Major concerns	Major concerns	No concerns	No concerns	low	Indirectness, Imprecision
MgSO4: Oxycodone	0	Some concerns	Low risk	Major concerns	No concerns	No concerns	No concerns	moderate	Indirectness
MgSO4: Remifentanil	0	Some concerns	Low risk	Major concerns	Major concerns	No concerns	No concerns	low	Indirectness, Imprecision
MgSO4: Salbutamol	0	Some concerns	Low risk	Major concerns	Major concerns	No concerns	No concerns	low	Indirectness, Imprecision
MgSO4: Sufentanil	0	Some concerns	Low risk	Major concerns	Major concerns	No concerns	No concerns	low	Indirectness, Imprecision
MgSO4: Tramadol	0	Some concerns	Low risk	Major concerns	Major concerns	No concerns	No concerns	low	Indirectness, Imprecision

Nalbuphine: Nalmefene	0	Some concerns	Low risk	Major concerns	Major concerns	No concerns	No concerns	low	Indirectness, Imprecision
Nalbuphine: Naloxone	0	Some concerns	Low risk	Major concerns	No concerns	Some concerns	No concerns	low	Indirectness, Heterogeneity
Nalbuphine: Oxycodone	0	Some concerns	Low risk	Major concerns	No concerns	No concerns	No concerns	moderate	Indirectness
Nalbuphine: Remifentanil	0	Some concerns	Low risk	Major concerns	Major concerns	No concerns	No concerns	low	Indirectness, Imprecision
Nalbuphine: Salbutamol	0	Some concerns	Low risk	Major concerns	Major concerns	No concerns	No concerns	low	Indirectness, Imprecision
Nalbuphine: Sufentanil	0	Some concerns	Low risk	Major concerns	Major concerns	No concerns	No concerns	low	Indirectness, Imprecision
Nalbuphine: Tramadol	0	Some concerns	Low risk	Major concerns	Major concerns	No concerns	No concerns	low	Indirectness, Imprecision
Nalmefene: Naloxone	0	Some concerns	Low risk	Major concerns	Major concerns	No concerns	No concerns	low	Indirectness, Imprecision
Nalmefene: Oxycodone	0	Some concerns	Low risk	Major concerns	Major concerns	No concerns	No concerns	low	Indirectness, Imprecision

Nalmefene: Remifentanil	0	Some concerns	Low risk	Major concerns	Major concerns	No concerns	No concerns	low	Indirectness, Imprecision
Nalmefene: Salbutamol	0	Some concerns	Low risk	Major concerns	Major concerns	No concerns	No concerns	low	Indirectness, Imprecision
Nalmefene: Sufentanil	0	Some concerns	Low risk	Major concerns	Major concerns	No concerns	No concerns	low	Indirectness, Imprecision
Nalmefene: Tramadol	0	Some concerns	Low risk	Major concerns	Major concerns	No concerns	No concerns	low	Indirectness, Imprecision
Naloxone: Oxycodone	0	Some concerns	Low risk	Major concerns	Major concerns	No concerns	No concerns	low	Indirectness, Imprecision
Naloxone: Remifentanil	0	Some concerns	Low risk	Major concerns	Major concerns	No concerns	No concerns	low	Indirectness, Imprecision
Naloxone: Salbutamol	0	Some concerns	Low risk	Major concerns	Major concerns	No concerns	No concerns	low	Indirectness, Imprecision
Naloxone: Sufentanil	0	Some concerns	Low risk	Major concerns	Major concerns	No concerns	No concerns	low	Indirectness, Imprecision
Naloxone: Tramadol	0	Some concerns	Low risk	Major concerns	Major concerns	No concerns	No concerns	low	Indirectness, Imprecision

Oxycodone:<br><br>Remifentanil	0	Some concerns	Low risk	Major concerns	Major concerns	No concerns	No concerns	low	Indirectness, Imprecision
Oxycodone:<br><br>Salbutamol	0	Some concerns	Low risk	Major concerns	Major concerns	No concerns	No concerns	low	Indirectness, Imprecision
Oxycodone:<br><br>Sufentanil	0	Some concerns	Low risk	Major concerns	Some concerns	Some concerns	No concerns	very low	Indirectness, Imprecision, Heterogeneity
Oxycodone:<br><br>Tramadol	0	Some concerns	Low risk	Major concerns	Major concerns	No concerns	No concerns	low	Indirectness, Imprecision
Remifentanil:<br><br>Salbutamol	0	Some concerns	Low risk	Major concerns	Major concerns	No concerns	No concerns	low	Indirectness, Imprecision
Remifentanil:<br><br>Sufentanil	0	Some concerns	Low risk	Major concerns	Major concerns	No concerns	No concerns	low	Indirectness, Imprecision
Remifentanil:<br><br>Tramadol	0	Some concerns	Low risk	Major concerns	Major concerns	No concerns	No concerns	low	Indirectness, Imprecision
Salbutamol:<br><br>Sufentanil	0	Some concerns	Low risk	Major concerns	Major concerns	No concerns	No concerns	low	Indirectness, Imprecision
Salbutamol:<br><br>Tramadol	0	Some concerns	Low risk	Major concerns	Major concerns	No concerns	No concerns	low	Indirectness, Imprecision

Sufentanil:<br><br>Tramadol	0	Some concerns	Low risk	Major concerns	Major concerns	No concerns	No concerns	Low	Indirectness, Imprecision

Table S4 C. Summary of findings table and GRADE assessment of NMA for the incidence of moderate to severe sufentanil-induced cough.

Comparison	Number of studies	Within-study Bias [1]	Reporting bias	Indirectness	Imprecision [2]	Heterogeneity [3]	Incoherence [4]	Confidence rating	Reason(s) for downgrading
Alfentanil:<br>Normal saline	1	No concerns	Low risk	No concerns	No concerns	No concerns	No concerns	High	-
Butorphanol:<br><br>Normal saline	3	Some concerns	Low risk	No concerns	No concerns	No concerns	No concerns	High	-
Dexamethasone:<br><br>Dexmedetomidine	1	Some concerns	Low risk	No concerns	Major concerns	No concerns	No concerns	moderate	Imprecision
Dexamethasone:<br><br>Normal saline	3	Some concerns	Low risk	No concerns	No concerns	Some concerns	No concerns	moderate	Heterogeneity
Dexamethasone:<br><br>Salbutamol	1	Some concerns	Low risk	No concerns	Major concerns	No concerns	No concerns	moderate	Imprecision
Dexmedetomidine:<br><br>Normal saline	2	Some concerns	Low risk	No concerns	No concerns	No concerns	No concerns	High	-
Dezocine:<br><br>Normal saline	8	Some concerns	Low risk	No concerns	No concerns	No concerns	No concerns	High	-
Dezocine:	1	Some concerns	Low risk	No concerns	Major concerns	No concerns	No concerns	moderate	Imprecision

Oxycodone									
Esketamine:<br><br>Normal saline	1	No concerns	Low risk	No concerns	No concerns	No concerns	No concerns	High	-
Ketorolac tromethamine:<br><br>Normal saline	1	Some concerns	Low risk	No concerns	No concerns	No concerns	No concerns	High	-
Lidocaine:<br><br>Normal saline	1	Some concerns	Low risk	No concerns	Some concerns	Some concerns	No concerns	low	Imprecision, Heterogeneity
Mechanical dropper:<br><br>Normal saline	1	Some concerns	Low risk	No concerns	No concerns	No concerns	No concerns	High	-
MgSO4:<br><br>Normal saline	1	No concerns	Low risk	No concerns	No concerns	No concerns	No concerns	High	-
Nalbuphine:<br><br>Normal saline	3	Some concerns	Low risk	No concerns	No concerns	No concerns	No concerns	High	-
Nalmefene:<br><br>Normal saline	2	No concerns	Low risk	No concerns	No concerns	No concerns	No concerns	High	-
Naloxone:<br><br>Normal saline	1	Some concerns	Low risk	No concerns	No concerns	No concerns	No concerns	High	-

Normal saline: Oxycodone	1	Some concerns	Low risk	No concerns	No concerns	No concerns	No concerns	High	-
Normal saline: Remifentanil	2	Some concerns	Low risk	No concerns	No concerns	No concerns	No concerns	High	-
Normal saline: Salbutamol	1	Some concerns	Low risk	No concerns	Major concerns	No concerns	No concerns	moderate	Imprecision
Normal saline: Sufentanil	2	Some concerns	Low risk	No concerns	No concerns	No concerns	No concerns	High	-
Normal saline: Tramadol	1	Some concerns	Low risk	No concerns	Some concerns	No concerns	No concerns	moderate	Imprecision
Alfentanil: Butorphanol	0	Some concerns	Low risk	Major concerns	Major concerns	No concerns	No concerns	low	Indirectness, Imprecision
Alfentanil: Dexamethasone	0	Some concerns	Low risk	Major concerns	Major concerns	No concerns	No concerns	low	Indirectness, Imprecision
Alfentanil: Dexmedetomidine	0	Some concerns	Low risk	Major concerns	Major concerns	No concerns	No concerns	low	Indirectness, Imprecision
Alfentanil: Dezocine	0	No concerns	Low risk	Major concerns	Major concerns	No concerns	No concerns	low	Indirectness, Imprecision

Alfentanil:<br><br>Esketamine	0	No concerns	Low risk	Major concerns	Major concerns	No concerns	No concerns	low	Indirectness, Imprecision
Alfentanil:<br><br>Ketorolac tromethamine	0	Some concerns	Low risk	Major concerns	Major concerns	No concerns	No concerns	low	Indirectness, Imprecision
Alfentanil:<br><br>Lidocaine	0	Some concerns	Low risk	Major concerns	Major concerns	No concerns	No concerns	low	Indirectness, Imprecision
Alfentanil:<br><br>Mechanical dropper	0	Some concerns	Low risk	Major concerns	Major concerns	No concerns	No concerns	low	Indirectness, Imprecision
Alfentanil:<br><br>MgSO4	0	No concerns	Low risk	Major concerns	Major concerns	No concerns	No concerns	low	Indirectness, Imprecision
Alfentanil:<br><br>Nalbuphine	0	No concerns	Low risk	Major concerns	Major concerns	No concerns	No concerns	low	Indirectness, Imprecision
Alfentanil:<br><br>Nalmefene	0	No concerns	Low risk	Major concerns	Major concerns	No concerns	No concerns	low	Indirectness, Imprecision
Alfentanil:<br><br>Naloxone	0	Some concerns	Low risk	Major concerns	Major concerns	No concerns	No concerns	low	Indirectness, Imprecision
Alfentanil:	0	Some concerns	Low risk	Major concerns	Major concerns	No concerns	No concerns	low	Indirectness, Imprecision

Oxycodone									
Alfentanil: Remifentanil	0	Some concerns	Low risk	Major concerns	Major concerns	No concerns	No concerns	low	Indirectness, Imprecision
Alfentanil: Salbutamol	0	Some concerns	Low risk	Major concerns	Major concerns	No concerns	No concerns	low	Indirectness, Imprecision
Alfentanil: Sufentanil	0	Some concerns	Low risk	Major concerns	Major concerns	No concerns	No concerns	low	Indirectness, Imprecision
Alfentanil: Tramadol	0	Some concerns	Low risk	Major concerns	Major concerns	No concerns	No concerns	low	Indirectness, Imprecision
Butorphanol: Dexamethasone	0	Some concerns	Low risk	Major concerns	No concerns	No concerns	No concerns	moderate	Indirectness
Butorphanol: Dexmedetomidine	0	Some concerns	Low risk	Major concerns	No concerns	No concerns	No concerns	moderate	Indirectness
Butorphanol: Dezocine	0	Some concerns	Low risk	Major concerns	No concerns	No concerns	No concerns	moderate	Indirectness
Butorphanol: Esketamine	0	Some concerns	Low risk	Major concerns	Some concerns	Some concerns	No concerns	very low	Indirectness, Imprecision, Heterogeneity

Butorphanol:<br><br>Ketorolac tromethamine	0	Some concerns	Low risk	Major concerns	Major concerns	No concerns	No concerns	low	Indirectness, Imprecision
Butorphanol:<br><br>Lidocaine	0	Some concerns	Low risk	Major concerns	No concerns	No concerns	No concerns	moderate	Indirectness
Butorphanol:<br><br>Mechanical dropper	0	Some concerns	Low risk	Major concerns	Major concerns	No concerns	No concerns	low	Indirectness, Imprecision
Butorphanol:<br><br>MgSO4	0	Some concerns	Low risk	Major concerns	Major concerns	No concerns	No concerns	low	Indirectness, Imprecision
Butorphanol:<br><br>Nalbuphine	0	Some concerns	Low risk	Major concerns	Major concerns	No concerns	No concerns	low	Indirectness, Imprecision
Butorphanol:<br><br>Nalmefene	0	Some concerns	Low risk	Major concerns	No concerns	No concerns	No concerns	moderate	Indirectness
Butorphanol:<br><br>Naloxone	0	Some concerns	Low risk	Major concerns	No concerns	No concerns	No concerns	moderate	Indirectness
Butorphanol:<br><br>Oxycodone	0	Some concerns	Low risk	Major concerns	Major concerns	No concerns	No concerns	low	Indirectness, Imprecision
Butorphanol:	0	Some concerns	Low risk	Major concerns	Major concerns	No concerns	No concerns	low	Indirectness, Imprecision

Remifentanil									
Butorphanol: Salbutamol	0	Some concerns	Low risk	Major concerns	No concerns	No concerns	No concerns	moderate	Indirectness
Butorphanol: Sufentanil	0	Some concerns	Low risk	Major concerns	No concerns	No concerns	No concerns	moderate	Indirectness
Butorphanol: Tramadol	0	Some concerns	Low risk	Major concerns	No concerns	No concerns	No concerns	moderate	Indirectness
Dexamethasone: Dezocine	0	Some concerns	Low risk	Major concerns	No concerns	No concerns	No concerns	moderate	Indirectness
Dexamethasone: Esketamine	0	Some concerns	Low risk	Major concerns	No concerns	Some concerns	No concerns	low	Heterogeneity
Dexamethasone: Ketorolac tromethamine	0	Some concerns	Low risk	Major concerns	Major concerns	No concerns	No concerns	low	Indirectness, Imprecision
Dexamethasone: Lidocaine	0	Some concerns	Low risk	Major concerns	Major concerns	No concerns	No concerns	low	Indirectness, Imprecision
Dexamethasone: Mechanical dropper	0	Some concerns	Low risk	Major concerns	Major concerns	No concerns	No concerns	low	Indirectness, Imprecision

Dexamethasone: MgSO4	0	Some concerns	Low risk	Major concerns	No concerns	No concerns	No concerns	moderate	Indirectness
Dexamethasone: Nalbuphine	0	Some concerns	Low risk	Major concerns	Some concerns	Some concerns	No concerns	very low	Indirectness, Imprecision, Heterogeneity
Dexamethasone: Nalmefene	0	Some concerns	Low risk	Major concerns	Major concerns	No concerns	No concerns	low	Indirectness, Imprecision
Dexamethasone: Naloxone	0	Some concerns	Low risk	Major concerns	Major concerns	No concerns	No concerns	low	Indirectness, Imprecision
Dexamethasone: Oxycodone	0	Some concerns	Low risk	Major concerns	Some concerns	Some concerns	No concerns	very low	Indirectness, Imprecision, Heterogeneity
Dexamethasone: Remifentanil	0	Some concerns	Low risk	Major concerns	No concerns	No concerns	No concerns	low	Indirectness
Dexamethasone: Sufentanil	0	Some concerns	Low risk	Major concerns	Major concerns	No concerns	No concerns	moderate	Indirectness, Imprecision
Dexamethasone: Tramadol	0	Some concerns	Low risk	Major concerns	Major concerns	No concerns	No concerns	low	Indirectness, Imprecision
Dexmedetomidine: Dezocine	0	Some concerns	Low risk	Major concerns	Major concerns	No concerns	No concerns	low	Indirectness, Imprecision

Dexmedetomidine:<br><br>Esketamine	0	Some concerns	Low risk	Major concerns	Major concerns	No concerns	No concerns	low	Indirectness, Imprecision
Dexmedetomidine:<br><br>Ketorolac tromethamine	0	Some concerns	Low risk	Major concerns	Major concerns	No concerns	No concerns	low	Indirectness, Imprecision
Dexmedetomidine:<br><br>Lidocaine	0	Some concerns	Low risk	Major concerns	Major concerns	No concerns	No concerns	low	Indirectness, Imprecision
Dexmedetomidine:<br><br>Mechanical dropper	0	Some concerns	Low risk	Major concerns	Major concerns	No concerns	No concerns	low	Indirectness, Imprecision
Dexmedetomidine:<br><br>MgSO4	0	Some concerns	Low risk	Major concerns	Some concerns	Some concerns	No concerns	very low	Indirectness, Imprecision, Heterogeneity
Dexmedetomidine:<br><br>Nalbuphine	0	Some concerns	Low risk	Major concerns	Major concerns	No concerns	No concerns	low	Indirectness, Imprecision
Dexmedetomidine:<br><br>Nalmefene	0	Some concerns	Low risk	Major concerns	Major concerns	No concerns	No concerns	low	Indirectness, Imprecision
Dexmedetomidine:<br><br>Naloxone	0	Some concerns	Low risk	Major concerns	Major concerns	No concerns	No concerns	low	Indirectness, Imprecision
Dexmedetomidine:	0	Some concerns	Low risk	Major concerns	Major concerns	No concerns	No concerns	low	Indirectness, Imprecision

Oxycodone									
Dexmedetomidine: Remifentanil	0	Some concerns	Low risk	Major concerns	Major concerns	No concerns	No concerns	low	Indirectness, Imprecision
Dexmedetomidine: Salbutamol	0	Some concerns	Low risk	Major concerns	Major concerns	No concerns	No concerns	low	Indirectness, Imprecision
Dexmedetomidine: Sufentanil	0	Some concerns	Low risk	Major concerns	Major concerns	No concerns	No concerns	low	Indirectness, Imprecision
Dexmedetomidine: Tramadol	0	Some concerns	Low risk	Major concerns	Major concerns	No concerns	No concerns	low	Indirectness, Imprecision
Dezocine: Esketamine	0	No concerns	Low risk	Major concerns	Major concerns	No concerns	No concerns	low	Indirectness, Imprecision
Dezocine: Ketorolac tromethamine	0	Some concerns	Low risk	Major concerns	Major concerns	No concerns	No concerns	low	Indirectness, Imprecision
Dezocine: Lidocaine	0	Some concerns	Low risk	Major concerns	Major concerns	No concerns	No concerns	low	Indirectness, Imprecision
Dezocine: Mechanical dropper	0	Some concerns	Low risk	Major concerns	Major concerns	No concerns	No concerns	low	Indirectness, Imprecision

Dezocine:<br><br>MgSO4	0	No concerns	Low risk	Major concerns	Major concerns	No concerns	No concerns	low	Indirectness, Imprecision
Dezocine:<br><br>Nalbuphine	0	Some concerns	Low risk	Major concerns	Major concerns	No concerns	No concerns	low	Indirectness, Imprecision
Dezocine:<br><br>Nalmefene	0	Some concerns	Low risk	Major concerns	Major concerns	No concerns	No concerns	low	Indirectness, Imprecision
Dezocine:<br><br>Naloxone	0	Some concerns	Low risk	Major concerns	Major concerns	No concerns	No concerns	low	Indirectness, Imprecision
Dezocine:<br><br>Remifentanil	0	Some concerns	Low risk	Major concerns	Major concerns	No concerns	No concerns	low	Indirectness, Imprecision
Dezocine:<br><br>Salbutamol	0	Some concerns	Low risk	Major concerns	Major concerns	No concerns	No concerns	low	Indirectness, Imprecision
Dezocine:<br><br>Sufentanil	0	Some concerns	Low risk	Major concerns	Major concerns	No concerns	No concerns	low	Indirectness, Imprecision
Dezocine:<br><br>Tramadol	0	Some concerns	Low risk	Major concerns	Some concerns	Some concerns	No concerns	very low	Indirectness, Imprecision, Heterogeneity
Esketamine:<br><br>Ketorolac	0	Some concerns	Low risk	Major concerns	Major concerns	No concerns	No concerns	low	Indirectness, Imprecision

tromethamine									
Esketamine: Lidocaine	0	Some concerns	Low risk	Major concerns	Major concerns	No concerns	No concerns	low	Indirectness, Imprecision
Esketamine: Mechanical dropper	0	Some concerns	Low risk	Major concerns	Major concerns	No concerns	No concerns	low	Indirectness, Imprecision
Esketamine: MgSO4	0	No concerns	Low risk	Major concerns	Major concerns	No concerns	No concerns	low	Indirectness, Imprecision
Esketamine: Nalbuphine	0	No concerns	Low risk	Major concerns	Major concerns	No concerns	No concerns	low	Indirectness, Imprecision
Esketamine: Nalmefene	0	No concerns	Low risk	Major concerns	Major concerns	No concerns	No concerns	low	Indirectness, Imprecision
Esketamine: Naloxone	0	Some concerns	Low risk	Major concerns	Major concerns	No concerns	No concerns	low	Indirectness, Imprecision
Esketamine: Oxycodone	0	Some concerns	Low risk	Major concerns	Major concerns	No concerns	No concerns	low	Indirectness, Imprecision
Esketamine: Remifentanil	0	Some concerns	Low risk	Major concerns	Major concerns	No concerns	No concerns	low	Indirectness, Imprecision
Esketamine:	0	Some concerns	Low risk	Major concerns	Major concerns	No concerns	No concerns	low	Indirectness,

Salbutamol									Imprecision
Esketamine:<br><br>Sufentanil	0	Some concerns	Low risk	Major concerns	Major concerns	No concerns	No concerns	low	Indirectness, Imprecision
Esketamine:<br><br>Tramadol	0	Some concerns	Low risk	Major concerns	Major concerns	No concerns	No concerns	low	Indirectness, Imprecision
Ketorolac tromethamine:<br><br>Lidocaine	0	Some concerns	Low risk	Major concerns	Major concerns	No concerns	No concerns	low	Indirectness, Imprecision
Ketorolac tromethamine:<br><br>Mechanical dropper	0	Some concerns	Low risk	Major concerns	Major concerns	No concerns	No concerns	low	Indirectness, Imprecision
Ketorolac tromethamine:<br><br>MgSO4	0	Some concerns	Low risk	Major concerns	Major concerns	No concerns	No concerns	low	Indirectness, Imprecision
Ketorolac tromethamine:<br><br>Nalbuphine	0	Some concerns	Low risk	Major concerns	Major concerns	No concerns	No concerns	low	Indirectness, Imprecision
Ketorolac tromethamine:<br><br>Nalmefene	0	Some concerns	Low risk	Major concerns	Major concerns	No concerns	No concerns	low	Indirectness, Imprecision

Ketorolac tromethamine:<br><br>Naloxone	0	Some concerns	Low risk	Major concerns	Major concerns	No concerns	No concerns	low	Indirectness, Imprecision
Ketorolac tromethamine:<br><br>Oxycodone	0	Some concerns	Low risk	Major concerns	Major concerns	No concerns	No concerns	low	Indirectness, Imprecision
Ketorolac tromethamine:<br><br>Remifentanil	0	Some concerns	Low risk	Major concerns	Major concerns	No concerns	No concerns	low	Indirectness, Imprecision
Ketorolac tromethamine:<br><br>Salbutamol	0	Some concerns	Low risk	Major concerns	Major concerns	No concerns	No concerns	low	Indirectness, Imprecision
Ketorolac tromethamine:<br><br>Sufentanil	0	Some concerns	Low risk	Major concerns	Major concerns	No concerns	No concerns	low	Indirectness, Imprecision
Ketorolac tromethamine:<br><br>Tramadol	0	Some concerns	Low risk	Major concerns	Major concerns	No concerns	No concerns	low	Indirectness, Imprecision
Lidocaine:<br><br>Mechanical dropper	0	Some concerns	Low risk	Major concerns	Major concerns	No concerns	No concerns	low	Indirectness, Imprecision

Lidocaine: MgSO4	0	Some concerns	Low risk	Major concerns	Major concerns	No concerns	No concerns	low	Indirectness, Imprecision
Lidocaine: Nalbuphine	0	Some concerns	Low risk	Major concerns	Major concerns	No concerns	No concerns	low	Indirectness, Imprecision
Lidocaine: Nalmefene	0	Some concerns	Low risk	Major concerns	Major concerns	No concerns	No concerns	low	Indirectness, Imprecision
Lidocaine: Naloxone	0	Some concerns	Low risk	Major concerns	Major concerns	No concerns	No concerns	low	Indirectness, Imprecision
Lidocaine: Oxycodone	0	Some concerns	Low risk	Major concerns	Major concerns	No concerns	No concerns	low	Indirectness, Imprecision
Lidocaine: Remifentanil	0	Some concerns	Low risk	Major concerns	Major concerns	No concerns	No concerns	low	Indirectness, Imprecision
Lidocaine: Salbutamol	0	Some concerns	Low risk	Major concerns	Major concerns	No concerns	No concerns	low	Indirectness, Imprecision
Lidocaine: Sufentanil	0	Some concerns	Low risk	Major concerns	Major concerns	No concerns	No concerns	low	Indirectness, Imprecision
Lidocaine: Tramadol	0	Some concerns	Low risk	Major concerns	Major concerns	No concerns	No concerns	low	Indirectness, Imprecision

Mechanical dropper: MgSO4	0	Some concerns	Low risk	Major concerns	Major concerns	No concerns	No concerns	low	Indirectness, Imprecision
Mechanical dropper: Nalbuphine	0	Some concerns	Low risk	Major concerns	Major concerns	No concerns	No concerns	low	Indirectness, Imprecision
Mechanical dropper: Nalmefene	0	Some concerns	Low risk	Major concerns	Major concerns	No concerns	No concerns	low	Indirectness, Imprecision
Mechanical dropper: Naloxone	0	Some concerns	Low risk	Major concerns	Major concerns	No concerns	No concerns	low	Indirectness, Imprecision
Mechanical dropper: Oxycodone	0	Some concerns	Low risk	Major concerns	Major concerns	No concerns	No concerns	low	Indirectness, Imprecision
Mechanical dropper: Remifentanil	0	Some concerns	Low risk	Major concerns	Major concerns	No concerns	No concerns	low	Indirectness, Imprecision
Mechanical dropper: Salbutamol	0	Some concerns	Low risk	Major concerns	Major concerns	No concerns	No concerns	low	Indirectness, Imprecision
Mechanical dropper: Sufentanil	0	Some concerns	Low risk	Major concerns	Major concerns	No concerns	No concerns	low	Indirectness, Imprecision
Mechanical dropper: Tramadol	0	Some concerns	Low risk	Major concerns	Major concerns	No concerns	No concerns	low	Indirectness, Imprecision

MgSO4: Nalbuphine	0	No concerns	Low risk	Major concerns	Major concerns	No concerns	No concerns	low	Indirectness, Imprecision
MgSO4: Nalmefene	0	No concerns	Low risk	Major concerns	Major concerns	No concerns	No concerns	low	Indirectness, Imprecision
MgSO4: Naloxone	0	Some concerns	Low risk	Major concerns	Some concerns	Some concerns	No concerns	very low	Indirectness, Imprecision, Heterogeneity
MgSO4: Oxycodone	0	Some concerns	Low risk	Major concerns	Major concerns	No concerns	No concerns	low	Indirectness, Imprecision
MgSO4: Remifentanil	0	Some concerns	Low risk	Major concerns	Major concerns	No concerns	No concerns	low	Indirectness, Imprecision
MgSO4: Salbutamol	0	Some concerns	Low risk	Major concerns	Major concerns	No concerns	No concerns	low	Indirectness, Imprecision
MgSO4: Sufentanil	0	Some concerns	Low risk	Major concerns	Major concerns	No concerns	No concerns	low	Indirectness, Imprecision
MgSO4: Tramadol	0	Some concerns	Low risk	Major concerns	No concerns	No concerns	No concerns	moderate	Indirectness
Nalbuphine: Nalmefene	0	No concerns	Low risk	Major concerns	Major concerns	No concerns	No concerns	low	Indirectness, Imprecision

Nalbuphine:<br><br>Naloxone	0	Some concerns	Low risk	Major concerns	Major concerns	No concerns	No concerns	low	Indirectness, Imprecision
Nalbuphine:<br><br>Oxycodone	0	Some concerns	Low risk	Major concerns	Major concerns	No concerns	No concerns	low	Indirectness, Imprecision
Nalbuphine:<br><br>Remifentanil	0	Some concerns	Low risk	Major concerns	Major concerns	No concerns	No concerns	low	Indirectness, Imprecision
Nalbuphine:<br><br>Salbutamol	0	Some concerns	Low risk	Major concerns	Major concerns	No concerns	No concerns	low	Indirectness, Imprecision
Nalbuphine:<br><br>Sufentanil	0	Some concerns	Low risk	Major concerns	Major concerns	No concerns	No concerns	low	Indirectness, Imprecision
Nalbuphine:<br><br>Tramadol	0	Some concerns	Low risk	Major concerns	Major concerns	No concerns	No concerns	low	Indirectness, Imprecision
Nalmefene:<br><br>Naloxone	0	Some concerns	Low risk	Major concerns	Major concerns	No concerns	No concerns	low	Indirectness, Imprecision
Nalmefene:<br><br>Oxycodone	0	Some concerns	Low risk	Major concerns	Major concerns	No concerns	No concerns	low	Indirectness, Imprecision
Nalmefene:<br><br>Remifentanil	0	Some concerns	Low risk	Major concerns	Major concerns	No concerns	No concerns	low	Indirectness, Imprecision

Nalmefene:<br>Salbutamol	0	Some concerns	Low risk	Major concerns	Major concerns	No concerns	No concerns	low	Indirectness, Imprecision
Nalmefene:<br>Sufentanil	0	Some concerns	Low risk	Major concerns	Major concerns	No concerns	No concerns	low	Indirectness, Imprecision
Nalmefene:<br>Tramadol	0	Some concerns	Low risk	Major concerns	Major concerns	No concerns	No concerns	low	Indirectness, Imprecision
Naloxone:<br>Oxycodone	0	Some concerns	Low risk	Major concerns	Major concerns	No concerns	No concerns	low	Indirectness, Imprecision
Naloxone:<br>Remifentanil	0	Some concerns	Low risk	Major concerns	Major concerns	No concerns	No concerns	low	Indirectness, Imprecision
Naloxone:<br>Salbutamol	0	Some concerns	Low risk	Major concerns	Major concerns	No concerns	No concerns	low	Indirectness, Imprecision
Naloxone:<br>Sufentanil	0	Some concerns	Low risk	Major concerns	Major concerns	No concerns	No concerns	low	Indirectness, Imprecision
Naloxone:<br>Tramadol	0	Some concerns	Low risk	Major concerns	Major concerns	No concerns	No concerns	low	Indirectness, Imprecision
Oxycodone:<br>Remifentanil	0	Some concerns	Low risk	Major concerns	Major concerns	No concerns	No concerns	low	Indirectness, Imprecision

Oxycodone: Salbutamol	0	Some concerns	Low risk	Major concerns	Major concerns	No concerns	No concerns	low	Indirectness, Imprecision
Oxycodone: Sufentanil	0	Some concerns	Low risk	Major concerns	Major concerns	No concerns	No concerns	low	Indirectness, Imprecision
Oxycodone: Tramadol	0	Some concerns	Low risk	Major concerns	Major concerns	No concerns	No concerns	low	Indirectness, Imprecision
Remifentanil: Salbutamol	0	Some concerns	Low risk	Major concerns	Major concerns	No concerns	No concerns	low	Indirectness, Imprecision
Remifentanil: Sufentanil	0	Some concerns	Low risk	Major concerns	Major concerns	No concerns	No concerns	low	Indirectness, Imprecision
Remifentanil: Tramadol	0	Some concerns	Low risk	Major concerns	Some concerns	Some concerns	No concerns	very low	Indirectness, Imprecision, Heterogeneity
Salbutamol: Sufentanil	0	Some concerns	Low risk	Major concerns	Major concerns	No concerns	No concerns	low	Indirectness, Imprecision
Salbutamol: Tramadol	0	Some concerns	Low risk	Major concerns	Major concerns	No concerns	No concerns	low	Indirectness, Imprecision
Sufentanil: Tramadol	0	Some concerns	Low risk	Major concerns	Major concerns	No concerns	No concerns	low	Indirectness, Imprecision

Abbreviations for interventions are defined in the text.

High quality: Further research is very unlikely to change our confidence in the estimate of effect.

Moderate quality: Further research is likely to have an important impact on our confidence in the estimate of effect and may change the estimate.

Low quality: Further research is very likely to have an important impact on our confidence in the estimate of effect and is likely to change the estimate.

Very low quality: We are very uncertain about the estimate.

[1]Downgraded for serious risk of bias. We didn't downgrade the level of some concern.

[2] Downgraded all indirect evidence for serious inconsistency. Since the comparisons between different interventions are all indirect comparisons, the network meta-analysis does not have a closed loop and cannot perform the inconsistency model test.

[3]Downgraded for serious imprecision, because confidence interval extends into clinically important effects in both directions.

[4] Downgraded for serious heterogeneity, with prediction interval extends into clinically important effects in both directions.

Table S5 Result of heterogeneity test and meta-regression for conventional pair comparisons.

Outcomes/Covariate	No. of Studies	Test of heterogeneity		*P* value of Egger's test	Meta-regression	
		I-square	Tau-square		Median coefficient (95%CI)	*p* value|
Outcome						
**The overall incidence of SIC.**	37	53.0%	0.852	<0.0001		
Covariate						
Duration of sufentanil injection	37	70.92%	0.872		-0.114 (-0.592 to 0.364)	0.633
Dosage of sufentanil injection	37	69.65%	0.849		0.476 (-0.409 to 1.361)	0.284
ASA	37	71.17%	0.874		-0.114 (-1.869 to 1.642)	0.897
Outcome						
**The incidence of mild SIC.**	35	5.7%	0.368	<0.0001	-	-
Outcome						
**The incidence of moderate to severe SIC.**	35	30.6%	0.689	<0.0001		-
Covariate						
Duration of sufentanil injection	35	53.19%	0.705		0.039 (-0.494 to 0.572)	0.882
Dosage of sufentanil injection	35	50.81%	0.667		0.639 (-0.243 to 1.521)	0.150
ASA	35	53.44%	0.707		0.172 (-1.501 to 1.844)	0.836

Additional Figure

Figure S1 Risk of bias summary review authors' judgment about each risk of bias item for included RCTs.

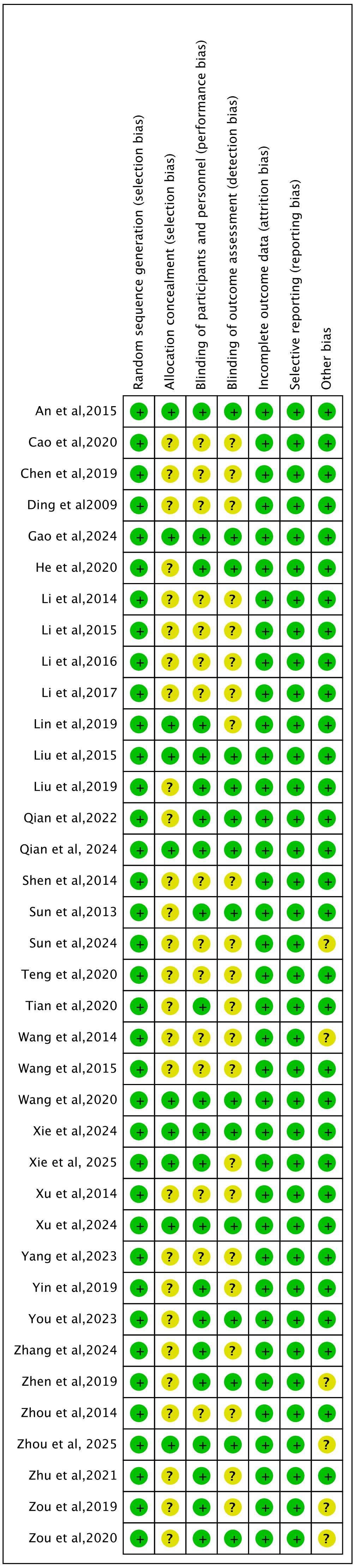

Figure S2 Funnel plot for the conventional pairwise meta-analysis

A. I Funnel plot of the overall incidence of SIC.

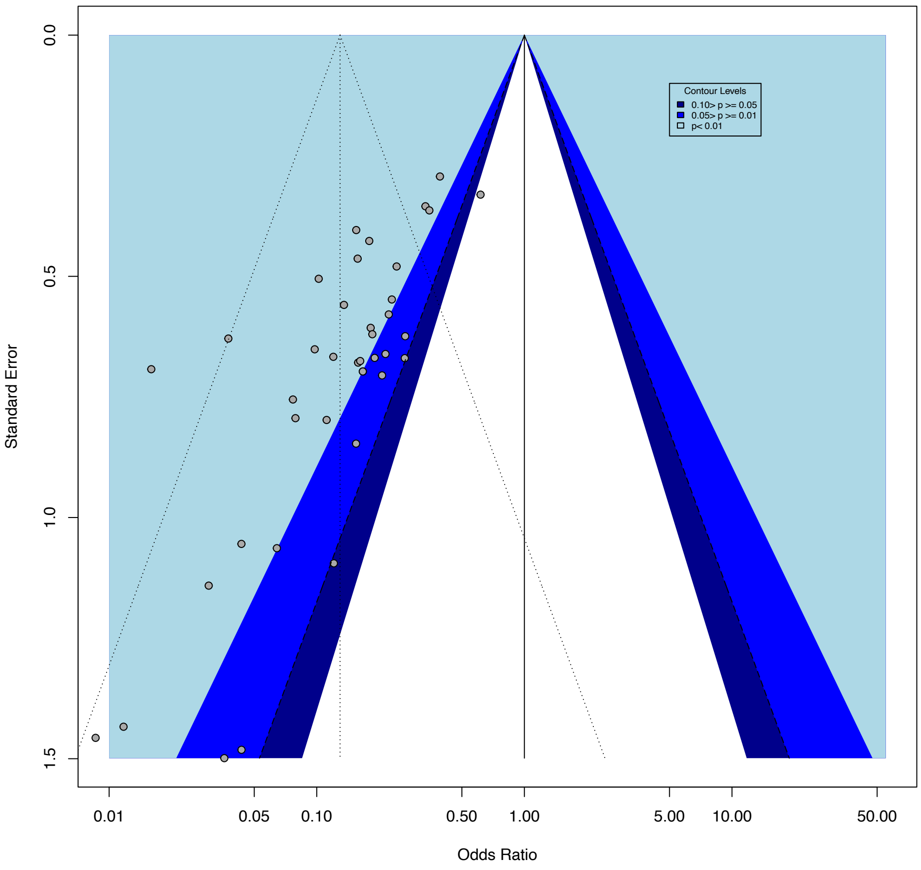

A.II Funnel plot of the overall incidence of SIC after Duval and Tweedie revision filling.

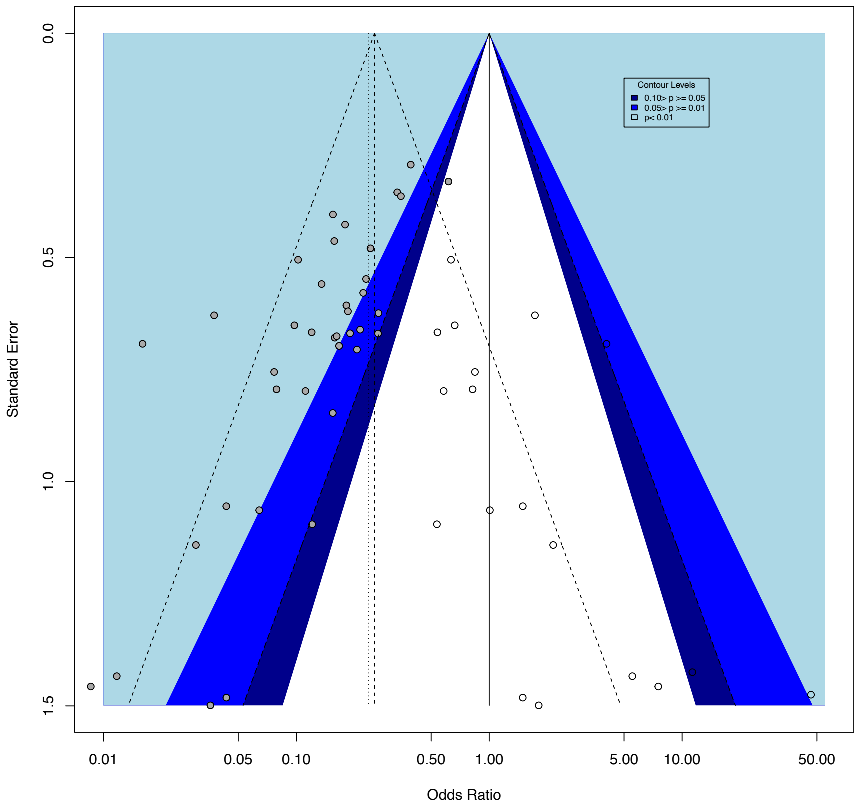

B. I Funnel plot of the incidence of mild SIC.

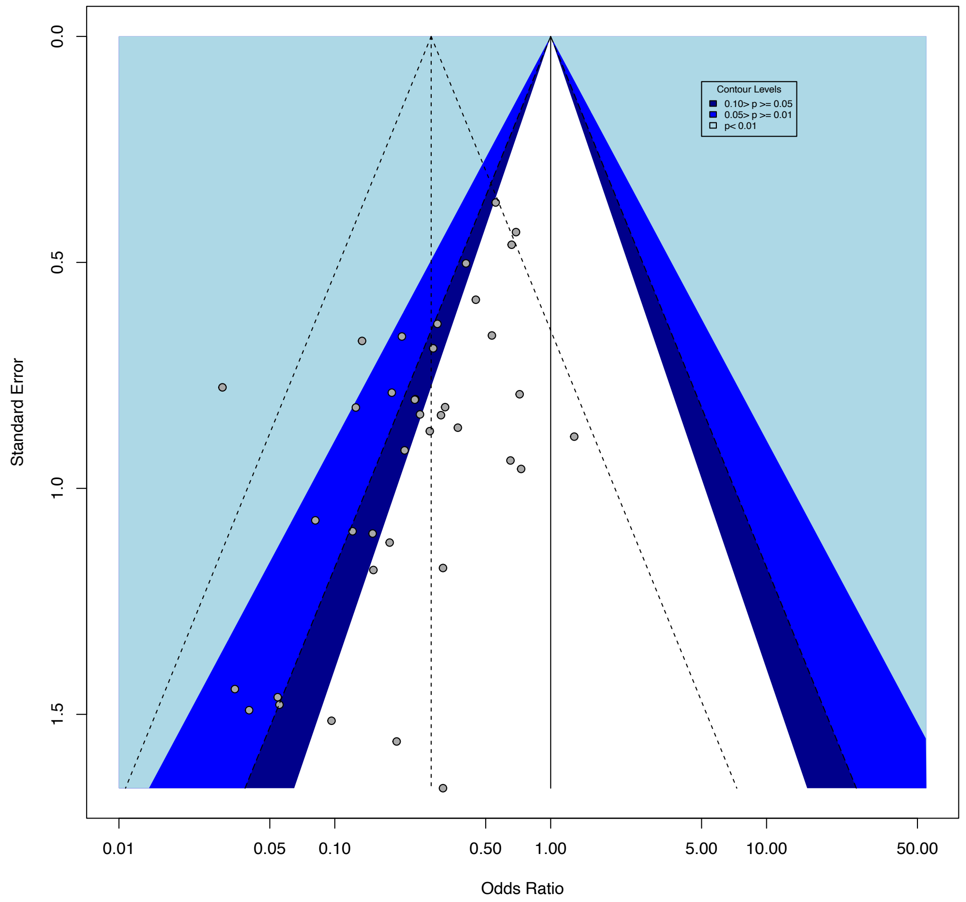

B.II Funnel plot of the incidence of mild SIC after Duval and Tweedie revision filling.

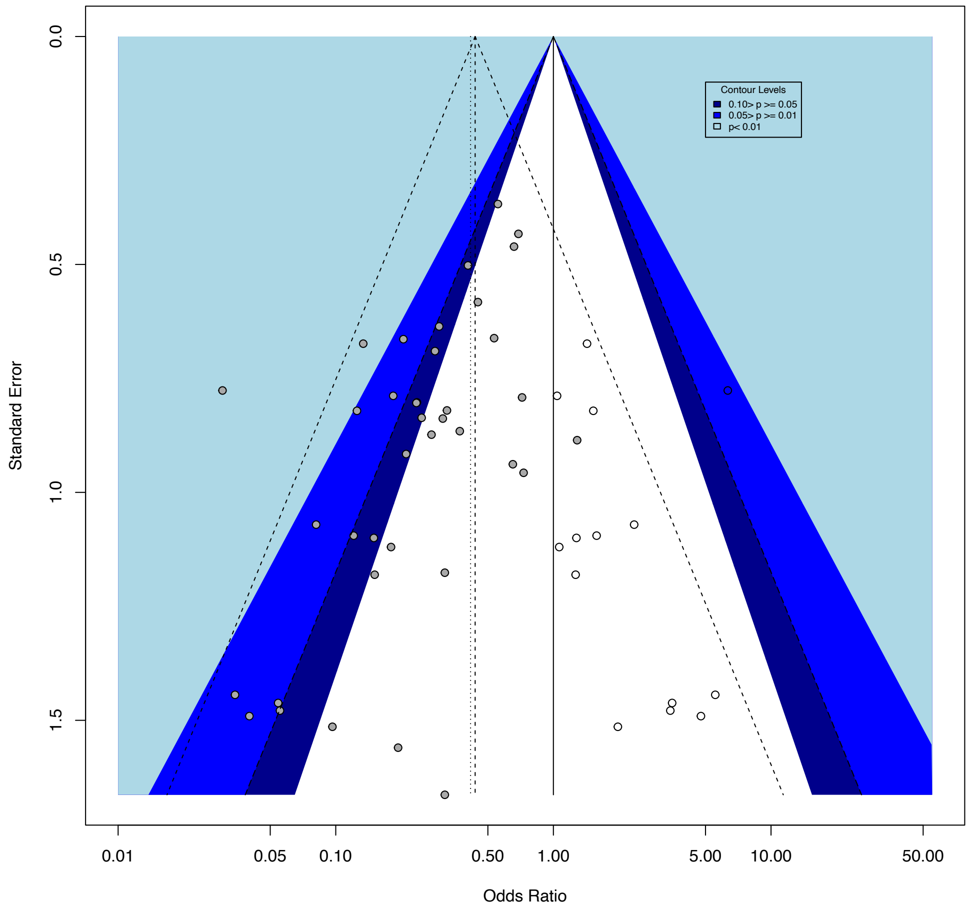

C. I Funnel plot of the incidence of moderate to severe SIC.

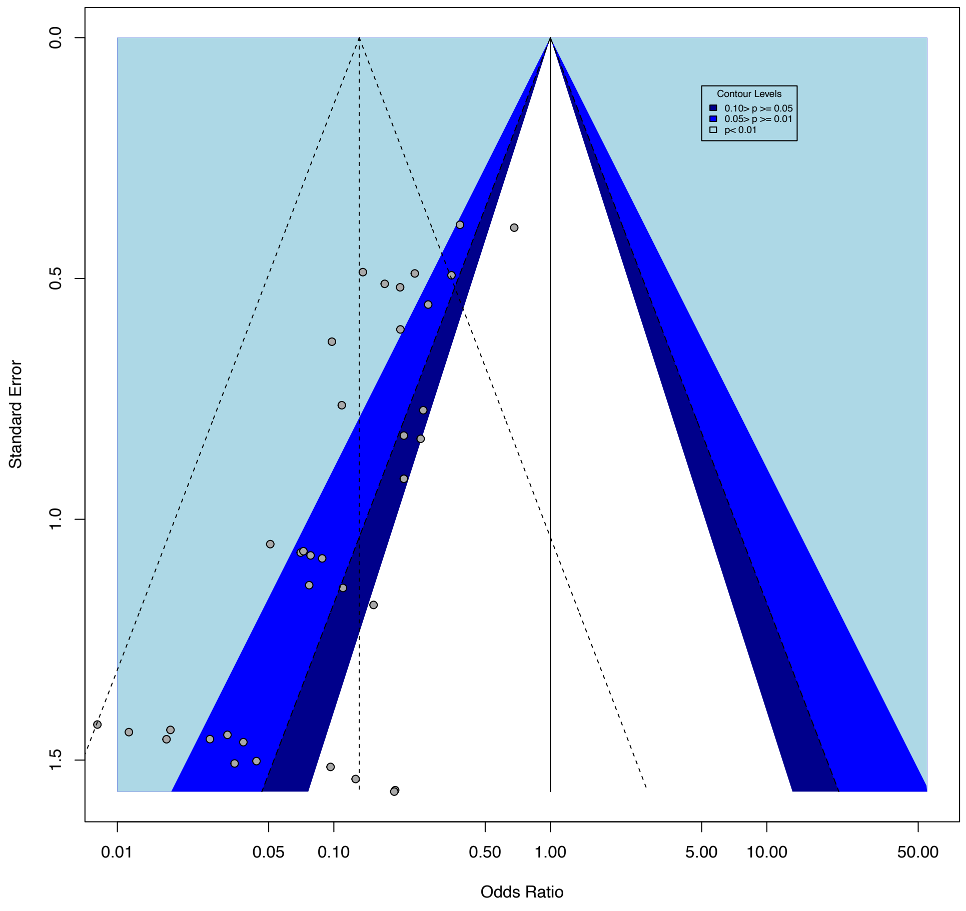

C.II Funnel plot of the incidence of moderate to severe SIC.

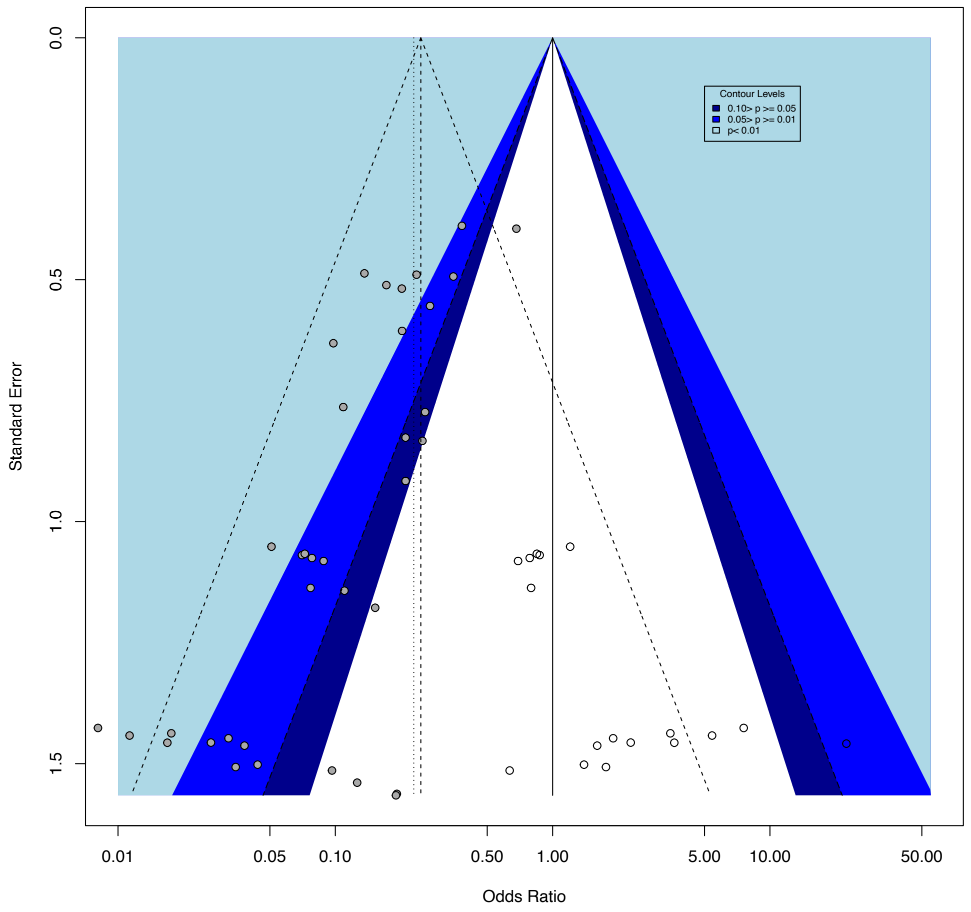

Figure S3 Forest plots of conventional pairwise meta-analysis.

A. I The overall incidence of SIC.

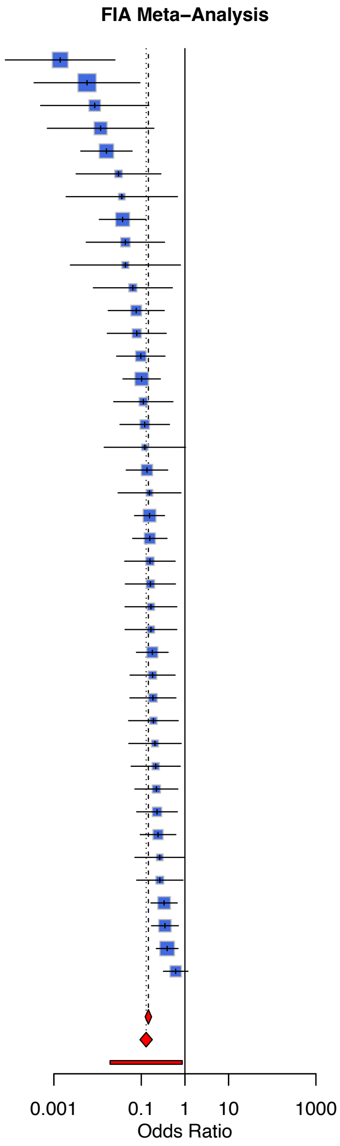

Study	Intervention Events	Intervention Total	Control Events	Control Total	OR	95%−CI	Weight (common)	Weight (random)
Yin et al 2019	0	80	33	40	0.00	[0.00; 0.03]	5.6%	1.1%
Liu et al 2015	0	185	59	185	0.01	[0.00; 0.09]	7.5%	1.2%
Li et al 2015	0	41	24	41	0.01	[0.00; 0.15]	3.0%	1.1%
Wang et al 2020	0	105	30	105	0.01	[0.00; 0.19]	3.8%	1.2%
Xu et al 2014	3	50	40	50	0.02	[0.00; 0.06]	4.7%	2.5%
Chen(1) et al 2019	1	30	8	15	0.03	[0.00; 0.28]	1.3%	1.6%
He et al 2020	0	20	8	20	0.04	[0.00; 0.68]	1.0%	1.1%
Li et al 2017	3	160	27	80	0.04	[0.01; 0.13]	4.4%	2.7%
Wang et al 2015	1	50	16	50	0.04	[0.01; 0.34]	2.0%	1.7%
Zhou et al 2014	0	30	8	30	0.04	[0.00; 0.79]	1.1%	1.1%
Yang et al 2023	1	49	12	49	0.06	[0.01; 0.52]	1.5%	1.7%
Liu et al 2019	2	100	21	100	0.08	[0.02; 0.34]	2.6%	2.4%
Xu et al 2024	2	40	16	40	0.08	[0.02; 0.37]	1.9%	2.3%
Zhang et al 2024	3	60	21	60	0.10	[0.03; 0.35]	2.5%	2.6%
Gao et al 2024	5	100	34	100	0.10	[0.04; 0.27]	4.1%	3.0%
Lin et al 2019	2	42	13	42	0.11	[0.02; 0.53]	1.6%	2.3%
Zhou et al 2025	3	50	17	49	0.12	[0.03; 0.44]	2.0%	2.6%
Teng et al 2020	1	40	7	40	0.12	[0.01; 1.03]	0.9%	1.6%
Zou et al 2019	4	110	24	110	0.14	[0.05; 0.40]	2.9%	2.9%
Li et al 2014	2	25	9	25	0.15	[0.03; 0.81]	1.0%	2.1%
An et al 2015	13	107	25	53	0.15	[0.07; 0.34]	3.7%	3.3%
Xie et al 2024	15	67	22	34	0.16	[0.06; 0.39]	2.8%	3.2%
Tian et al 2020	3	45	14	45	0.16	[0.04; 0.60]	1.6%	2.6%
Sun et al 2024	3	48	14	48	0.16	[0.04; 0.61]	1.6%	2.6%
Chen(2) et al 2019	6	30	9	15	0.17	[0.04; 0.65]	1.2%	2.5%
Wang(1) et al 2014	4	40	8	20	0.17	[0.04; 0.65]	1.2%	2.5%
Sun et al 2013	11	180	16	60	0.18	[0.08; 0.41]	2.8%	3.3%
Zhu(2) et al 2021	5	60	10	30	0.18	[0.06; 0.60]	1.5%	2.8%
Li et al 2016	4	40	15	40	0.19	[0.05; 0.62]	1.7%	2.7%
Xie et al 2025	4	54	8	27	0.19	[0.05; 0.71]	1.2%	2.6%
Cao(1) et al 2020	4	40	7	20	0.21	[0.05; 0.82]	1.1%	2.5%
Wang(2) et al 2014	5	40	8	20	0.21	[0.06; 0.78]	1.2%	2.6%
Zhu(1) et al 2021	6	60	10	30	0.22	[0.07; 0.69]	1.5%	2.8%
Shen et al 2014	5	60	17	60	0.23	[0.08; 0.67]	2.0%	2.9%
Zhen et al 2019	8	50	22	50	0.24	[0.09; 0.62]	2.3%	3.1%
Cao(2) et al 2020	5	40	7	20	0.27	[0.07; 0.99]	1.0%	2.6%
You et al 2023	4	44	12	44	0.27	[0.08; 0.91]	1.4%	2.7%
Qian et al 2022	15	93	34	93	0.33	[0.17; 0.67]	3.6%	3.4%
Zou et al 2020	12	152	30	152	0.35	[0.17; 0.71]	3.5%	3.4%
Qian et al 2025	25	118	48	118	0.39	[0.22; 0.70]	4.8%	3.6%
Ding et al 2009	25	80	34	80	0.61	[0.32; 1.18]	2.9%	3.5%
**Common effect model**		**2815**		**2290**	**0.15**	**[0.12; 0.17]**	**100.0%**	**.**
**Random effects model**					**0.13**	**[0.09; 0.18]**	**.**	**100.0%**
**Prediction interval**						**[0.02; 0.87]**		

Heterogeneity: $I^2 = 53.0\%$, $\tau^2 = 0.8524$, $p < 0.0001$

A.II The overall incidence of SIC after Duval and Tweedie revision filling.

Study	Events	Total	Events	Total	OR	95%-CI	Weight (common)	Weight (random)
Yin et al 2019	0	80	33	40	0.00	[0.00; 0.03]	0.3%	1.1%
Liu et al 2015	0	185	59	185	0.01	[0.00; 0.09]	0.3%	1.2%
Li et al 2015	0	41	24	41	0.01	[0.00; 0.15]	0.3%	1.2%
Wang et al 2020	0	105	30	105	0.01	[0.00; 0.19]	0.3%	1.2%
Xu et al 2014	3	50	40	50	0.02	[0.00; 0.06]	1.5%	1.8%
Chen(1) et al 2019	1	30	8	15	0.03	[0.00; 0.28]	0.5%	1.4%
He et al 2020	0	20	8	20	0.04	[0.00; 0.68]	0.3%	1.1%
Li et al 2017	3	160	27	80	0.04	[0.01; 0.13]	1.8%	1.9%
Wang et al 2015	1	50	16	50	0.04	[0.01; 0.34]	0.6%	1.5%
Zhou et al 2014	0	30	8	30	0.04	[0.00; 0.79]	0.3%	1.1%
Yang et al 2023	1	49	12	49	0.06	[0.01; 0.52]	0.6%	1.5%
Liu et al 2019	2	100	21	100	0.08	[0.02; 0.34]	1.2%	1.8%
Xu et al 2024	2	40	16	40	0.08	[0.02; 0.37]	1.1%	1.7%
Zhang et al 2024	3	60	21	60	0.10	[0.03; 0.35]	1.7%	1.9%
Gao et al 2024	5	100	34	100	0.10	[0.04; 0.27]	2.8%	2.0%
Lin et al 2019	2	42	13	42	0.11	[0.02; 0.53]	1.1%	1.7%
Zhou et al 2025	3	50	17	49	0.12	[0.03; 0.44]	1.6%	1.9%
Teng et al 2020	1	40	7	40	0.12	[0.01; 1.03]	0.6%	1.5%
Zou et al 2019	4	110	24	110	0.14	[0.05; 0.40]	2.3%	2.0%
Li et al 2014	2	25	9	25	0.15	[0.03; 0.81]	1.0%	1.7%
An et al 2015	13	107	25	53	0.15	[0.07; 0.34]	4.3%	2.1%
Xie et al 2024	15	67	22	34	0.16	[0.06; 0.39]	3.3%	2.0%
Tian et al 2020	3	45	14	45	0.16	[0.04; 0.60]	1.5%	1.9%
Sun et al 2024	3	48	14	48	0.16	[0.04; 0.61]	1.5%	1.9%
Chen(2) et al 2019	6	30	9	15	0.17	[0.04; 0.65]	1.5%	1.8%
Wang(1) et al 2014	4	40	8	20	0.17	[0.04; 0.65]	1.5%	1.8%
Sun et al 2013	11	180	16	60	0.18	[0.08; 0.41]	3.9%	2.1%
Zhu(2) et al 2021	5	60	10	30	0.18	[0.06; 0.60]	1.9%	1.9%
Li et al 2016	4	40	15	40	0.19	[0.05; 0.62]	1.8%	1.9%
Xie et al 2025	4	54	8	27	0.19	[0.05; 0.71]	1.6%	1.9%
Cao(1) et al 2020	4	40	7	20	0.21	[0.05; 0.82]	1.4%	1.8%
Wang(2) et al 2014	5	40	8	20	0.21	[0.06; 0.78]	1.6%	1.9%
Zhu(1) et al 2021	6	60	10	30	0.22	[0.07; 0.69]	2.1%	1.9%
Shen et al 2014	5	60	17	60	0.23	[0.08; 0.67]	2.4%	2.0%
Zhen et al 2019	8	50	22	50	0.24	[0.09; 0.62]	3.1%	2.0%
Cao(2) et al 2020	5	40	7	20	0.27	[0.07; 0.99]	1.6%	1.9%
You et al 2023	4	44	12	44	0.27	[0.08; 0.91]	1.8%	1.9%
Qian et al 2022	15	93	34	93	0.33	[0.17; 0.67]	5.6%	2.1%
Zou et al 2020	12	152	30	152	0.35	[0.17; 0.71]	5.4%	2.1%
Qian et al 2025	25	118	48	118	0.39	[0.22; 0.70]	8.2%	2.1%
Filled: Teng et al 2020	1	40	7	40	0.54	[0.06; 4.58]	0.6%	1.5%
Filled: Zhou et al 2025	3	50	17	49	0.54	[0.15; 1.99]	1.6%	1.9%
Filled: Lin et al 2019	2	42	13	42	0.58	[0.12; 2.77]	1.1%	1.7%
Ding et al 2009	25	80	34	80	0.61	[0.32; 1.18]	6.5%	2.1%
Filled: Gao et al 2024	5	100	34	100	0.63	[0.24; 1.71]	2.8%	2.0%
Filled: Zhang et al 2024	3	60	21	60	0.66	[0.18; 2.37]	1.7%	1.9%
Filled: Xu et al 2024	2	40	16	40	0.82	[0.17; 3.89]	1.1%	1.7%
Filled: Liu et al 2019	2	100	21	100	0.84	[0.19; 3.71]	1.2%	1.8%
Filled: Yang et al 2023	1	49	12	49	1.01	[0.13; 8.11]	0.6%	1.5%
Filled: Zhou et al 2014	0	30	8	30	1.49	[0.08; 27.22]	0.3%	1.1%
Filled: Wang et al 2015	1	50	16	50	1.49	[0.19; 11.80]	0.6%	1.5%
Filled: Li et al 2017	3	160	27	80	1.73	[0.50; 5.92]	1.8%	1.9%
Filled: He et al 2020	0	20	8	20	1.81	[0.10; 34.07]	0.3%	1.1%
Filled: Chen(1) et al 2019	1	30	8	15	2.15	[0.23; 20.09]	0.5%	1.4%
Filled: Xu et al 2014	3	50	40	50	4.06	[1.04; 15.77]	1.5%	1.8%
Filled: Wang et al 2020	0	105	30	105	5.52	[0.33; 91.68]	0.3%	1.2%
Filled: Li et al 2015	0	41	24	41	7.52	[0.43; 130.76]	0.3%	1.2%
Filled: Liu et al 2015	0	185	59	185	11.30	[0.69; 184.46]	0.3%	1.2%
Filled: Yin et al 2019	0	80	33	40	46.57	[2.59; 838.71]	0.3%	1.1%
**Common effect model**		**4047**		**3386**	**0.25**	**[0.22; 0.30]**	**100.0%**	.
**Random effects model**					**0.24**	**[0.16; 0.36]**	.	**100.0%**
**Prediction interval**						**[0.01; 5.07]**		

Heterogeneity: $I^2 = 67.0\%$, $\tau^2 = 2.2897$, $p < 0.0001$

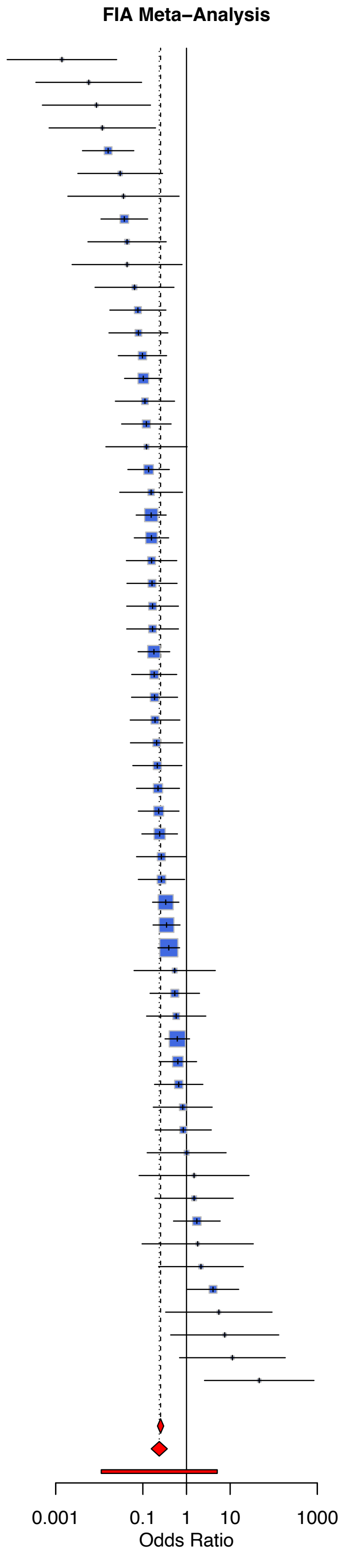

A.III The overall incidence of SIC after exclusion of outliers.

Outliers: "Yin et al 2019", "Ding et al 2009", "Xu et al 2014", "Qian et al 2025"

Study	Intervention Events	Intervention Total	Control Events	Control Total	OR	95%−CI	Weight (common)	Weight (random)
Liu et al 2015	0	185	59	185	0.01	[0.00; 0.09]	9.1%	1.0%
Li et al 2015	0	41	24	41	0.01	[0.00; 0.15]	3.7%	1.0%
Wang et al 2020	0	105	30	105	0.01	[0.00; 0.19]	4.6%	1.0%
Chen(1) et al 2019	1	30	8	15	0.03	[0.00; 0.28]	1.6%	1.4%
He et al 2020	0	20	8	20	0.04	[0.00; 0.68]	1.3%	0.9%
Li et al 2017	3	160	27	80	0.04	[0.01; 0.13]	5.4%	3.0%
Wang et al 2015	1	50	16	50	0.04	[0.01; 0.34]	2.4%	1.6%
Zhou et al 2014	0	30	8	30	0.04	[0.00; 0.79]	1.3%	1.0%
Yang et al 2023	1	49	12	49	0.06	[0.01; 0.52]	1.8%	1.6%
Liu et al 2019	2	100	21	100	0.08	[0.02; 0.34]	3.2%	2.5%
Xu et al 2024	2	40	16	40	0.08	[0.02; 0.37]	2.3%	2.4%
Zhang et al 2024	3	60	21	60	0.10	[0.03; 0.35]	3.1%	2.9%
Gao et al 2024	5	100	34	100	0.10	[0.04; 0.27]	4.9%	3.7%
Lin et al 2019	2	42	13	42	0.11	[0.02; 0.53]	1.9%	2.4%
Zhou et al 2025	3	50	17	49	0.12	[0.03; 0.44]	2.5%	2.9%
Teng et al 2020	1	40	7	40	0.12	[0.01; 1.03]	1.0%	1.5%
Zou et al 2019	4	110	24	110	0.14	[0.05; 0.40]	3.5%	3.4%
Li et al 2014	2	25	9	25	0.15	[0.03; 0.81]	1.3%	2.2%
An et al 2015	13	107	25	53	0.15	[0.07; 0.34]	4.5%	4.2%
Xie et al 2024	15	67	22	34	0.16	[0.06; 0.39]	3.5%	3.9%
Tian et al 2020	3	45	14	45	0.16	[0.04; 0.60]	2.0%	2.8%
Sun et al 2024	3	48	14	48	0.16	[0.04; 0.61]	2.0%	2.8%
Chen(2) et al 2019	6	30	9	15	0.17	[0.04; 0.65]	1.5%	2.7%
Wang(1) et al 2014	4	40	8	20	0.17	[0.04; 0.65]	1.5%	2.7%
Sun et al 2013	11	180	16	60	0.18	[0.08; 0.41]	3.5%	4.1%
Zhu(2) et al 2021	5	60	10	30	0.18	[0.06; 0.60]	1.9%	3.1%
Li et al 2016	4	40	15	40	0.19	[0.05; 0.62]	2.1%	3.1%
Xie et al 2025	4	54	8	27	0.19	[0.05; 0.71]	1.5%	2.9%
Cao(1) et al 2020	4	40	7	20	0.21	[0.05; 0.82]	1.3%	2.7%
Wang(2) et al 2014	5	40	8	20	0.21	[0.06; 0.78]	1.4%	2.9%
Zhu(1) et al 2021	6	60	10	30	0.22	[0.07; 0.69]	1.8%	3.3%
Shen et al 2014	5	60	17	60	0.23	[0.08; 0.67]	2.4%	3.4%
Zhen et al 2019	8	50	22	50	0.24	[0.09; 0.62]	2.8%	3.8%
Cao(2) et al 2020	5	40	7	20	0.27	[0.07; 0.99]	1.3%	2.9%
You et al 2023	4	44	12	44	0.27	[0.08; 0.91]	1.7%	3.1%
Qian et al 2022	15	93	34	93	0.33	[0.17; 0.67]	4.4%	4.5%
Zou et al 2020	12	152	30	152	0.35	[0.17; 0.71]	4.2%	4.5%
**Common effect model**		**2487**		**2002**	**0.13**	**[0.11; 0.16]**	**100.0%**	.
**Random effects model**					**0.14**	**[0.11; 0.18]**	.	**100.0%**
**Prediction interval**						**[0.04; 0.54]**		

Heterogeneity: $I^2 = 10.3\%$, $\tau^2 = 0.4253$, $p = 0.2915$

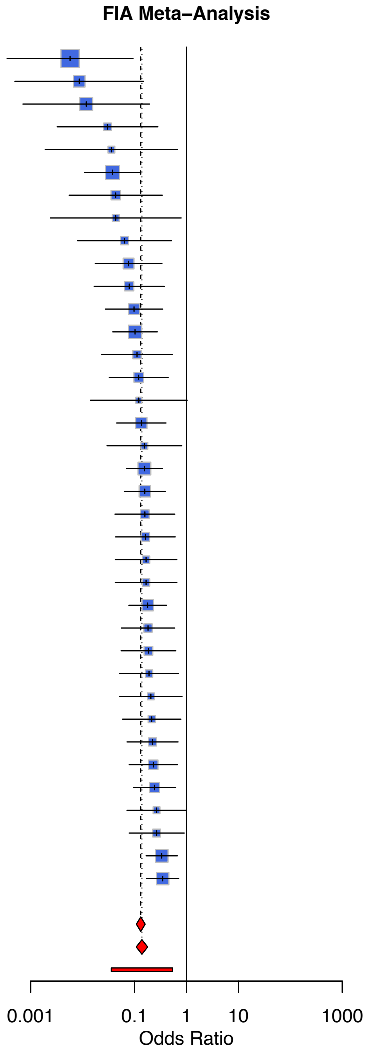

B.I The incidence of mild SIC.

Study	Intervention Events	Intervention Total	Control Events	Control Total	OR	95%−CI	Weight (common)	Weight (random)
Xu et al 2014	2	50	29	50	0.03	[0.01; 0.14]	9.4%	3.0%
Liu et al 2015	0	185	13	185	0.03	[0.00; 0.58]	4.5%	1.2%
Yin et al 2019	0	80	5	40	0.04	[0.00; 0.74]	2.4%	1.1%
Wang et al 2020	0	105	8	105	0.05	[0.00; 0.95]	2.8%	1.1%
Li et al 2015	0	41	7	41	0.06	[0.00; 1.01]	2.5%	1.1%
Yang et al 2023	1	49	10	49	0.08	[0.01; 0.66]	3.3%	1.9%
Zhou et al 2014	0	30	4	30	0.10	[0.00; 1.88]	1.5%	1.1%
Xu et al 2024	1	40	7	40	0.12	[0.01; 1.03]	2.3%	1.8%
An et al 2015	2	107	7	53	0.13	[0.03; 0.63]	3.1%	2.7%
Li et al 2017	3	160	10	80	0.13	[0.04; 0.50]	4.4%	3.5%
Wang et al 2015	1	50	6	50	0.15	[0.02; 1.29]	2.0%	1.8%
Xie et al 2025	1	54	3	27	0.15	[0.01; 1.53]	1.3%	1.6%
Teng et al 2020	1	40	5	40	0.18	[0.02; 1.61]	1.6%	1.8%
Gao et al 2024	2	100	10	100	0.18	[0.04; 0.86]	3.3%	2.9%
Shen et al 2014	0	60	2	60	0.19	[0.01; 4.11]	0.8%	1.0%
Sun et al 2013	4	180	6	60	0.20	[0.06; 0.75]	3.0%	3.5%
Wang(1) et al 2014	2	40	4	20	0.21	[0.03; 1.27]	1.7%	2.4%
Liu et al 2019	2	100	8	100	0.23	[0.05; 1.13]	2.6%	2.8%
Zou et al 2019	2	110	8	110	0.24	[0.05; 1.14]	2.6%	2.8%
Li et al 2016	2	40	7	40	0.25	[0.05; 1.28]	2.2%	2.7%
Li et al 2014	2	25	6	25	0.28	[0.05; 1.53]	1.9%	2.5%
Zhu(2) et al 2021	4	60	6	30	0.29	[0.07; 1.10]	2.5%	3.4%
Zhu(1) et al 2021	5	60	7	30	0.30	[0.09; 1.04]	2.9%	3.7%
Zhang et al 2024	2	60	6	60	0.31	[0.06; 1.60]	2.0%	2.7%
Lin et al 2019	1	42	3	42	0.32	[0.03; 3.18]	1.0%	1.6%
He et al 2020	0	20	1	20	0.32	[0.01; 8.26]	0.5%	0.9%
Wang(2) et al 2014	3	40	4	20	0.32	[0.06; 1.62]	1.7%	2.8%
You et al 2023	2	44	5	44	0.37	[0.07; 2.03]	1.6%	2.6%
Zou et al 2020	6	152	14	152	0.41	[0.15; 1.08]	4.5%	4.6%
Xie et al 2024	7	67	7	34	0.45	[0.14; 1.41]	2.8%	4.1%
Zhen et al 2019	4	50	7	50	0.53	[0.15; 1.95]	2.2%	3.6%
Qian et al 2025	14	118	23	118	0.56	[0.27; 1.14]	6.8%	5.7%
Tian et al 2020	2	45	3	45	0.65	[0.10; 4.10]	1.0%	2.3%
Qian et al 2022	9	93	13	93	0.66	[0.27; 1.63]	4.0%	4.9%
Ding et al 2009	11	80	15	80	0.69	[0.30; 1.61]	4.4%	5.2%
Zhou et al 2025	3	50	4	49	0.72	[0.15; 3.39]	1.3%	2.9%
Cao(1) et al 2020	3	40	2	20	0.73	[0.11; 4.76]	0.8%	2.2%
Cao(2) et al 2020	5	40	2	20	1.29	[0.23; 7.29]	0.8%	2.5%
**Common effect model**		**2707**		**2212**	**0.28**	**[0.22; 0.35]**	**100.0%**	**.**
**Random effects model**					**0.28**	**[0.21; 0.36]**	**.**	**100.0%**
**Prediction interval**						**[0.08; 0.99]**		

Heterogeneity: $I^2 = 5.7\%$, $\tau^2 = 0.3682$, $p = 0.3693$

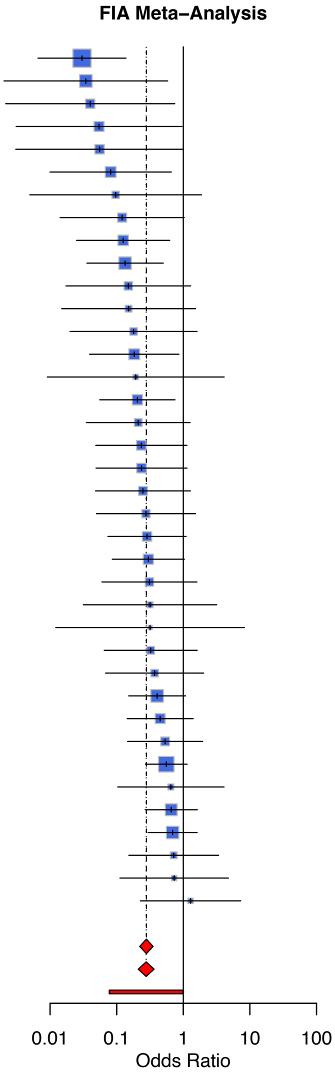

B.II The incidence of mild SIC after Duval and Tweedie revision filling.

Study	Events	Total	Events	Total	OR	95%-CI	Weight (common)	Weight (random)
Xu et al 2014	2	50	29	50	0.03	[0.01; 0.14]	2.1%	2.3%
Liu et al 2015	0	185	13	185	0.03	[0.00; 0.58]	0.6%	1.2%
Yin et al 2019	0	80	5	40	0.04	[0.00; 0.74]	0.6%	1.1%
Wang et al 2020	0	105	8	105	0.05	[0.00; 0.95]	0.6%	1.2%
Li et al 2015	0	41	7	41	0.06	[0.00; 1.01]	0.6%	1.1%
Yang et al 2023	1	49	10	49	0.08	[0.01; 0.66]	1.1%	1.7%
Zhou et al 2014	0	30	4	30	0.10	[0.00; 1.88]	0.6%	1.1%
Xu et al 2024	1	40	7	40	0.12	[0.01; 1.03]	1.1%	1.7%
An et al 2015	2	107	7	53	0.13	[0.03; 0.63]	1.9%	2.2%
Li et al 2017	3	160	10	80	0.13	[0.04; 0.50]	2.8%	2.5%
Wang et al 2015	1	50	6	50	0.15	[0.02; 1.29]	1.1%	1.6%
Xie et al 2025	1	54	3	27	0.15	[0.01; 1.53]	0.9%	1.5%
Teng et al 2020	1	40	5	40	0.18	[0.02; 1.61]	1.0%	1.6%
Gao et al 2024	2	100	10	100	0.18	[0.04; 0.86]	2.1%	2.3%
Shen et al 2014	0	60	2	60	0.19	[0.01; 4.11]	0.5%	1.1%
Sun et al 2013	4	180	6	60	0.20	[0.06; 0.75]	2.9%	2.5%
Wang(1) et al 2014	2	40	4	20	0.21	[0.03; 1.27]	1.5%	2.0%
Liu et al 2019	2	100	8	100	0.23	[0.05; 1.13]	2.0%	2.2%
Zou et al 2019	2	110	8	110	0.24	[0.05; 1.14]	2.0%	2.2%
Li et al 2016	2	40	7	40	0.25	[0.05; 1.28]	1.8%	2.1%
Li et al 2014	2	25	6	25	0.28	[0.05; 1.53]	1.7%	2.1%
Zhu(2) et al 2021	4	60	6	30	0.29	[0.07; 1.10]	2.7%	2.5%
Zhu(1) et al 2021	5	60	7	30	0.30	[0.09; 1.04]	3.2%	2.6%
Zhang et al 2024	2	60	6	60	0.31	[0.06; 1.60]	1.8%	2.1%
Lin et al 2019	1	42	3	42	0.32	[0.03; 3.18]	0.9%	1.5%
He et al 2020	0	20	1	20	0.32	[0.01; 8.26]	0.5%	1.0%
Wang(2) et al 2014	3	40	4	20	0.32	[0.06; 1.62]	1.9%	2.2%
You et al 2023	2	44	5	44	0.37	[0.07; 2.03]	1.7%	2.1%
Zou et al 2020	6	152	14	152	0.41	[0.15; 1.08]	5.1%	2.9%
Xie et al 2024	7	67	7	34	0.45	[0.14; 1.41]	3.8%	2.7%
Zhen et al 2019	4	50	7	50	0.53	[0.15; 1.95]	2.9%	2.5%
Qian et al 2025	14	118	23	118	0.56	[0.27; 1.14]	9.5%	3.2%
Tian et al 2020	2	45	3	45	0.65	[0.10; 4.10]	1.5%	1.9%
Qian et al 2022	9	93	13	93	0.66	[0.27; 1.63]	6.0%	3.0%
Ding et al 2009	11	80	15	80	0.69	[0.30; 1.61]	6.8%	3.1%
Zhou et al 2025	3	50	4	49	0.72	[0.15; 3.39]	2.0%	2.2%
Cao(1) et al 2020	3	40	2	20	0.73	[0.11; 4.76]	1.4%	1.9%
Filled: Gao et al 2024	2	100	10	100	1.04	[0.22; 4.87]	2.1%	2.3%
Filled: Teng et al 2020	1	40	5	40	1.06	[0.12; 9.55]	1.0%	1.6%
Filled: Xie et al 2025	1	54	3	27	1.27	[0.13; 12.80]	0.9%	1.5%
Filled: Wang et al 2015	1	50	6	50	1.28	[0.15; 11.02]	1.1%	1.6%
Cao(2) et al 2020	5	40	2	20	1.29	[0.23; 7.29]	1.6%	2.0%
Filled: Li et al 2017	3	160	10	80	1.43	[0.38; 5.35]	2.8%	2.5%
Filled: An et al 2015	2	107	7	53	1.53	[0.31; 7.63]	1.9%	2.2%
Filled: Xu et al 2024	1	40	7	40	1.58	[0.18; 13.51]	1.1%	1.7%
Filled: Zhou et al 2014	0	30	4	30	1.98	[0.10; 38.47]	0.6%	1.1%
Filled: Yang et al 2023	1	49	10	49	2.35	[0.29; 19.17]	1.1%	1.7%
Filled: Li et al 2015	0	41	7	41	3.45	[0.19; 62.50]	0.6%	1.1%
Filled: Wang et al 2020	0	105	8	105	3.51	[0.20; 61.67]	0.6%	1.2%
Filled: Yin et al 2019	0	80	5	40	4.76	[0.26; 88.49]	0.6%	1.1%
Filled: Liu et al 2015	0	185	13	185	5.54	[0.33; 93.98]	0.6%	1.2%
Filled: Xu et al 2014	2	50	29	50	6.33	[1.38; 28.99]	2.1%	2.3%
**Common effect model**		**3798**		**3102**	**0.44**	**[0.35; 0.55]**	**100.0%**	.
**Random effects model**					**0.42**	**[0.30; 0.58]**	.	**100.0%**
**Prediction interval**						**[0.06; 3.12]**		

Heterogeneity: $I^2 = 36.9\%$, $\tau^2 = 0.9691$, $p = 0.0049$

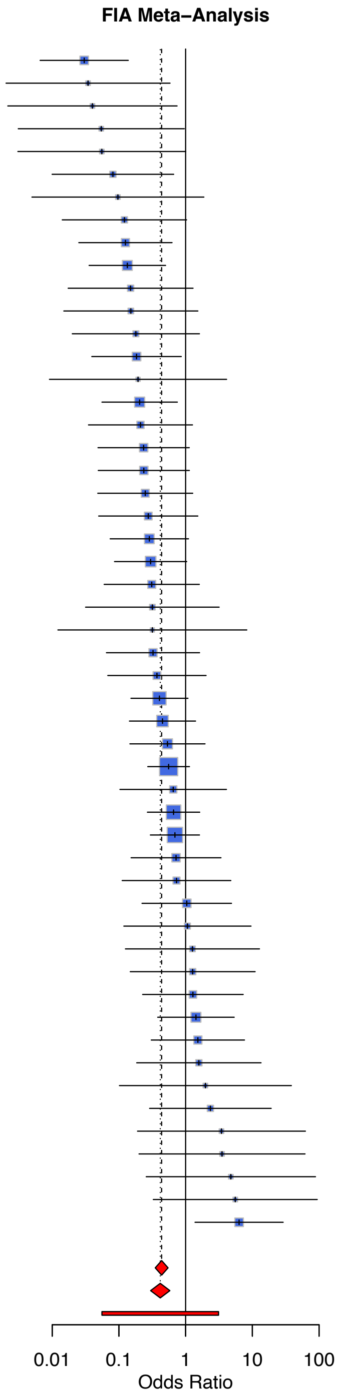

B.III The incidence of mild SIC after exclusion of outliers.

Outlier: "Xu et al 2014"

Study	Intervention Events	Intervention Total	Control Events	Control Total	OR	95%−CI	Weight (common)	Weight (random)
Liu et al 2015	0	185	13	185	0.03	[0.00; 0.58]	5.0%	1.1%
Yin et al 2019	0	80	5	40	0.04	[0.00; 0.74]	2.7%	1.1%
Wang et al 2020	0	105	8	105	0.05	[0.00; 0.95]	3.1%	1.1%
Li et al 2015	0	41	7	41	0.06	[0.00; 1.01]	2.8%	1.1%
Yang et al 2023	1	49	10	49	0.08	[0.01; 0.66]	3.6%	1.9%
Zhou et al 2014	0	30	4	30	0.10	[0.00; 1.88]	1.6%	1.0%
Xu et al 2024	1	40	7	40	0.12	[0.01; 1.03]	2.5%	1.8%
An et al 2015	2	107	7	53	0.13	[0.03; 0.63]	3.4%	2.8%
Li et al 2017	3	160	10	80	0.13	[0.04; 0.50]	4.9%	3.6%
Wang et al 2015	1	50	6	50	0.15	[0.02; 1.29]	2.2%	1.8%
Xie et al 2025	1	54	3	27	0.15	[0.01; 1.53]	1.5%	1.6%
Teng et al 2020	1	40	5	40	0.18	[0.02; 1.61]	1.8%	1.7%
Gao et al 2024	2	100	10	100	0.18	[0.04; 0.86]	3.6%	3.0%
Shen et al 2014	0	60	2	60	0.19	[0.01; 4.11]	0.9%	1.0%
Sun et al 2013	4	180	6	60	0.20	[0.06; 0.75]	3.3%	3.7%
Wang(1) et al 2014	2	40	4	20	0.21	[0.03; 1.27]	1.9%	2.4%
Liu et al 2019	2	100	8	100	0.23	[0.05; 1.13]	2.9%	2.9%
Zou et al 2019	2	110	8	110	0.24	[0.05; 1.14]	2.9%	2.9%
Li et al 2016	2	40	7	40	0.25	[0.05; 1.28]	2.5%	2.7%
Li et al 2014	2	25	6	25	0.28	[0.05; 1.53]	2.1%	2.6%
Zhu(2) et al 2021	4	60	6	30	0.29	[0.07; 1.10]	2.8%	3.5%
Zhu(1) et al 2021	5	60	7	30	0.30	[0.09; 1.04]	3.2%	3.9%
Zhang et al 2024	2	60	6	60	0.31	[0.06; 1.60]	2.2%	2.7%
Lin et al 2019	1	42	3	42	0.32	[0.03; 3.18]	1.1%	1.6%
He et al 2020	0	20	1	20	0.32	[0.01; 8.26]	0.5%	0.9%
Wang(2) et al 2014	3	40	4	20	0.32	[0.06; 1.62]	1.8%	2.8%
You et al 2023	2	44	5	44	0.37	[0.07; 2.03]	1.8%	2.6%
Zou et al 2020	6	152	14	152	0.41	[0.15; 1.08]	5.0%	5.0%
Xie et al 2024	7	67	7	34	0.45	[0.14; 1.41]	3.1%	4.3%
Zhen et al 2019	4	50	7	50	0.53	[0.15; 1.95]	2.4%	3.7%
Qian et al 2025	14	118	23	118	0.56	[0.27; 1.14]	7.5%	6.4%
Tian et al 2020	2	45	3	45	0.65	[0.10; 4.10]	1.1%	2.3%
Qian et al 2022	9	93	13	93	0.66	[0.27; 1.63]	4.4%	5.4%
Ding et al 2009	11	80	15	80	0.69	[0.30; 1.61]	4.8%	5.7%
Zhou et al 2025	3	50	4	49	0.72	[0.15; 3.39]	1.4%	2.9%
Cao(1) et al 2020	3	40	2	20	0.73	[0.11; 4.76]	0.9%	2.2%
Cao(2) et al 2020	5	40	2	20	1.29	[0.23; 7.29]	0.9%	2.5%
**Common effect model**		**2657**		**2162**	**0.31**	**[0.24; 0.39]**	**100.0%**	**.**
**Random effects model**					**0.30**	**[0.24; 0.39]**	**.**	**100.0%**
**Prediction interval**						**[0.10; 0.93]**		

Heterogeneity: $I^2 = 0.0\%$, $\tau^2 = 0.2834$, $p = 0.7594$

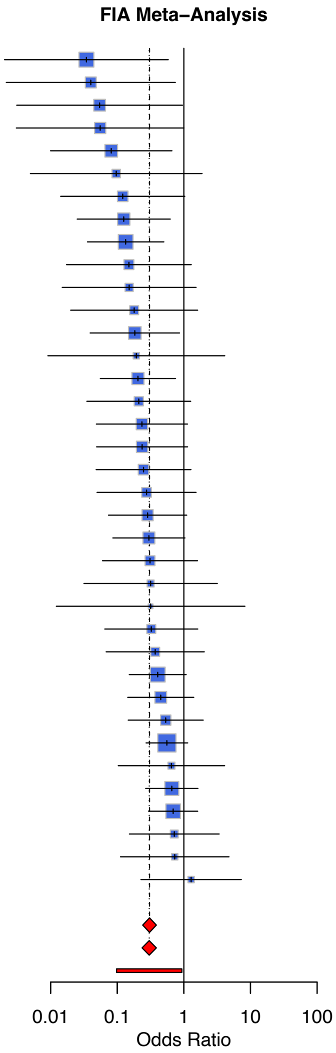

C.I The incidence of moderate to severe SIC.

Study	Intervention Events	Intervention Total	Control Events	Control Total	OR	95%-CI	Weight (common)	Weight (random)
Yin et al 2019	0	80	28	40	0.00	[0.00; 0.05]	7.4%	1.5%
Liu et al 2015	0	185	46	185	0.01	[0.00; 0.13]	9.1%	1.5%
Li et al 2017	0	160	17	80	0.01	[0.00; 0.19]	4.6%	1.5%
Li et al 2015	0	41	17	41	0.02	[0.00; 0.29]	3.4%	1.5%
Wang et al 2020	0	105	22	105	0.02	[0.00; 0.29]	4.4%	1.5%
Zhou et al 2025	0	50	13	49	0.03	[0.00; 0.46]	2.7%	1.5%
Liu et al 2019	0	100	13	100	0.03	[0.00; 0.55]	2.6%	1.5%
Cao(2) et al 2020	0	40	5	20	0.03	[0.00; 0.67]	1.4%	1.4%
Wang et al 2015	0	50	10	50	0.04	[0.00; 0.67]	2.1%	1.5%
He et al 2020	0	20	7	20	0.04	[0.00; 0.83]	1.4%	1.4%
Zhang et al 2024	1	60	15	60	0.05	[0.01; 0.40]	2.9%	2.3%
Tian et al 2020	1	45	11	45	0.07	[0.01; 0.57]	2.1%	2.3%
Xu et al 2014	1	50	11	50	0.07	[0.01; 0.58]	2.1%	2.3%
Cao(1) et al 2020	1	40	5	20	0.08	[0.01; 0.71]	1.3%	2.1%
Lin et al 2019	1	42	10	42	0.08	[0.01; 0.64]	1.9%	2.3%
Xu et al 2024	1	40	9	40	0.09	[0.01; 0.74]	1.7%	2.2%
Zhou et al 2014	0	30	4	30	0.10	[0.00; 1.88]	0.9%	1.4%
Gao et al 2024	3	100	24	100	0.10	[0.03; 0.34]	4.6%	3.8%
Zou et al 2019	2	110	16	110	0.11	[0.02; 0.49]	3.1%	3.3%
Zhu(2) et al 2021	1	60	4	30	0.11	[0.01; 1.03]	1.0%	2.1%
Li et al 2014	0	25	3	25	0.13	[0.01; 2.58]	0.7%	1.4%
An et al 2015	7	107	18	53	0.14	[0.05; 0.35]	4.4%	4.5%
Zhu(1) et al 2021	1	60	3	30	0.15	[0.02; 1.53]	0.8%	2.0%
Xie et al 2024	8	67	15	34	0.17	[0.06; 0.47]	3.5%	4.4%
Teng et al 2020	0	40	2	40	0.19	[0.01; 4.09]	0.5%	1.3%
Yang et al 2023	0	49	2	49	0.19	[0.01; 4.10]	0.5%	1.3%
Sun et al 2013	7	180	10	60	0.20	[0.07; 0.56]	2.8%	4.4%
Zhen et al 2019	4	50	15	50	0.20	[0.06; 0.67]	2.7%	4.0%
Li et al 2016	2	40	8	40	0.21	[0.04; 1.06]	1.5%	3.0%
Wang(1) et al 2014	2	40	4	20	0.21	[0.03; 1.27]	1.0%	2.7%
Wang(2) et al 2014	2	40	4	20	0.21	[0.03; 1.27]	1.0%	2.7%
Qian et al 2022	6	93	21	93	0.24	[0.09; 0.62]	3.9%	4.5%
You et al 2023	2	44	7	44	0.25	[0.05; 1.29]	1.3%	3.0%
Xie et al 2025	3	54	5	27	0.26	[0.06; 1.18]	1.2%	3.2%
Shen et al 2014	5	60	15	60	0.27	[0.09; 0.81]	2.7%	4.2%
Zou et al 2020	6	152	16	152	0.35	[0.13; 0.92]	3.0%	4.5%
Qian et al 2025	11	118	25	118	0.38	[0.18; 0.82]	4.5%	5.0%
Ding et al 2009	14	80	19	80	0.68	[0.31; 1.48]	3.1%	4.9%
**Common effect model**		**2707**		**2212**	**0.13**	**[0.10; 0.16]**	**100.0%**	.
**Random effects model**					**0.13**	**[0.09; 0.18]**	.	**100.0%**
**Prediction interval**						**[0.02; 0.72]**		

Heterogeneity: $I^2 = 30.6\%$, $\tau^2 = 0.6886$, $p = 0.0403$

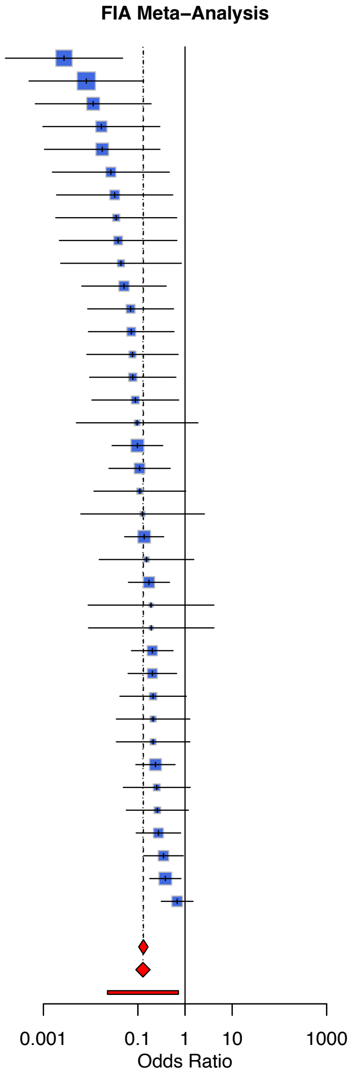

C.II The incidence of moderate to severe SIC after Duval and Tweedie revision filling.

Study	Events	Total	Events	Total	OR	95%-CI	Weight (common)	Weight (random)
Yin et al 2019	0	80	28	40	0.00	[0.00; 0.05]	0.6%	1.4%
Liu et al 2015	0	185	46	185	0.01	[0.00; 0.13]	0.7%	1.5%
Li et al 2017	0	160	17	80	0.01	[0.00; 0.19]	0.7%	1.4%
Li et al 2015	0	41	17	41	0.02	[0.00; 0.29]	0.6%	1.4%
Wang et al 2020	0	105	22	105	0.02	[0.00; 0.29]	0.7%	1.4%
Zhou et al 2025	0	50	13	49	0.03	[0.00; 0.46]	0.6%	1.4%
Liu et al 2019	0	100	13	100	0.03	[0.00; 0.55]	0.6%	1.4%
Cao(2) et al 2020	0	40	5	20	0.03	[0.00; 0.67]	0.6%	1.4%
Wang et al 2015	0	50	10	50	0.04	[0.00; 0.67]	0.6%	1.4%
He et al 2020	0	20	7	20	0.04	[0.00; 0.83]	0.6%	1.4%
Zhang et al 2024	1	60	15	60	0.05	[0.01; 0.40]	1.2%	1.9%
Tian et al 2020	1	45	11	45	0.07	[0.01; 0.57]	1.2%	1.9%
Xu et al 2014	1	50	11	50	0.07	[0.01; 0.58]	1.2%	1.9%
Cao(1) et al 2020	1	40	5	20	0.08	[0.01; 0.71]	1.1%	1.8%
Lin et al 2019	1	42	10	42	0.08	[0.01; 0.64]	1.2%	1.9%
Xu et al 2024	1	40	9	40	0.09	[0.01; 0.74]	1.2%	1.9%
Zhou et al 2014	0	30	4	30	0.10	[0.00; 1.88]	0.6%	1.4%
Gao et al 2024	3	100	24	100	0.10	[0.03; 0.34]	3.4%	2.4%
Zou et al 2019	2	110	16	110	0.11	[0.02; 0.49]	2.3%	2.3%
Zhu(2) et al 2021	1	60	4	30	0.11	[0.01; 1.03]	1.0%	1.8%
Li et al 2014	0	25	3	25	0.13	[0.01; 2.58]	0.6%	1.3%
An et al 2015	7	107	18	53	0.14	[0.05; 0.35]	5.7%	2.6%
Zhu(1) et al 2021	1	60	3	30	0.15	[0.02; 1.53]	1.0%	1.7%
Xie et al 2024	8	67	15	34	0.17	[0.06; 0.47]	5.2%	2.6%
Teng et al 2020	0	40	2	40	0.19	[0.01; 4.09]	0.6%	1.3%
Yang et al 2023	0	49	2	49	0.19	[0.01; 4.10]	0.6%	1.3%
Sun et al 2013	7	180	10	60	0.20	[0.07; 0.56]	5.1%	2.6%
Zhen et al 2019	4	50	15	50	0.20	[0.06; 0.67]	3.7%	2.5%
Li et al 2016	2	40	8	40	0.21	[0.04; 1.06]	2.0%	2.2%
Wang(1) et al 2014	2	40	4	20	0.21	[0.03; 1.27]	1.6%	2.1%
Wang(2) et al 2014	2	40	4	20	0.21	[0.03; 1.27]	1.6%	2.1%
Qian et al 2022	6	93	21	93	0.24	[0.09; 0.62]	5.7%	2.6%
You et al 2023	2	44	7	44	0.25	[0.05; 1.29]	2.0%	2.2%
Xie et al 2025	3	54	5	27	0.26	[0.06; 1.18]	2.3%	2.3%
Shen et al 2014	5	60	15	60	0.27	[0.09; 0.81]	4.4%	2.5%
Zou et al 2020	6	152	16	152	0.35	[0.13; 0.92]	5.6%	2.6%
Qian et al 2025	11	118	25	118	0.38	[0.18; 0.82]	9.0%	2.7%
Filled: Zhou et al 2014	0	30	4	30	0.63	[0.03; 12.33]	0.6%	1.4%
Ding et al 2009	14	80	19	80	0.68	[0.31; 1.48]	8.7%	2.7%
Filled: Xu et al 2024	1	40	9	40	0.69	[0.08; 5.77]	1.2%	1.9%
Filled: Lin et al 2019	1	42	10	42	0.78	[0.10; 6.45]	1.2%	1.9%
Filled: Cao(1) et al 2020	1	40	5	20	0.80	[0.09; 7.39]	1.1%	1.8%
Filled: Xu et al 2014	1	50	11	50	0.85	[0.10; 6.84]	1.2%	1.9%
Filled: Tian et al 2020	1	45	11	45	0.87	[0.11; 7.09]	1.2%	1.9%
Filled: Zhang et al 2024	1	60	15	60	1.20	[0.15; 9.46]	1.2%	1.9%
Filled: He et al 2020	0	20	7	20	1.39	[0.07; 26.49]	0.6%	1.4%
Filled: Wang et al 2015	0	50	10	50	1.60	[0.09; 28.19]	0.6%	1.4%
Filled: Cao(2) et al 2020	0	40	5	20	1.76	[0.09; 33.74]	0.6%	1.4%
Filled: Liu et al 2019	0	100	13	100	1.90	[0.11; 32.41]	0.6%	1.4%
Filled: Zhou et al 2025	0	50	13	49	2.29	[0.13; 39.72]	0.6%	1.4%
Filled: Wang et al 2020	0	105	22	105	3.48	[0.21; 58.23]	0.7%	1.4%
Filled: Li et al 2015	0	41	17	41	3.63	[0.21; 63.07]	0.6%	1.4%
Filled: Li et al 2017	0	160	17	80	5.42	[0.32; 91.43]	0.7%	1.4%
Filled: Liu et al 2015	0	185	46	185	7.57	[0.46; 123.93]	0.7%	1.5%
Filled: Yin et al 2019	0	80	28	40	22.47	[1.29; 391.98]	0.6%	1.4%
**Common effect model**		**3845**		**3189**	**0.25**	**[0.20; 0.31]**	**100.0%**	**.**
**Random effects model**					**0.23**	**[0.15; 0.36]**	**.**	**100.0%**
**Prediction interval**						**[0.01; 4.14]**		

Heterogeneity: $I^2 = 47.7\%$, $\tau^2 = 2.0229$, $p < 0.0001$

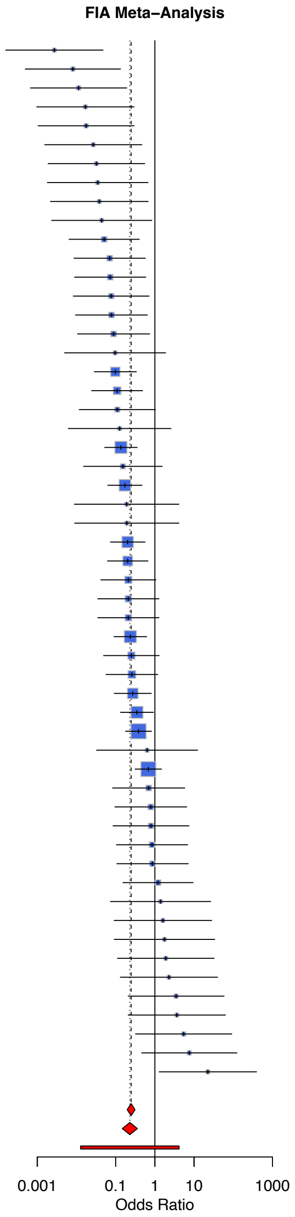

C.III The incidence of moderate to severe SIC after exclusion of outliers.

Outliers: "Yin et al 2019", "Ding et al 2009", "Qian et al 2025"

FIA Meta-Analysis

Study	Intervention Events	Intervention Total	Control Events	Control Total	OR	95%-CI	Weight (common)	Weight (random)
Liu et al 2015	0	185	46	185	0.01	[0.00; 0.13]	10.8%	1.5%
Li et al 2017	0	160	17	80	0.01	[0.00; 0.19]	5.4%	1.5%
Li et al 2015	0	41	17	41	0.02	[0.00; 0.29]	4.0%	1.4%
Wang et al 2020	0	105	22	105	0.02	[0.00; 0.29]	5.2%	1.5%
Zhou et al 2025	0	50	13	49	0.03	[0.00; 0.46]	3.1%	1.4%
Liu et al 2019	0	100	13	100	0.03	[0.00; 0.55]	3.1%	1.5%
Cao(2) et al 2020	0	40	5	20	0.03	[0.00; 0.67]	1.7%	1.4%
Wang et al 2015	0	50	10	50	0.04	[0.00; 0.67]	2.4%	1.4%
He et al 2020	0	20	7	20	0.04	[0.00; 0.83]	1.7%	1.4%
Zhang et al 2024	1	60	15	60	0.05	[0.01; 0.40]	3.4%	2.4%
Tian et al 2020	1	45	11	45	0.07	[0.01; 0.57]	2.5%	2.4%
Xu et al 2014	1	50	11	50	0.07	[0.01; 0.58]	2.5%	2.4%
Cao(1) et al 2020	1	40	5	20	0.08	[0.01; 0.71]	1.5%	2.2%
Lin et al 2019	1	42	10	42	0.08	[0.01; 0.64]	2.3%	2.4%
Xu et al 2024	1	40	9	40	0.09	[0.01; 0.74]	2.0%	2.3%
Zhou et al 2014	0	30	4	30	0.10	[0.00; 1.88]	1.0%	1.4%
Gao et al 2024	3	100	24	100	0.10	[0.03; 0.34]	5.4%	4.6%
Zou et al 2019	2	110	16	110	0.11	[0.02; 0.49]	3.6%	3.8%
Zhu(2) et al 2021	1	60	4	30	0.11	[0.01; 1.03]	1.2%	2.1%
Li et al 2014	0	25	3	25	0.13	[0.01; 2.58]	0.8%	1.3%
An et al 2015	7	107	18	53	0.14	[0.05; 0.35]	5.2%	5.8%
Zhu(1) et al 2021	1	60	3	30	0.15	[0.02; 1.53]	0.9%	2.1%
Xie et al 2024	8	67	15	34	0.17	[0.06; 0.47]	4.1%	5.6%
Teng et al 2020	0	40	2	40	0.19	[0.01; 4.09]	0.6%	1.3%
Yang et al 2023	0	49	2	49	0.19	[0.01; 4.10]	0.6%	1.3%
Sun et al 2013	7	180	10	60	0.20	[0.07; 0.56]	3.3%	5.6%
Zhen et al 2019	4	50	15	50	0.20	[0.06; 0.67]	3.2%	4.8%
Li et al 2016	2	40	8	40	0.21	[0.04; 1.06]	1.8%	3.4%
Wang(1) et al 2014	2	40	4	20	0.21	[0.03; 1.27]	1.2%	3.0%
Wang(2) et al 2014	2	40	4	20	0.21	[0.03; 1.27]	1.2%	3.0%
Qian et al 2022	6	93	21	93	0.24	[0.09; 0.62]	4.6%	5.8%
You et al 2023	2	44	7	44	0.25	[0.05; 1.29]	1.5%	3.4%
Xie et al 2025	3	54	5	27	0.26	[0.06; 1.18]	1.5%	3.7%
Shen et al 2014	5	60	15	60	0.27	[0.09; 0.81]	3.2%	5.2%
Zou et al 2020	6	152	16	152	0.35	[0.13; 0.92]	3.6%	5.8%
**Common effect model**		**2429**		**1974**	**0.11**	**[0.08; 0.14]**	**100.0%**	.
**Random effects model**					**0.12**	**[0.09; 0.17]**	.	**100.0%**
**Prediction interval**						**[0.03; 0.47]**		

Heterogeneity: $I^2 = 0.0\%$, $\tau^2 = 0.3854$, $p = 0.7702$

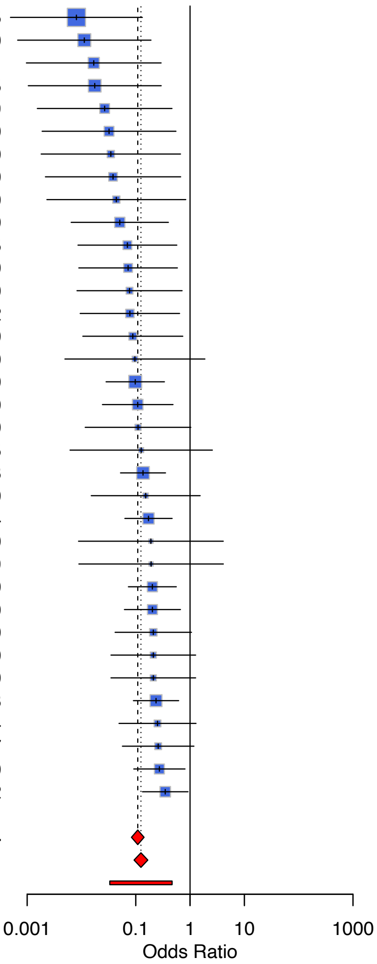

Figure S4 Network plot of the network meta-analysis for the secondary outcomes.

A. The incidence of mild SIC.

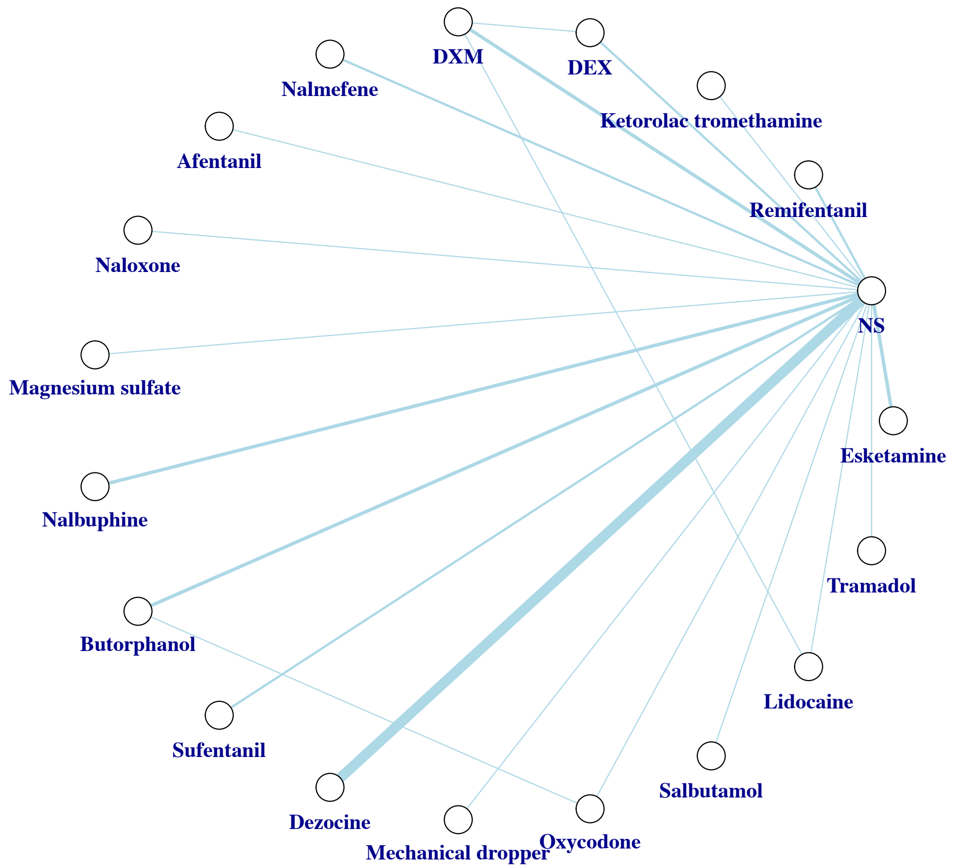

B. The incidence of moderate to severe SIC.

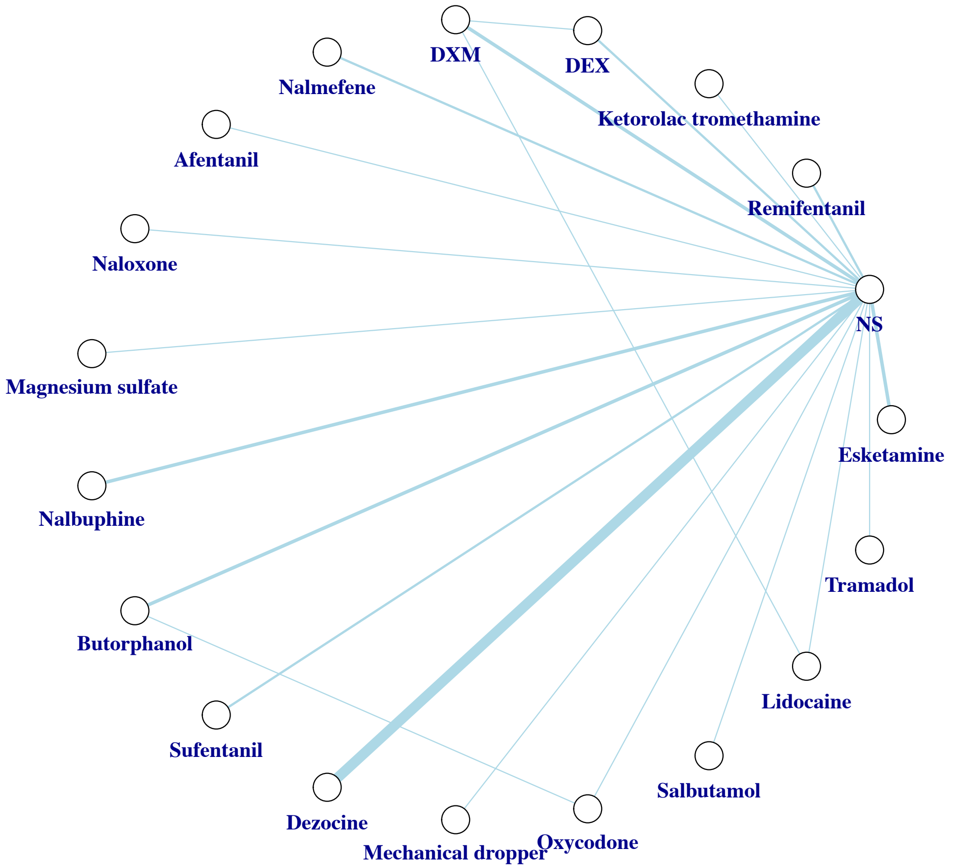

Figure S5 League table of the overall incidence of SIC (A); the incidence of mild severe SIC(B); the incidence of moderate to severe SIC(C);

A

Nalbuphine
0.67 (0.04, 8.44)	Dezocine
0.52 (0.03, 8.51)	0.79 (0.14, 4.82)	Butorphanol
0.37 (0.01, 21.78)	0.57 (0.02, 19.55)	0.72 (0.02, 26.07)	Mechanical droppe
0.35 (0.01, 20.79)	0.53 (0.01, 17)	0.68 (0.02, 26.95)	0.94 (0.01, 108.85)	Alfentanil
0.27 (0.01, 11.48)	0.41 (0.02, 8.59)	0.52 (0.03, 9.3)	0.72 (0.01, 61.97)	0.77 (0.01, 69.5)	Oxycodone
0.26 (0.01, 5.89)	0.38 (0.03, 4.92)	0.49 (0.03, 7.22)	0.69 (0.01, 37.99)	0.73 (0.01, 41.71)	0.93 (0.02, 35.72)	Remifentanil
0.16 (0, 8.46)	0.25 (0.01, 6.84)	0.32 (0.01, 10.43)	0.44 (0, 44.41)	0.47 (0, 43.39)	0.6 (0.01, 44.71)	0.66 (0.01, 34.32)	Ketorolac trometha
0.16 (0.01, 3.58)	0.24 (0.02, 2.61)	0.31 (0.02, 4.14)	0.42 (0.01, 24.1)	0.45 (0.01, 24.49)	0.59 (0.01, 21.36)	0.63 (0.02, 15.14)	0.96 (0.02, 52.97)	Nalmefene
0.15 (0, 6.56)	0.23 (0.01, 5.31)	0.29 (0.01, 8.02)	0.4 (0, 33.74)	0.43 (0.01, 36.44)	0.56 (0.01, 35.38)	0.6 (0.01, 27.99)	0.92 (0.01, 75.14)	0.94 (0.02, 39.54)	Magnesium sulfate
0.14 (0, 6.48)	0.21 (0.01, 5.53)	0.26 (0.01, 7.84)	0.37 (0, 33.01)	0.38 (0, 38.65)	0.51 (0.01, 35.05)	0.54 (0.01, 26.79)	0.82 (0.01, 79.85)	0.86 (0.02, 41.84)	0.91 (0.01, 72.85)	Salbutamol
0.14 (0.01, 2.31)	0.21 (0.02, 1.61)	0.27 (0.02, 2.65)	0.37 (0.01, 15.43)	0.39 (0.01, 17.15)	0.51 (0.01, 13.89)	0.54 (0.03, 10.08)	0.82 (0.02, 34)	0.85 (0.05, 16.03)	0.91 (0.03, 30.66)	1 (0.03, 37.38)	Esketamine
0.13 (0, 2.93)	0.2 (0.01, 2.25)	0.25 (0.02, 3.4)	0.35 (0.01, 18.24)	0.38 (0.01, 20.83)	0.48 (0.01, 16.47)	0.52 (0.02, 12.48)	0.8 (0.02, 38.93)	0.82 (0.03, 19.23)	0.86 (0.02, 35.86)	0.97 (0.02, 40.7)	0.97 (0.05, 16.11)	Sufentanil
0.11 (0, 2.08)	0.16 (0.01, 1.55)	0.21 (0.01, 2.37)	0.28 (0.01, 13.38)	0.31 (0.01, 15.31)	0.39 (0.01, 11.95)	0.42 (0.02, 8.58)	0.65 (0.01, 30.59)	0.67 (0.03, 14.65)	0.7 (0.02, 28.44)	0.78 (0.02, 33.46)	0.78 (0.05, 11.86)	0.8 (0.04, 17.58)	Dexmedetomidine
0.09 (0, 3.43)	0.14 (0.01, 3.01)	0.18 (0.01, 4.39)	0.25 (0, 19.34)	0.26 (0, 21.27)	0.34 (0, 19.99)	0.36 (0.01, 14.26)	0.55 (0.01, 43.19)	0.58 (0.01, 23.11)	0.61 (0.01, 38.89)	0.67 (0.01, 45.26)	0.67 (0.02, 20.85)	0.71 (0.02, 27.98)	0.86 (0.03, 27.54)	Lidocaine
0.09 (0, 1.55)	0.14 (0.02, 1.03)	0.17 (0.02, 1.61)	0.24 (0.01, 9.81)	0.26 (0.01, 10.53)	0.33 (0.01, 9.54)	0.35 (0.02, 6.35)	0.54 (0.01, 22.15)	0.56 (0.03, 9.93)	0.59 (0.02, 18.66)	0.66 (0.02, 23.71)	0.65 (0.05, 8.24)	0.68 (0.04, 11.78)	0.84 (0.08, 9.15)	0.98 (0.05, 19.61)	Dexamethasone
0.07 (0, 2.75)	0.11 (0, 2.12)	0.13 (0, 3.35)	0.19 (0, 13.92)	0.19 (0, 18.12)	0.25 (0, 15.37)	0.27 (0.01, 10.84)	0.41 (0.01, 32.31)	0.44 (0.01, 17.33)	0.46 (0.01, 31.75)	0.51 (0.01, 35.5)	0.51 (0.02, 14.77)	0.53 (0.01, 19.68)	0.65 (0.02, 25.15)	0.75 (0.01, 47.64)	0.78 (0.02, 22.3)	Naloxone
0.07 (0, 2.67)	0.1 (0, 2.18)	0.13 (0, 3.24)	0.18 (0, 15.45)	0.19 (0, 16.43)	0.25 (0, 14.21)	0.26 (0.01, 10.94)	0.41 (0, 34.36)	0.43 (0.01, 17.64)	0.44 (0.01, 28.94)	0.48 (0.01, 36.62)	0.49 (0.01, 15.75)	0.52 (0.01, 22.11)	0.63 (0.02, 23.68)	0.72 (0.01, 50.84)	0.76 (0.02, 23.65)	0.97 (0.01, 63.94)	Tramadol
0.02 (0, 0.2)	0.03 (0.01, 0.1)	0.04 (0.01, 0.18)	0.06 (0, 1.59)	0.07 (0, 1.67)	0.08 (0, 1.43)	0.09 (0.01, 0.84)	0.14 (0.01, 3.55)	0.14 (0.02, 1.36)	0.15 (0.01, 2.86)	0.17 (0.01, 3.75)	0.17 (0.03, 0.96)	0.17 (0.02, 1.56)	0.21 (0.03, 1.75)	0.25 (0.01, 4.56)	0.25 (0.04, 1.38)	0.33 (0.02, 6.55)	0.33 (0.02, 6.66)	Normal saline

B

Nalbuphine
0.7 (0.06, 5.66)	Dezocine
0.35 (0.02, 5.13)	0.49 (0.08, 4.35)	Butorphanol
0.27 (0.01, 7.01)	0.39 (0.02, 6.3)	0.8 (0.03, 15.34)	Mechanical droppe
0.64 (0.02, 41.32)	0.91 (0.04, 45)	1.82 (0.06, 99.48)	2.4 (0.05, 217.97)	Alfentanil
0.22 (0.01, 4.41)	0.31 (0.03, 4.22)	0.62 (0.06, 6.7)	0.78 (0.02, 27.83)	0.33 (0, 15.48)	Oxycodone
0.2 (0.01, 2.83)	0.29 (0.03, 2.63)	0.58 (0.03, 6.74)	0.75 (0.03, 17.88)	0.31 (0, 10.05)	0.95 (0.04, 18.09)	Remifentanil
0.09 (0, 2.57)	0.13 (0.01, 2.71)	0.25 (0.01, 6.24)	0.33 (0.01, 14.59)	0.14 (0, 7.78)	0.43 (0.01, 14.43)	0.44 (0.02, 14.73)	Ketorolac trometha
0.16 (0.01, 1.69)	0.22 (0.03, 1.43)	0.45 (0.03, 3.79)	0.56 (0.02, 10.77)	0.24 (0, 6.21)	0.73 (0.03, 10.85)	0.75 (0.06, 9.83)	1.68 (0.06, 42.86)	Nalmefene
0.5 (0.02, 13.34)	0.73 (0.05, 12.67)	1.49 (0.05, 30.54)	1.87 (0.05, 71.86)	0.81 (0.01, 37.61)	2.4 (0.07, 73.38)	2.53 (0.1, 70.98)	5.71 (0.13, 260.29)	3.32 (0.17, 78.8)	Magnesium sulfate
0.26 (0.01, 6.54)	0.37 (0.02, 6.52)	0.74 (0.03, 14.28)	0.92 (0.03, 35.73)	0.4 (0, 20.15)	1.19 (0.03, 37.15)	1.27 (0.05, 34.97)	2.81 (0.06, 133.41)	1.66 (0.08, 40.34)	0.49 (0.01, 19.11)	Salbutamol
0.13 (0.01, 1.13)	0.19 (0.03, 0.95)	0.38 (0.03, 2.6)	0.47 (0.02, 7.72)	0.2 (0, 4.46)	0.6 (0.03, 7.35)	0.64 (0.06, 6.97)	1.44 (0.06, 28.74)	0.84 (0.1, 7.19)	0.25 (0.01, 4.18)	0.51 (0.02, 8.43)	Esketamine
0.21 (0.01, 2.56)	0.3 (0.03, 2.27)	0.6 (0.04, 6.08)	0.76 (0.03, 15.66)	0.32 (0.01, 9.58)	0.97 (0.05, 16.19)	1.03 (0.07, 15.08)	2.34 (0.08, 59.44)	1.35 (0.12, 15.89)	0.4 (0.02, 8.79)	0.82 (0.03, 18.34)	1.61 (0.17, 14.81)	Sufentanil
0.19 (0.01, 1.98)	0.28 (0.04, 1.59)	0.56 (0.04, 4.54)	0.7 (0.03, 12.58)	0.3 (0, 7.07)	0.9 (0.05, 12.37)	0.96 (0.08, 11.17)	2.15 (0.08, 48.43)	1.25 (0.13, 12.45)	0.38 (0.02, 6.79)	0.77 (0.03, 14.02)	1.52 (0.2, 10.94)	0.93 (0.09, 9.48)	Dexmedetomidine
0.15 (0.01, 2.65)	0.22 (0.02, 2.5)	0.43 (0.02, 6.43)	0.54 (0.02, 15.35)	0.24 (0, 8.76)	0.7 (0.02, 15.85)	0.74 (0.04, 15.55)	1.67 (0.04, 55.15)	0.97 (0.06, 16)	0.3 (0.01, 8.22)	0.6 (0.02, 16.63)	1.17 (0.09, 16.29)	0.73 (0.04, 13.52)	0.78 (0.05, 10.87)	Lidocaine
0.16 (0.01, 1.44)	0.23 (0.04, 1.11)	0.47 (0.04, 3.12)	0.59 (0.03, 9.6)	0.25 (0, 5.48)	0.75 (0.04, 9.35)	0.79 (0.07, 8.22)	1.78 (0.08, 35.89)	1.02 (0.13, 9.1)	0.31 (0.02, 5.05)	0.63 (0.03, 10.33)	1.24 (0.2, 7.79)	0.77 (0.08, 7.19)	0.83 (0.15, 4.95)	1.07 (0.1, 10.82)	Dexamethasone
0.08 (0, 1.3)	0.12 (0.01, 1.19)	0.25 (0.01, 2.73)	0.31 (0.01, 7.49)	0.13 (0, 4.16)	0.39 (0.01, 7.87)	0.41 (0.02, 7.32)	0.93 (0.03, 28.43)	0.55 (0.04, 8.51)	0.16 (0.01, 4.19)	0.33 (0.01, 8.01)	0.65 (0.05, 8.17)	0.4 (0.02, 6.24)	0.43 (0.03, 6.11)	0.56 (0.02, 11.96)	0.52 (0.04, 6.06)	Naloxone
0.14 (0.01, 2.09)	0.21 (0.02, 1.96)	0.41 (0.02, 5.02)	0.52 (0.02, 13.47)	0.22 (0, 7.71)	0.66 (0.02, 13.44)	0.7 (0.04, 12.15)	1.57 (0.04, 48.49)	0.92 (0.06, 14.45)	0.28 (0.01, 6.91)	0.55 (0.02, 14.22)	1.11 (0.09, 13.57)	0.68 (0.04, 10.82)	0.74 (0.05, 9.64)	0.94 (0.04, 21.95)	0.9 (0.07, 10.03)	1.69 (0.08, 34.12)	Tramadol
0.06 (0.01, 0.3)	0.08 (0.02, 0.2)	0.16 (0.02, 0.68)	0.2 (0.01, 2.28)	0.09 (0, 1.32)	0.25 (0.02, 2.16)	0.27 (0.03, 1.8)	0.61 (0.03, 8.51)	0.35 (0.07, 1.81)	0.11 (0.01, 1.2)	0.21 (0.01, 2.41)	0.42 (0.1, 1.5)	0.26 (0.04, 1.55)	0.28 (0.06, 1.27)	0.36 (0.04, 3.17)	0.34 (0.09, 1.13)	0.64 (0.07, 5.3)	0.38 (0.04, 3.24)	Normal saline

C

Nalbuphine
0 (0, 0.12)	Dezocine
13.45 (0, 1.28e+18)	5.29e+15 (125.72, 1.475e+29)	Butorphanol
0.03 (0, 5.34e+41)	3.50e+11 (1.55, 2.00e+47)	0 (0, 3.92e+41)	Mechanical droppe
0 (0, 0.1)	0.64 (0.01, 52.05)	0 (0, 0.01)	0 (0, 0.71)	Alfentanil
0 (0, 0.16)	0.79 (0.01, 65.94)	0 (0, 0.01)	0 (0, 0.93)	1.23 (0, 418.31)	Oxycodone
0 (0, 0.11)	0.86 (0.03, 18.69)	0 (0, 0.01)	0 (0, 0.64)	1.33 (0.01, 177.06)	1.09 (0.01, 143.67)	Remifentanil
0 (0, 0.2)	0.86 (0.01, 82.62)	0 (0, 0.01)	0 (0, 1.13)	1.35 (0, 501.83)	1.1 (0, 391.72)	1.02 (0.01, 203.02)	Ketorolac trometha
0 (0, 0.06)	0.43 (0.02, 10.98)	0 (0, 0)	0 (0, 0.34)	0.67 (0, 114.08)	0.54 (0, 97.07)	0.51 (0.01, 36.06)	0.49 (0, 83)	Nalmefene
0 (0, 0.19)	1.31 (0.02, 50.06)	0 (0, 0.01)	0 (0, 1.13)	1.95 (0.01, 400.03)	1.65 (0.01, 327.87)	1.46 (0.02, 148.77)	1.45 (0, 300.4)	3.03 (0.02, 245.55)	Magnesium sulfate
0 (0, 0.03)	0.23 (0, 9.67)	0 (0, 0)	0 (0, 0.22)	0.34 (0, 77.11)	0.28 (0, 62.73)	0.25 (0, 27.75)	0.25 (0, 61.98)	0.51 (0, 43.38)	0.18 (0, 28.13)	Salbutamol
0 (0, 0.05)	0.41 (0.03, 4.61)	0 (0, 0)	0 (0, 0.28)	0.64 (0.01, 65.19)	0.51 (0, 52.72)	0.48 (0.01, 16.76)	0.47 (0, 45.01)	0.96 (0.02, 28.57)	0.32 (0.01, 23.25)	1.86 (0.03, 153.35)	Esketamine
0 (0, 0.03)	0.29 (0.01, 4.29)	0 (0, 0)	0 (0, 0.2)	0.43 (0, 44.58)	0.35 (0, 37.5)	0.32 (0.01, 13.51)	0.33 (0, 30.78)	0.66 (0.01, 23.02)	0.22 (0, 16.65)	1.28 (0.01, 107.94)	0.69 (0.02, 16.27)	Sufentanil
0 (0, 0.03)	0.23 (0.01, 3.3)	0 (0, 0)	0 (0, 0.17)	0.35 (0, 38.02)	0.29 (0, 29.2)	0.26 (0.01, 11.21)	0.26 (0, 27.87)	0.53 (0.01, 18.09)	0.18 (0, 13.15)	1.02 (0.01, 88.56)	0.56 (0.02, 13.2)	0.8 (0.02, 30.39)	Dexmedetomidine
0 (0, 0.03)	0.19 (0, 6.48)	0 (0, 0)	0 (0, 0.16)	0.29 (0, 53.35)	0.23 (0, 42.57)	0.21 (0, 17.91)	0.21 (0, 38.22)	0.43 (0, 32.65)	0.14 (0, 23.08)	0.82 (0.01, 132.25)	0.45 (0.01, 24.05)	0.66 (0.01, 54.11)	0.8 (0.01, 60.51)	Lidocaine
0 (0, 0.02)	0.14 (0.01, 1.33)	0 (0, 0)	0 (0, 0.1)	0.21 (0, 18.63)	0.18 (0, 14.31)	0.16 (0, 5.12)	0.16 (0, 13.52)	0.32 (0.01, 8.66)	0.11 (0, 6.84)	0.61 (0.01, 43.23)	0.34 (0.02, 5.82)	0.49 (0.02, 14.16)	0.61 (0.03, 12.28)	0.76 (0.02, 24.35)	Dexamethasone
0 (0, 0.02)	0.17 (0, 4.84)	0 (0, 0)	0 (0, 0.14)	0.26 (0, 45.04)	0.21 (0, 37.45)	0.2 (0, 14.83)	0.2 (0, 31.69)	0.4 (0, 24.59)	0.13 (0, 17.85)	0.76 (0, 104.02)	0.42 (0.01, 18.66)	0.6 (0.01, 43.19)	0.75 (0.01, 48.83)	0.92 (0.01, 96.35)	1.25 (0.02, 59.49)	Naloxone
0 (0, 0.02)	0.12 (0, 3.25)	0 (0, 0)	0 (0, 0.09)	0.18 (0, 28.7)	0.15 (0, 24.8)	0.13 (0, 9.74)	0.13 (0, 20.67)	0.28 (0, 16.14)	0.09 (0, 11.1)	0.52 (0, 69.88)	0.29 (0, 12.06)	0.41 (0.01, 28.44)	0.52 (0.01, 31.32)	0.63 (0, 69.93)	0.85 (0.02, 39.63)	0.69 (0.01, 75.27)	Tramadol
0 (0, 0)	0.04 (0.01, 0.13)	0 (0, 0)	0 (0, 0.02)	0.06 (0, 2.65)	0.05 (0, 2.31)	0.04 (0, 0.65)	0.04 (0, 2.03)	0.09 (0, 1.17)	0.03 (0, 0.95)	0.17 (0, 6.47)	0.1 (0.01, 0.62)	0.14 (0.01, 1.78)	0.17 (0.01, 1.95)	0.21 (0.01, 5.67)	0.28 (0.03, 1.93)	0.23 (0.01, 6.19)	0.33 (0.01, 8.71)	Normal saline

For the three outcomes above, we calculated the odds ratio (OR) and the 95% confidence interval (CI). If the OR is < 1, it means that the upper intervention is superior to the right-sided intervention. Conversely, if the OR value is > 1, the opposite is true. Accordingly, statistically significant comparisons are highlighted in green. SIC: sufentanil-induced cough.

Figure S6 Results of local inconsistency and heterogeneity analyses.

A. I Heterogeneity results for the overall incidence of SIC.

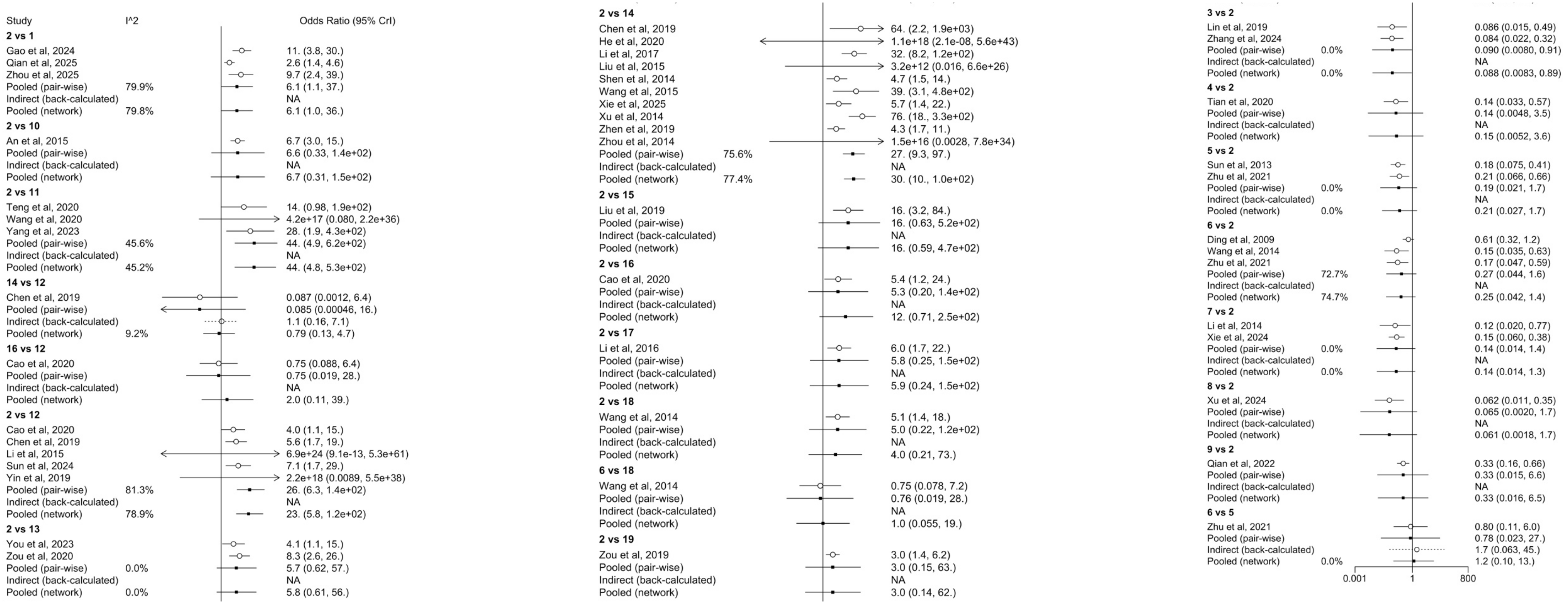

A. II Inconsistency results for the overall incidence of SIC.

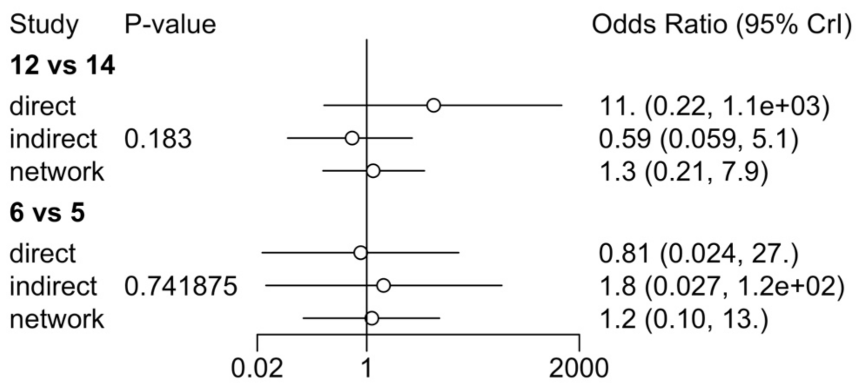

B. I Heterogeneity results for the incidence of mild SIC.

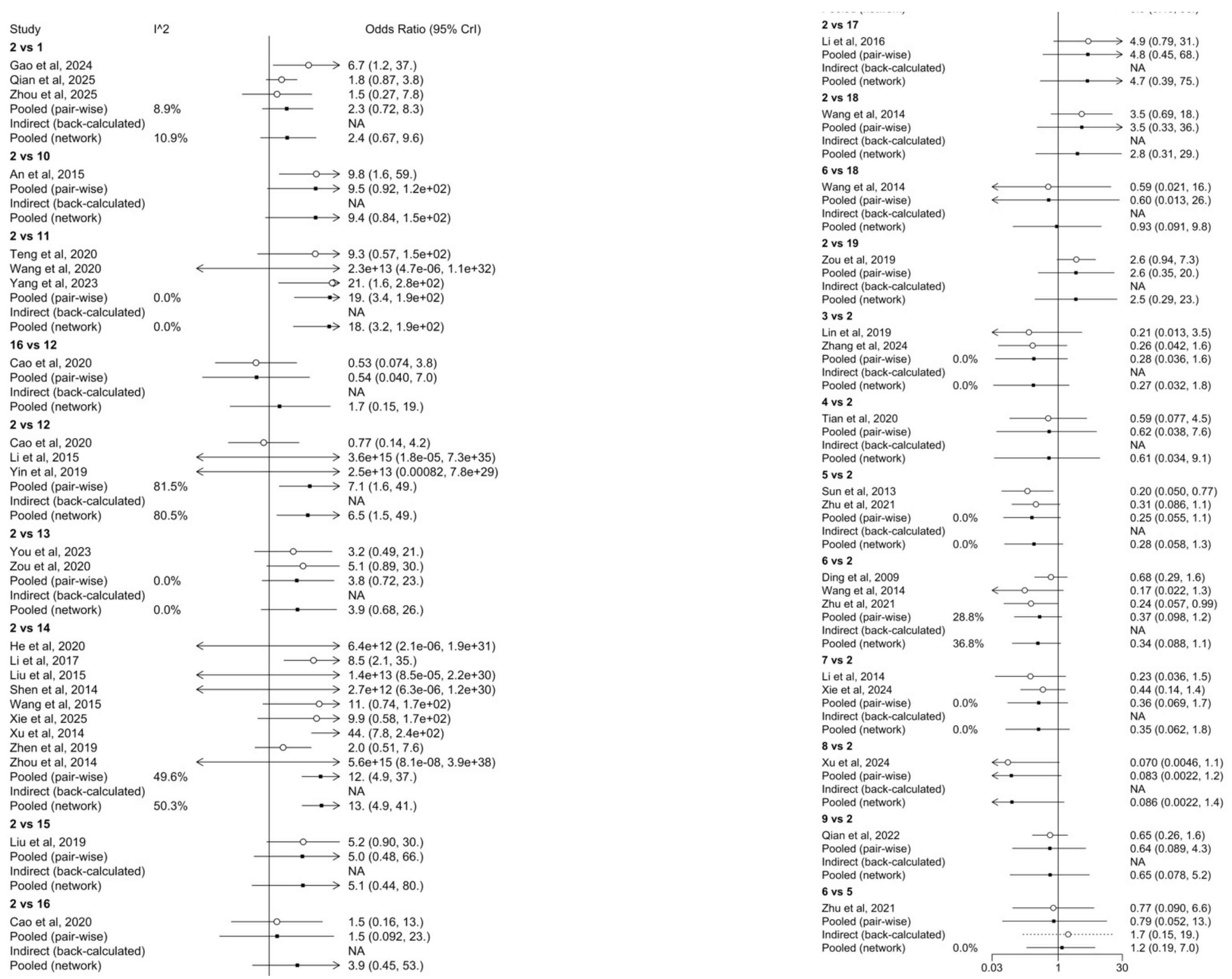

B.II Inconsistency results for the incidence of mild SIC.

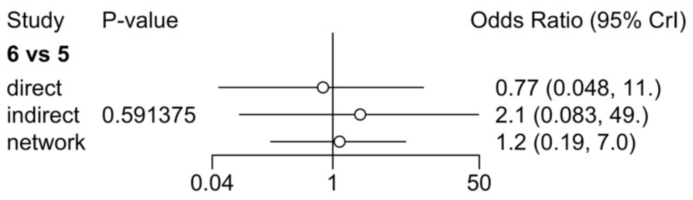

C.I Heterogeneity results for the incidence of moderate to severe SIC.

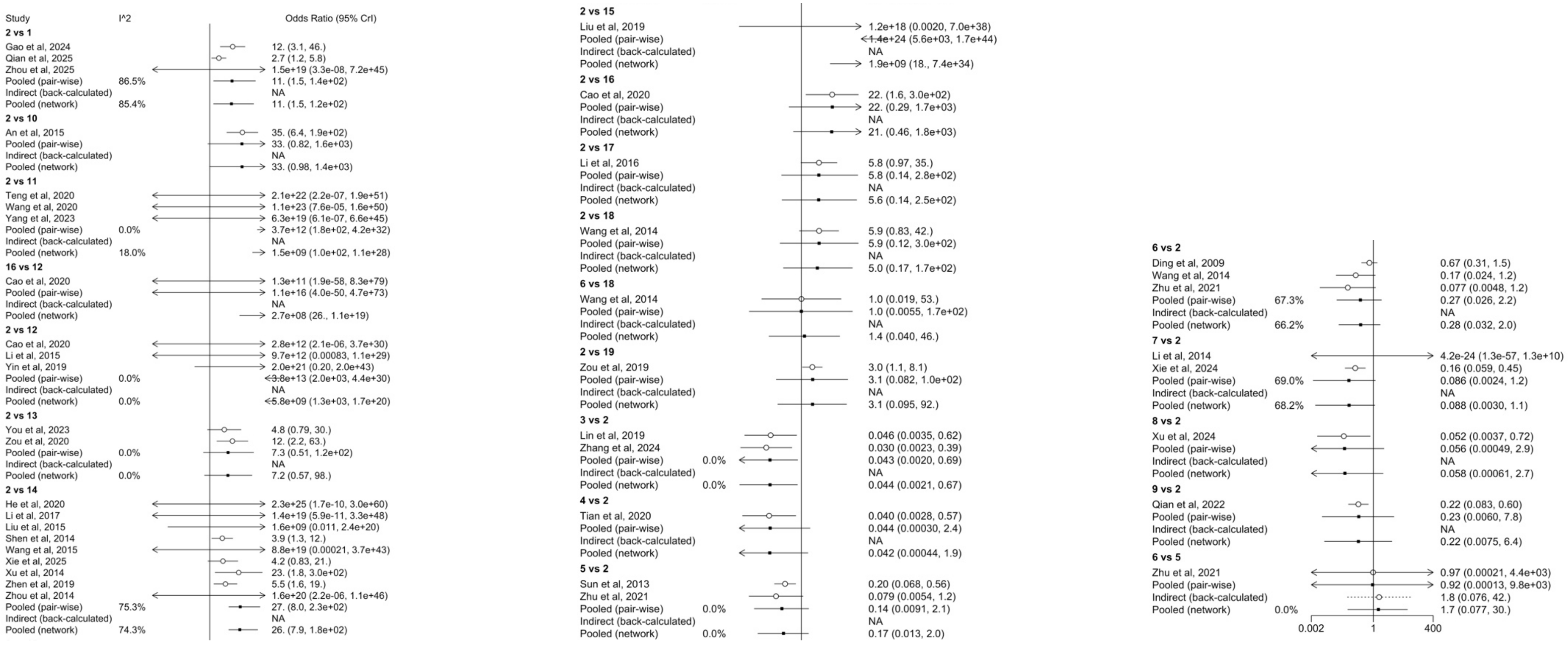

C.II Inconsistency results for the incidence of moderate to severe SIC.

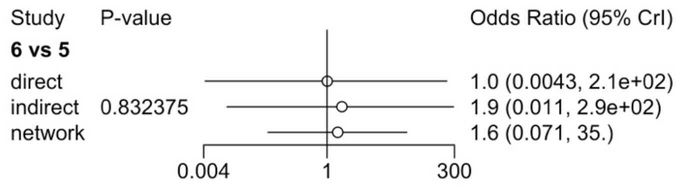

Figure S7 Gelman–Rubin diagnostic plots

Figure S7A Gelman–Rubin diagnostic plots for the overall incidence of SIC

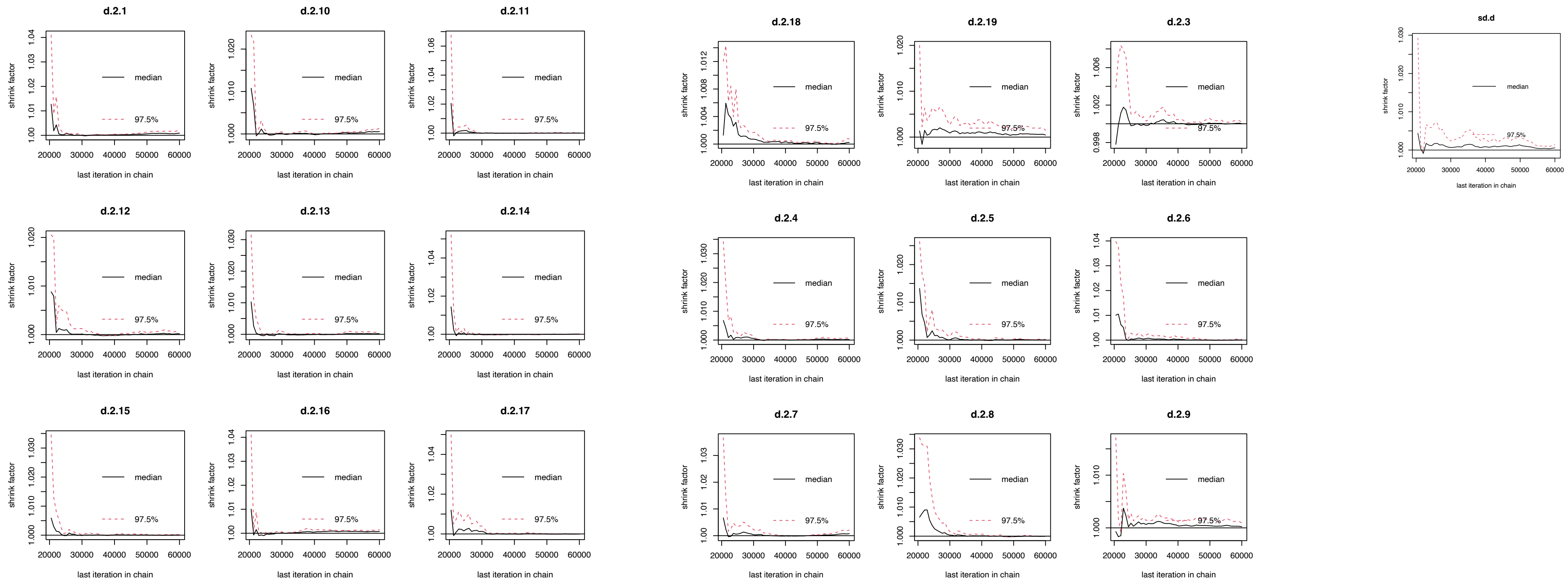

Figure S7B Gelman–Rubin diagnostic plots for the incidence of mild SIC.

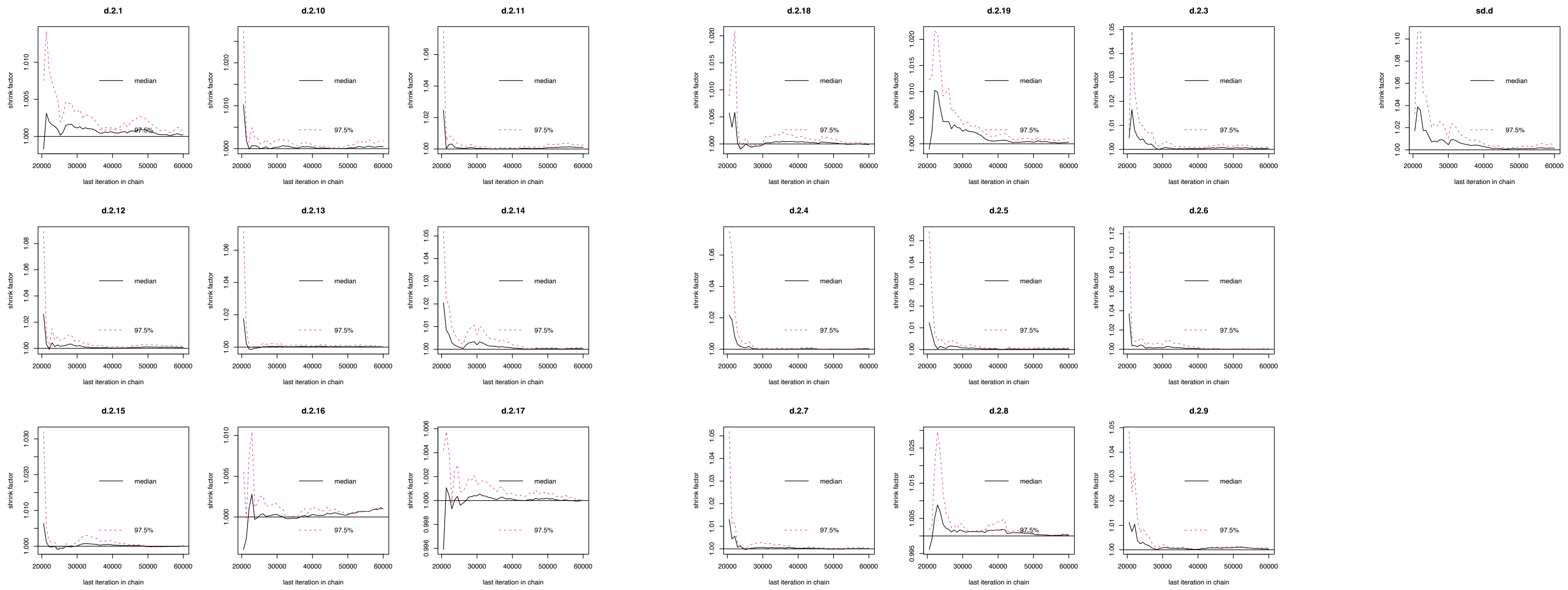

Figure S7C Gelman–Rubin diagnostic plots for the incidence of moderate to severe SIC.

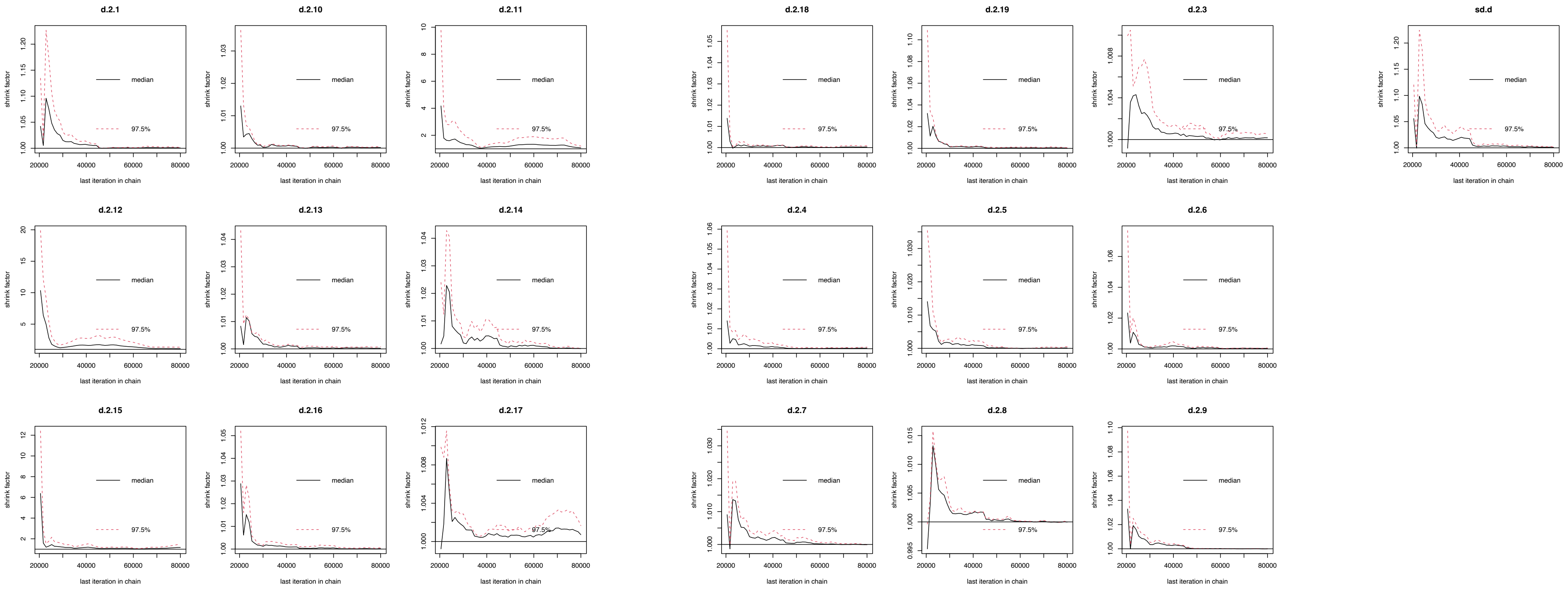

Supplement: Supplementary file 1 [file DataSheet1.pdf]
